# A Digital Therapeutic Intervention for Inpatients With Elevated Suicide Risk: A Randomized Clinical Trial

**DOI:** 10.1001/jamanetworkopen.2025.25809

**Published:** 2025-08-08

**Authors:** Craig J. Bryan, Patricia Simon, Samuel T. Wilkinson, Michael H. Allen, Jeremiah Perez, Caleb Adler, Khatiya Moon, Lauren Astorino, Kristen M. Carpenter, Luke Misquitta, Katherine Brownlowe, Lauren R. Khazem, Jarrod Hay, Austin G. Starkey, Julia Tartaglia, Helena Winston, Scott Simpson, Alecia D. Dager, Seth Feuerstein

**Affiliations:** 1 Department of Psychiatry and Behavioral Health, The Ohio State University College of Medicine, Columbus; 2 Department of Psychiatry, Yale School of Medicine, New Haven, Connecticut; 3 Oui Therapeutics Inc, New Haven, Connecticut; 4 Department of Psychiatry, University of Colorado School of Medicine, Anschutz Medical Campus, Aurora; 5 Biostatistics Department, Avania, Marlborough, Massachusetts; 6 Department of Psychiatry and Behavioral Neuroscience, University of Cincinnati College of Medicine, Cincinnati, Ohio; 7 Department of Psychiatry, Northwell Health, Glen Oaks, New York

## Abstract

**Importance:**

Suicide is a leading cause of death in the US. Suicide-specific cognitive behavior therapy (CBT) is effective for reducing suicide attempts but is difficult to implement.

**Objective:**

To evaluate the efficacy of a smartphone-based digital therapeutic intervention designed to deliver suicide-focused CBT in reducing suicidal behavior among patients hospitalized for a suicide attempt or suicidal ideation.

**Design, Setting, and Participants:**

This multisite, double-blind, randomized clinical trial was conducted in 6 psychiatric inpatient units across the US. Adult patients admitted with elevated suicide risk from April 2022 to April 2024 were included. Participants completed a baseline assessment and were randomly assigned to either the digital therapeutic group or control application group. The trial was stopped early by the Data Management Safety Board because it surpassed the prespecified futility boundary for the primary end point. Statistical analysis followed the intention-to-treat principle.

**Interventions:**

The digital therapeutic intervention includes 12 sessions of smartphone-based educational modules (lasting 10-15 minutes each) drawn from CBT for suicide prevention. The active control is a 12-session smartphone-based application that delivers safety planning and psychoeducation about suicide. For both interventions, the first session was completed prior to hospital discharge and the remaining self-paced sessions could be completed after discharge. All participants also received treatment as usual, which included suicide risk assessment, supportive listening, crisis resources, clinician assessment, safety planning, and referral to outpatient treatment.

**Main Outcomes and Measures:**

The primary end point was time (days) to first actual suicide attempt during follow-up. The secondary end points were change in suicidal ideation from baseline to week 24 (quantified as a change in the Scale for Suicide Ideation total score) and clinician-rated clinical improvement at week 24. The nonprespecified sensitivity analysis end point for suicide attempts was the rate of suicide attempts (actual, aborted, and interrupted). Prespecified subgroup analyses were also conducted to examine treatment effects among patients with vs without prior suicide attempts.

**Results:**

A total of 339 participants (mean [SD] age, 27.9 [10.7] years; 224 females [66.1%]) were included. Follow-up data were available from 266 participants (78.5%). Time to first actual suicide attempt, the primary end point, was not significantly different across treatment groups (log-rank χ^2^_1_ = 3.6; *P* = .06). Among the 170 participants with prior suicide attempts, nonprespecified sensitivity analyses indicated that the adjusted rate of follow-up suicide attempts was 58.3% lower in the digital therapeutic group than the control application group (0.70 vs 1.68 attempts per person-year; rate ratio [RR], 0.42 [95% CI, 0.18-0.95]; *P* = .04), and the odds of clinical improvement were higher in the digital therapeutic group than the control application group (97.9% vs 87.5%; odds ratio, 7.59; 95% CI, 1.14-153.62; *P* = .04). Trajectories of suicidal ideation significantly differed between the digital therapeutic and control application groups (*F*_3,206_ = 2.9, *P* = .04), with decreased suicidal ideation through week 24 in the digital therapeutic group, but in the control application group, suicidal ideation decreased through week 12 and then increased at week 24. Nonprespecified dose-response analyses indicated the suicide attempt rate among patients with a prior suicide attempt decreased by 14.0% for every digital therapeutic module completed (adjusted RR, 0.86; 95% CI, 0.76-0.98; *P* = .02).

**Conclusions and Relevance:**

In this randomized clinical trial, the digital therapeutic intervention did not change the time to first actual suicide attempt but helped to sustain reductions in suicidal ideation among inpatients with elevated suicide risk. However, among patients with prior suicide attempts, the digital therapeutic intervention helped reduce recurrent suicide attempts and increased the percentage of inpatients with clinician-rated clinical improvement.

**Trial Registration:**

ClinicalTrials.gov Identifier: NCT05144685

## Key Points

**Question:**

Does a smartphone-based digital therapeutic intervention designed to deliver suicide-focused cognitive behavior therapy (CBT) reduce future suicide attempts among patients hospitalized with acutely elevated suicide risk?

**Findings:**

In this randomized clinical trial of 339 inpatients in psychiatric hospitals, no difference was found in time to first actual suicide attempt between those who used the digital therapeutic intervention and those who used the control application.

**Meaning:**

The digital therapeutic intervention did not impact time to first suicide attempt after discharge among patients admitted for suicidal ideation and/or suicide attempts.

## Teaser

This randomized clinical trial evaluates the efficacy of a smartphone-based educational intervention in reducing suicidal behavior after discharge of patients hospitalized for a suicide attempt or suicidal ideation.

## Introduction

Suicide is one of the leading causes of death in the US and has increased by more than 33% since 1999.^1^ More than 1 million adults per year are estimated to engage in nonfatal suicidal behavior.^2^ The weeks and months after discharge from psychiatric hospitalization are among periods with the highest risk for suicide mortality,^3^ highlighting the need for interventions that can reduce the recurrence of suicide attempts in this group.

Suicide-focused cognitive behavior therapies (CBTs) have been shown to reduce suicidal behaviors by approximately 20% on average compared with other mental health treatments.^4,5^ Newer-generation manualized CBT protocols that focus directly on suicidal thoughts and behaviors are even more effective, often reducing suicidal behaviors by 40% or more among patients reporting recent suicidal thoughts and behaviors.^6,7,8,9^ A recent randomized clinical trial (RCT) further showed that a 4-session suicide-focused CBT protocol adapted for psychiatric inpatient settings reduced postdischarge suicide attempt rates by greater than 60% compared with treatment as usual (TAU).^10^ Despite their efficacy, suicide-focused CBTs have not been widely adopted by mental health professionals and are often poorly implemented in routine practice, reducing their effectiveness. Digital therapeutics—patient-facing software applications designed to help patients prevent, manage, or treat a medical condition^11^—offer a promising solution to these barriers by providing immediate, scalable, and cost-effective access to evidence-based interventions delivered with high fidelity.

Because the risk of suicidal behaviors is so high following psychiatric hospitalization, suicide-focused digital therapeutics may be especially impactful when initiated around the time of discharge. Guided by this possibility, this study aimed to evaluate the effectiveness of a smartphone-based digital therapeutic application designed to deliver suicide-focused CBT for the reduction of suicidal behavior among patients hospitalized for a suicide attempt or suicidal ideation. We hypothesized statistically significant differences in the time to first suicide attempt (primary end point), suicidal ideation (secondary end point), and clinical improvement (secondary end point) after randomization that favored the digital therapeutic intervention over an active control application.

## Methods

### Study Design and Settings

This multisite, double-blind RCT was conducted at 6 inpatient psychiatric units across the US. These 6 units were diverse in size, geographic location, and communities served (eTable 1 in Supplement 2). The Western-Copernicus Group Institutional Review Board approved the trial, and the protocol is available in Supplement 1. All participants provided informed consent. We followed the Consolidated Standards of Reporting Trials (CONSORT) reporting guideline.^12^

Patients were recruited from April 2022 to April 2024 during their inpatient psychiatric stay. After consenting to participate, eligible patients completed a baseline assessment and were randomly assigned to either the OTX-202 application group (hereafter digital therapeutic group) or the control application group. A computerized stratified block randomization procedure, which was developed by an independent biostatistician (J.P.), was used with the following strata: study site, sex (male or female), race and ethnicity (American Indian or Alaska Native, Asian, Black or African American, Hispanic or Latino, Native Hawaiian or Other Pacific Islander, White, or other), and lifetime history of suicide attempts (0, 1, or ≥2). Self-reported race and ethnicity were assessed in this RCT because suicide rates in the US differ across racial and ethnic groups.^13^ Participants were enrolled and assigned to treatment groups by research assistants who were not involved in follow-up data collection.

### Participants

Adult inpatients (aged ≥18 years) in psychiatric units were eligible to participate if they (1) had been hospitalized for a suicide attempt or suicidal ideation with the intent to harm themselves, as indicated by a total score of 5 or higher on the Scale for Suicide Ideation (SSI); (2) owned a smartphone capable of downloading and running the intervention applications; and (3) were able and willing to provide at least 2 verifiable emergency contacts. Inpatients were excluded if they (1) had acutely impaired mental status that precluded their capacity to provide informed consent (eg, uncontrolled psychosis or mania, or under the influence of alcohol or other substances), (2) had cognitive impairments or medical conditions that could adversely affect the integrity of the data, or (3) were concurrently enrolled in another clinical trial.

### Interventions

All participants received TAU during and after enrollment in addition to their randomly assigned intervention. TAU included suicide risk assessment, supportive listening, crisis resources, clinician assessment, safety planning, and referral to outpatient treatment. Participants downloaded 1 of 2 randomly assigned intervention applications to their smartphones with the assistance of a researcher (including J.H., A.G.S., and L.A.) and then completed the application’s first module. Questions about how to use the application were answered during the initial onboarding. Following hospital discharge, participants accessed and used their assigned application at their desired frequency and pace. Participants were informed that they would be randomly assigned to 1 of 2 digital interventions that included some combination of supportive listening, education about crisis services, software services, and referral to treatment services. Participants were not told to which application they were assigned.

#### Digital Therapeutic Intervention Plus TAU

The digital therapeutic application includes 12 educational modules (lasting 10-15 minutes each) drawn from CBT for suicide prevention, an individual manualized psychotherapy that has been shown to substantially reduce suicide attempts.^6,7,8,14^ The first module focuses on crisis response planning, lethal means restriction, and psychoeducation about suicidal behavior. Subsequent modules teach emotion regulation and cognitive reappraisal skills. The digital therapeutic application’s content is delivered through a chatbot, narration videos, and actor portrayals of the lived experience of actual patients who have completed the treatment. Patients may progress to the next module only after completing the psychoeducation and skills practice assignments in the preceding module. Additional details about the development and design of the digital therapeutic application, including example screenshots, are available in the eMethods in Supplement 2.

#### Control Application Plus TAU

The control application included information and materials that are commonly provided to patients receiving TAU, such as safety planning, psychoeducational material about mental health symptoms and diagnoses, encouragement to adhere to clinician recommendations, and referrals to other professional resources. The control application was designed to mimic the digital therapeutic application’s user interface and engagement but did not include content focused on emotion regulation or cognitive reappraisal skills training.

### Outcomes

All outcomes were assessed by independent evaluators who were masked to treatment assignments. Follow-up assessments were conducted via remote interview at 4, 8, 12, 24, 52, 78, and 104 weeks after baseline. The study met stopping criteria before participants reached week 104. Fatal and nonfatal suicide attempts (actual, aborted, and interrupted) during follow-up were assessed using the Columbia Suicide Severity Rating Scale,^15^ change in suicidal ideation was assessed using the SSI (score range: 0-38, with higher scores indicating more severe suicidal ideation),^16^ change in clinical condition was assessed using the Clinical Global Impression for Severity of Suicidality (CGI-SS; score range: 0 [very much improved] to 7 [very much worse]),^17,18^ and patient perceptions of their intervention were assessed using the Credibility Expectancy Questionnaire (CEQ) items 1 to 3 only (score range: 3-27, with higher scores indicating more favorable perceptions of the intervention).^19^ Adverse events (AEs) were assessed using an AE reporting form at the follow-up assessments. Research staff asked participants, “Have you had any physical or mental health problems since our last call?” AEs were subsequently rated for severity and their relationship to intervention use. AEs were coded using the standardized MedDRA (Medical Dictionary for Regulatory Activities), version 25.1 or higher.

The prespecified primary end point was time to first actual suicide attempt (excluding aborted and interrupted attempts), quantified as number of days after randomization. The prespecified secondary end points were change in suicidal ideation (quantified as a change in SSI total score) from baseline to week 24 and clinician-rated clinical improvement at week 24.

### Efforts to Minimize Bias

To minimize bias, participants, investigators, and outcome assessors were masked to treatment assignments. Outcome assessments were conducted by independent evaluators, audio recorded, and overseen by a centralized assessment team who reviewed randomly selected recordings and met monthly with the assessors to provide feedback. Participants were also instructed not to talk about their assigned application with the independent evaluators. The control condition included a smartphone application designed to look and operate as a digital therapeutic application.

### Statistical Analysis

The statistical analysis plan can be found in Supplement 1. The sample size was estimated based on the log-rank test to compare time to first actual suicide attempt (primary end point). The log-rank test accounts for dropouts and censored data,^20,21^ consistent with the intention-to-treat principle. Based on previously published RCTs,^6,7,8^ we assumed the probability of event at 12 months as approximately 5.1% in the digital therapeutic group and 12.9% in the control application group. Thus, based on an exponential survival distribution, the assumed hazard rate (parameter) of the exponential distribution was 0.0044 for the digital therapeutic group and 0.0115 for the control application group, with a hazard ratio (HR) of 0.3826 (ie, 62% relative rate reduction). Assuming an accrual period of 12 months and a total study duration of 24 months, the expected number of events over the study period was approximately 37 in the control application group, 15 in the experimental group, and 52 in total. Using a 2-sided log-rank test at α = .05, we found that 391 enrolled participants randomized 1:1 provided 90% power to detect this difference with 3 planned interim analyses occurring when 60% (31), 75% (39), and 90% (47) of the anticipated number of suicide attempts had occurred. The O’Brien-Fleming^22^ spending function was used to determine the effectiveness and nonbinding futility boundaries for the analyses.

The prespecified analyses for the primary end point—time to first actual suicide attempt after randomization—were performed with survival analyses using the log-rank test followed by a Cox proportional hazards regression model to assess the robustness of findings in the presence of important covariates. Analyses were adjusted for age, borderline personality disorder diagnosis, and number of lifetime actual suicide attempts as prespecified covariates because they are known correlates of suicidal behavior.^23^ The prespecified analysis for a secondary end point—change in severity of suicidal ideation from baseline to week 24—was performed with mixed-effects modeling, with fixed effects of treatment group, time, site, baseline score, age, borderline personality disorder diagnosis, number of lifetime actual suicide attempts, baseline-score-by-time interaction, and the treatment-by-time interaction with an unstructured covariance structure to model within-subject errors. Subgroup analyses by suicide attempt history were prespecified to examine differences in treatment effects among clinically relevant subgroups.

To examine treatment effects on a broader spectrum of suicide attempts (including actual, interrupted, and aborted) as well as the frequency of suicide attempts per person-year, nonprespecified sensitivity analyses of the primary end point were conducted using zero-inflated negative binomial (ZINB) regression.^24^ ZINB models were used because the distribution of suicide attempts was characterized by an excess of 0 counts (79% of patients had no follow-up attempts) and overdispersion (mean [SD], 0.46 [2.46]). ZINB regression assumed that patients without follow-up suicide attempts represent a mixture of 2 groups: those who did not attempt suicide because they were not at risk of doing so and those who did not attempt suicide despite being at risk. This approach aligns with previous findings^3,10,25,26,27,28^ that most inpatients in psychiatric units will not attempt suicide after discharge. Consistent with this assumption, ZINB models included a zero-inflation part that compared the number of patients in each group who were not at risk of attempting suicide and a count part that compared the rate of suicide attempts by patients who were at risk. A logit link function was used for the zero-inflation part and a log link function for the count part. For patients with no prior suicide attempts, we used standard (ie, noninflated) negative binomial regression instead of ZINB because none of these patients had more than 1 follow-up suicide attempt. To evaluate dose-effect relationships between intervention completion and frequency of suicide attempts per person-year, nonprespecified exploratory analyses were also conducted separately for each treatment condition and subgroup. In all regression models, the log of follow-up time—calculated as the number of years from enrollment to final data collection—was added as a participant-level offset variable to account for differential follow-up time.

To assess the probability of improved clinical condition across treatments at week 24 (the other secondary end point), we used logistic regression with dichotomized change in CGI-SS scores (ie, improved vs not improved). Age and number of lifetime actual suicide attempts were included as covariates. Borderline personality disorder diagnosis was omitted because it caused estimation errors.

Intervention use and patient perceptions of intervention were compared using the Wilcoxon rank sum test and paired, 2-tailed *t* test. AEs were summarized using descriptive statistics (ie, frequency and percentages). All analyses were conducted using SAS, version 9.4 (SAS Institute).

Three interim analyses were planned to stop the study early in the event of demonstrated efficacy overwhelmingly favoring the digital therapeutic intervention or futility based on the primary end point. The first interim analysis occurred after 31 suicide attempts had been documented, at which time 339 of 391 participants (86.7%) were enrolled. The first interim analysis surpassed the log-rank test’s prespecified futility boundary and stopping rule. Therefore, the trial’s Data Safety Monitoring Board recommended stopping enrollment on April 22, 2024. The results used all of the data collected up to the date when the stopping criteria were met.

## Results

### Participant Characteristics

**Figure 1.**
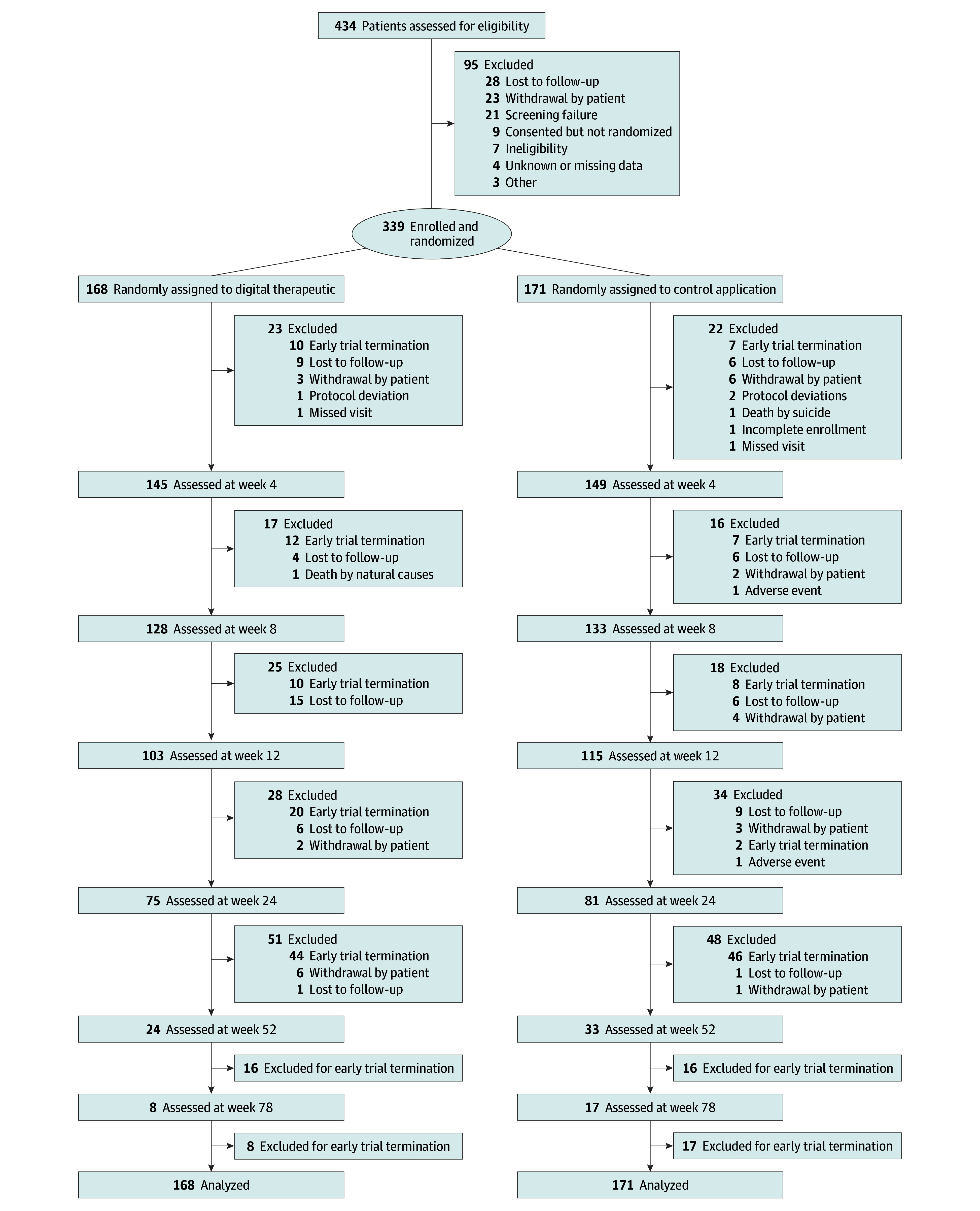
Flow of Participants Through the Study

Of 434 patients screened for eligibility, 339 were enrolled in the study (Figure 1). These patients had a mean (SD) age of 27.9 (10.7) years and included 224 females (66.1%) and 115 males (33.9%). Among them, 4 (1.2%) self-identified as American Indian or Alaska Native, 25 (7.4%) as Asian, 47 (13.9%) as Black or African American, 65 (19.2%) as Hispanic or Latino, 3 (0.9%) as Native Hawaiian or Other Pacific Islander, 228 (67.3%) as White, and 26 (7.7%) as other race and ethnicity (Table 1).

**Table 1.** Sample Characteristics

Variable	Participants, No. (%)		
	Overall (n = 339)	Digital therapeutic group (n = 168)	Control application group (n = 171)
Age, y			
Mean (SD)	27.9 (10.7)	26.6 (9.2)	29.1 (12.0)
Median (range)	24 (18-76)	24 (18-69)	24 (18-76)
Sex			
Female	224 (66.1)	111 (66.1)	113 (66.1)
Male	115 (33.9)	57 (33.9)	58 (33.9)
Ethnicity^a^			
Hispanic or Latino	65 (19.2)	33 (19.6)	32 (18.7)
Not Hispanic or Latino	270 (79.6)	132 (78.6)	138 (80.7)
Unknown or not reported	4 (1.2)	3 (1.8)	1 (0.6)
Race^a^^,^^b^			
American Indian or Alaska Native	4 (1.2)	1 (0.6)	3 (1.8)
Asian	25 (7.4)	12 (7.1)	13 (7.6)
Black or African American	47 (13.9)	24 (14.3)	23 (13.5)
Native Hawaiian or Other Pacific Islander	3 (0.9)	1 (0.6)	2 (1.2)
White	228 (67.3)	114 (67.9)	114 (66.7)
Unknown or not reported	6 (1.8)	4 (2.4)	2 (1.2)
Other^c^	26 (7.7)	12 (7.1)	14 (8.2)
Psychiatric diagnosis^d^			
Major depressive disorder	246 (72.6)	124 (73.8)	122 (71.3)
Anxiety disorder	180 (53.1)	89 (53.0)	91 (53.2)
PTSD	90 (26.5)	45 (26.8)	45 (26.3)
Sleep-wake disorder	76 (22.4)	40 (23.8)	36 (21.1)
Borderline personality disorder	69 (20.4)	43 (25.6)	26 (15.2)
Bipolar disorder	65 (19.2)	30 (17.9)	35 (20.5)
ADHD	63 (18.6)	29 (17.3)	34 (19.9)
SUD	45 (13.3)	18 (10.7)	27 (15.8)
Lifetime history of suicide attempts^e^^,^^f^			
0	120 (35.4)	58 (34.5)	62 (36.5)
1	88 (26.0)	45 (26.8)	43 (25.3)
≥2	130 (38.3)	65 (38.7)	65 (38.2)

Abbreviations: ADHD, attention-deficit/hyperactivity disorder; PTSD, posttraumatic stress disorder; SUD, substance use disorder.

^a^ Race and ethnicity data were self-reported.

^b^ Participants could report more than 1 race; thus, the numbers may be greater than the total.

^c^ Other race was unspecified. Other was an option for participants to select.

^d^ Diagnoses for less than 10% of the full sample are not reported.

^e^ Based on actual suicide attempts only (excluding aborted and interrupted suicide attempts).

^f^ Lifetime suicide attempt data were missing from 1 participant in the control application group.

### Intervention Use and Perceived Credibility

Compared with participants assigned to the control application group (n = 171), participants assigned to the digital therapeutic group (n = 168) completed fewer application sessions (mean [SD] number, 4.4 [3.8] vs 5.9 [4.8]; *Z* = 2.4; *P* = .02). The first application module was completed by 149 patients (88.7%) in the digital therapeutic group vs 156 patients (91.2%) in the control application group, at least 6 modules were completed by 52 patients (31.0%) and 80 patients (46.8%) in each group, respectively, and all 12 modules were completed by 21 patients (12.5%) and 53 patients (31.0%), respectively (eTable 2 in Supplement 2). Mean (SD) CEQ scores, assessed at week 12, did not differ between the digital therapeutic and control application groups (18.5 [5.6] vs 16.9 [6.9]; *t*_181_ = 0.7; *P* = .16), indicating that patients did not perceive 1 intervention as more credible than the other.

### Primary End Point

**Figure 2.**
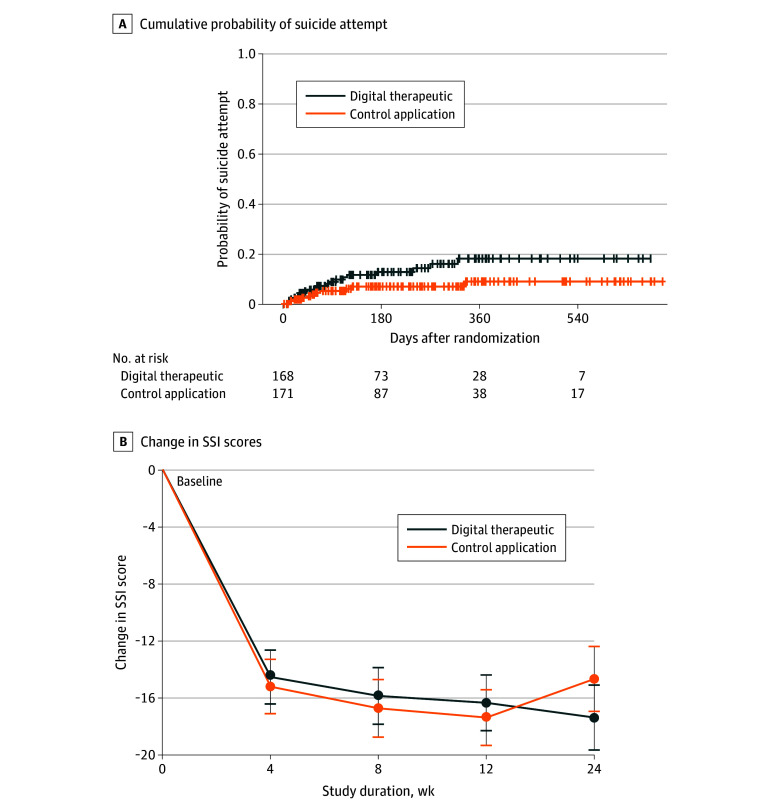
Cumulative Probability of Suicide Attempt Over Time and Mean Change in Suicidal Ideation From Baseline in Digital Therapeutic and Control Application Groups Suicidal ideation assessed with the Scale for Suicide Ideation (SSI; score range: 0-38, with higher scores indicating more severe suicidal ideation). Error bars represent 95% CIs.

A total of 266 participants (78.5%) completed at least 1 follow-up assessment by the time of the first planned interim analysis. Of 266 patients, 31 (11.7%) made at least 1 actual suicide attempt during follow-up: 20 (15.7%) in the digital therapeutic group and 11 (7.9%) in the control application group. Time to first actual suicide attempt did not significantly differ between treatment groups in either the unadjusted log-rank model (χ^2^_1_ = 3.6; *P* = .06; HR, 2.01 [95% CI, 0.96-4.19]) or adjusted Cox proportional hazards regression model (HR, 1.94 [95% CI, 0.92-4.08]; *P* = .08). The Kaplan-Meier estimate of the cumulative probability of suicide attempt at 12 months was 18.3% in the digital therapeutic group and 9.0% in the control application group (difference, 9.2%; 95% CI, –0.7% to 19.2%) (Figure 2). Restricted mean (SD) survival times at 12 months were 322.1 (9.0) days in the digital therapeutic group and 342.9 (6.6) days in the control application group.

### Sensitivity Analysis

Of 266 participants, 40 (15.0%) made at least 1 actual, aborted, or interrupted suicide attempt during follow-up: 21 (16.5%) in the digital therapeutic group and 19 (13.7%) in the control application group. Among the 40 patients who reported suicide attempts, 24 (60.0%) made 1 attempt and 16 (40.0%) made 2 or more attempts. A total of 83 suicide attempts occurred during follow-up: 34 in the digital therapeutic group and 49 in the control application group. Of these 83 suicide attempts, 78 (94.0%) were by participants with prior suicide attempts (n = 170) and 5 (6.0%) were made by participants without prior suicide attempts (n = 96) (Table 2). The unadjusted and adjusted rates of follow-up suicide attempts from the ZINB model were not significantly different across treatment groups (unadjusted vs adjusted rate ratio [RR], 0.58 [95% CI, 0.19-1.70] vs 0.62 [95% CI, 0.27-1.42]; *P* = .26).

**Table 2.** Follow-Up Suicide Attempts Overall and by Lifetime History of Suicide Attempt Across Treatment Groups of Participants With Evaluable Follow-Up Data

Group	Full sample			With prior suicide attempts			Without prior suicide attempts		
	Overall (N = 266)	Digital therapeutic group (n = 127)	Control application group (n = 139)	Overall (n = 170)	Digital therapeutic group (n = 84)	Control application group (n = 86)	Overall (n = 96)	Digital therapeutic group (n = 43)	Control application group (n = 53)
**Total No. of suicide attempts**									
All	83	34	49	78	32	46	5	2	3
Actual	34	17	17	33	16	17	1	1	0
Interrupted	13	5	8	12	5	7	1	0	1
Aborted	36	12	24	33	11	22	3	1	2
**Unadjusted rate of suicide attempts per person-year**									
All	0.40	0.36	0.43	0.60	0.51	0.69	0.06	0.06	0.06
Actual	0.16	0.18	0.15	0.25	0.25	0.26	0.01	0.03	0.00
Interrupted	0.06	0.05	0.07	0.09	0.08	0.11	0.01	0.00	0.02
Aborted	0.17	0.13	0.21	0.25	0.17	0.33	0.04	0.03	0.04
**Adjusted rate of suicide attempts per person-year^a^**									
All	NA	0.75	1.21	NA	0.70	1.68	NA	0.03	0.04
Actual	NA	0.45	0.71	NA	0.33	0.30	NA	0.01	0.00
Interrupted	NA	0.37	0.48	NA	0.25	0.47	NA	0.00	0.00
Aborted	NA	0.33	1.07	NA	0.44	1.85	NA	0.00	0.00

Abbreviation: NA, not applicable.

^a^ Adjusted for age, borderline personality disorder diagnosis, and number of lifetime actual suicide attempts.

Among patients with prior suicide attempts, the zero-inflated portion of the ZINB model indicated the odds of a participant having no follow-up suicide attempts did not significantly differ across groups (odds ratio [OR], 0.06; 95% CI, 0.001-2.48; *P* = .14). The adjusted count portion of the ZINB model significantly differed across treatment groups (adjusted RR, 0.42; 95% CI, 0.18-0.95; *P* = .04), indicating the digital therapeutic group had a 58.3% adjusted relative rate reduction in repeat suicide attempts compared with the control application group (0.70 vs 1.68 attempts per person-year). Among the participants with no prior suicide attempts, the adjusted rate of suicide attempts did not significantly differ across digital therapeutic and control application groups (RR, 0.73; 95% CI, 0.12-4.44; *P* = .74; 0.03 attempts per person-year vs 0.04 attempts per person-year).

The association between completed number of application modules and follow-up suicide attempts is reported in Table 3 by treatment group and lifetime history of suicide attempt. The only significant association was for the count portion of the ZINB model among patients with prior suicide attempts in the digital therapeutic group (adjusted RR, 0.86; 95% CI, 0.76-0.98; *P* = .02), indicating the suicide attempt rate among these patients decreased by 14.0% with each additional digital therapeutic module completed.

**Table 3.** Association Between Completed Modules and Follow-Up Suicide Attempts Across Treatment Groups by Lifetime Suicide Attempt History

Group	Zero-inflated^a^		Count^a^	
	OR (95% CI)	*P* value	RR (95% CI)	*P* value
**Patients with prior suicide attempts**				
Digital therapeutic	0.50 (0.23-1.10)	.08	0.86 (0.76-0.98)	.02
Control application	0.15 (0.001-999.99)	.96	1.06 (0.93-1.21)	.40
**Patients without prior suicide attempts**				
Digital therapeutic	NA	NA	1.14 (0.84-1.54)	.41
Control application	NA	NA	0.93 (0.68-1.26)	.12

Abbreviations: NA, not applicable; OR, odds ratio; RR, rate ratio.

^a^ Adjusted for age, borderline personality disorder diagnosis, and number of lifetime actual suicide attempts.

### Secondary End Points 

A statistically significant group-by-visit interaction (unadjusted *F*_3,206_ = 2.8, *P* = .04; adjusted *F*_3,206_ = 2.9, *P* = .04) indicated that the pattern of postbaseline change in SSI scores differed between treatment groups (Figure 2). In the digital therapeutic group, SSI scores decreased from baseline to week 4 and continued to decline through week 24. In the control application group, SSI scores decreased from baseline to week 4, continued to decline through week 12, and then increased from week 12 to week 24. From baseline to week 24, change in SSI scores in the digital therapeutic group did not significantly differ from change in SSI scores in control application (difference, –17.4 [95% CI, –19.7 to −15.1] vs –14.7 [95% CI, –16.9 to −12.4]; *t*_185_ = 2.0; *P* = .053). Similar patterns were observed in subgroup analyses, but the group-by-visit interactions were not statistically significant (with prior suicide attempts: *F*_3,128_ = 1.8, *P* = .15; without prior suicide attempts: *F*_3,72_ = 1.7, *P* = .17).

A total of 154 of 339 participants (45.4%) had CGI-SS change scores at the week-24 assessment. The percentage of participants showing clinical improvement from baseline was not significantly different across treatment groups (OR, 2.84; 95% CI, 0.80-13.29; *P* = .11): 95.8% in the digital therapeutic group vs 89.2% in the control application group. Among the participants with prior suicide attempts, the odds of clinical improvement from baseline were greater in the digital therapeutic group than the control application group (OR, 7.59; 95% CI, 1.14-153.62; *P* = .04): 97.9% vs 87.5%. Among participants with no prior suicide attempts, the odds of clinical improvement from baseline did not differ across treatment groups (OR, 0.76; 95% CI, 0.10-6.56; *P* = .79): 91.7% in the digital therapeutic group vs 91.4% in the control application group.

### Suicide Deaths and Device Safety

One suicide death occurred during the study in the control condition. There were no suicide deaths in the digital therapeutic group.

The number, severity, and nature of reported AEs were similar across groups (eTables 3-5 in Supplement 2). In the digital therapeutic group, 40 of 198 AEs (20.2%) were rated severe and 5 (2.5%) were rated as possibly or definitely related to device use (2 suicidal ideation, 1 suicide attempt, 1 missed visit, and 1 device malfunction). In the control application group, 36 of 200 AEs (18.0%) were rated severe and 5 (2.5%) were rated as possibly related to device use (2 nonsuicidal self-injury, 1 suicidal ideation, 1 suicide attempt, and 1 anxiety).

## Discussion

To our knowledge, this RCT was the first to test a digital therapeutic designed to prevent suicidal behavior. We found no difference between treatment groups for time to first actual suicide attempt (the primary end point), but we found different patterns of change in suicidal ideation (a secondary end point) from baseline to week 24. Suicidal ideation decreased in both treatment groups from baseline to week 12, continued to decline from week 12 to week 24 in the digital therapeutic group, but increased from week 12 to week 24 in the control application group, suggesting that the digital therapeutic application helped sustain reductions in suicidal ideation. Group differences favoring the digital therapeutic application were found in the sensitivity analysis examining suicide attempt rate and clinical improvement (another secondary end point) but only among patients who had suicide attempts prior to enrollment.

Strengths of this RCT include the use of a low-touch and interactive intervention, an active control group, broad inclusion criteria, a focus on scalability in clinical settings, and a naturalistic design. Several issues warrant discussion, however.

First, our findings suggest the inclusion criteria (and hence the ideal population for this intervention) may have been too broad. In this study, nearly all (94.0%) of the follow-up suicide attempts were made by patients with preadmission suicide attempts. Because patients with prior suicide attempts have more complicated clinical profiles (eg, more severe symptoms and poorer problem-solving abilities),^23,29,30,31^ it is unsurprising that this subgroup had more follow-up suicide attempts. However, the near absence of follow-up suicide attempts among patients with no preadmission attempts was unexpected considering that they were admitted for psychiatric inpatient care presumably with acutely elevated suicide risk. The RCT was, therefore, adequately powered for patients with a prior suicide attempt but underpowered for patients with lower risk. The extreme rarity in this subgroup contributed to the low rate of follow-up suicide attempts in this trial compared with previous inpatient studies (11.7% vs 24.0% to 40.0%).^3,10,25,26,27,28^ It is possible that the control condition, which included safety planning (an evidence-based suicide prevention intervention^32^), was therapeutic on its own.

Second, recurrent suicide attempts were common, but the primary end point could not account for recurrence. The trial was, therefore, designed to examine change in the likelihood of making any vs no suicide attempts rather than change in the rate of repeated suicide attempts. Future studies of this diagnostic therapeutic intervention and other interventions initiated prior to discharge from a psychiatric inpatient unit could benefit from designs that account for these considerations.

Third, the application design and implementation need consideration. In this trial, patients were not required to use the application following discharge and did not meet with anyone who could support intervention adherence. This condition could explain why, on average, patients completed only 4.4 of 12 sessions. Although low in an absolute sense, application engagement is comparable to previously reported engagement rates for self-guided digital mental health interventions. A meta-analysis by Garrido et al,^33^ for instance, found that users frequently complete less than half of digital mental health components on their own. Engagement with the digital therapeutic application was also associated with reduced suicide attempt rates in this trial; on average, the suicide attempt rate among patients with prior attempts decreased by 14.0% for each digital therapeutic module completed, suggesting that greater engagement through formal integration of the application into ongoing mental health care with a trained clinician after discharge could potentially amplify its effects. This approach could promote shared responsibility for care transitions, thereby enhancing clinical outcomes. Reducing the number of sessions within the application might also improve engagement.

The findings should be considered within the context of multiple previous RCTs testing the efficacy of suicide-focused CBTs in reducing suicide attempts. In those trials, suicide-focused CBTs were superior for reducing suicidal ideation and reduced suicide attempts by 40% to 60% among patients with a history of suicide attempts and compared with TAU.^6,7,8,9,10^ Although we did not find a significant difference specific to the primary end point, the differences in secondary end points and 58.3% reduction in suicide attempt rate among patients with prior suicide attempts favored the digital therapeutic intervention, and these findings are consistent with results of prior studies of suicide-focused CBTs.

### Limitations

This RCT has several limitations. First, because the study was stopped early due to surpassing the futility boundary of the primary end point, the results were based on a smaller sample size and on less data than originally planned. Second, the treatment groups differed on a few variables at baseline despite randomization. To minimize this impact, we controlled statistically for these baseline differences. Third, our sample was recruited and enrolled during inpatient psychiatric unit stays. Results, therefore, may not generalize to patients who were not engaged while in a hospital setting. Fourth, because the intervention required ownership of a smartphone, some patients with low socioeconomic status could not participate. Finally, as most previous studies investigating suicide prevention interventions, this trial did not have sufficient power to detect differences in suicide deaths. Although the long follow-up period (2 years) increased the likelihood of capturing suicide deaths, larger samples are needed to investigate the potential effect of the digital therapeutic intervention and other suicide prevention treatments on suicide mortality.

## Conclusions

In this multisite RCT of patients hospitalized with elevated suicide risk, the digital therapeutic application designed to deliver an empirically supported suicide prevention treatment had no effect on time to first actual suicide attempt but led to a sustained reduction in suicidal ideation. Nonprespecified analyses further showed that patients with prior suicide attempts who received the digital therapeutic intervention had a 58.3% relative rate reduction in recurrent suicide attempts and were more likely to be rated as clinically improved.

## Notes

Supplement 1.
Trial Protocol

Supplement 2. **eMethods.** Description of OTX-202, A Prescription Digital Therapeutic **eTable 1.** Site Characteristics **eTable 2.** Percentage of Patients in Each Treatment Group Who Completed Each Module **eTable 3.** Adverse Events (AEs) Reported by Enrolled Participants, Full Sample (n=331) **eTable 4.** Adverse Events (AEs) Reported by Participants Assigned to OTX-202 (n=164) **eTable 5.** Adverse Events (AEs) Reported by Participants Assigned to Control (n=167)

Supplement 3.
Data Sharing Statement
