# Supplementary material for: A Digital Therapeutic Intervention for Inpatients With Elevated Suicide Risk: A Randomized Clinical Trial
Source: JAMA Netw Open. 2025 Aug 8;8(8):e2525809. doi: 10.1001/jamanetworkopen.2025.25809 (PMC12334960; doi:10.1001/jamanetworkopen.2025.25809)
Supplement: Supplement 1. — Trial Protocol [file jamanetwopen-e2525809-s001.pdf]

**TITLE:**	A Randomized, Double Blind, Controlled Study to Evaluate the Safety and Effectiveness of a Digital Therapeutic for Adults with a History of Suicidal Ideation and/or Suicide Attempts
**Date**:	November 24, 2021
**Sponsor**:	Oui Therapeutics, LLC<br>4 Science Park<br>New Haven, CT 06511
**Protocol Number**:	2020-Oui-001<br>Version Number: 1.

CONFIDENTIALITY STATEMENT
The confidential information in this document is provided to you as an Investigator or consultant for review by you, your staff, and the applicable Institutional Review Board (IRB) or Ethics Committee (EC). Your acceptance of this document constitutes agreement that you will not disclose the information contained herein to others without written authorization from Oui Therapeutics, LLC.

TABLE OF CONTENTS

STATEMENT OF COMPLIANCE 5
INVESTIGATOR'S SIGNATURE 7
1. PROTOCOL SUMMARY 8
1.2 ABBREVIATIONS 12
1.3 STUDY SCHEMA 14
1.4 SCHEDULE OF ACTIVITIES 15
2. INTRODUCTION 17
2.1 BACKGROUND 17
2.2 AVIVA 18
2.3 RISK/BENEFIT ASSESSMENT 19
3. OBJECTIVES AND HYPOTHESES 21
3.1 PRIMARY OBJECTIVES AND HYPOTHESES 21
3.2 SECONDARY OBJECTIVES AND HYPOTHESES 21
4. STUDY DESIGN 22
4.1 OVERALL DESIGN 22
4.2 DESCRIPTION OF THE TWO TREATMENT CONDITIONS 22
4.3 DEFINITION OF END OF TRIAL 23
5. STUDY POPULATION 23
5.1 PARTICIPANT INCLUSION CRITERIA 23
5.2 PARTICIPANT EXCLUSION CRITERIA 24
6. PARTICIPANT RECRUITMENT 24
7. STUDY ASSESSMENTS AND PROCEDURES 24
7.1 ASSESSMENTS 24
7.2. INFORMED CONSENT 29
7.3 SCREENING AND ELIGIBILITY ASSESSMENT 29
7.4 PARTICIPANT ENROLLMENT 29
7.5 RANDOMIZATION CRITERIA 30
7.6 CONCOMITANT MEDICATIONS/PSYCHOTHERAPY 30
7.7 RETENTION METHODS 30
7.8 STUDY INTERVENTION COMPLIANCE 31
8. STUDY DISCONTINUATION AND PARTICIPANT DISCONTINUATION/WITHDRAWAL 31
8.1 DISCONTINUATION OF STUDY SITE OR STUDY 31

8.2 PARTICIPANT DISCONTINUATION/WITHDRAWAL FROM THE STUDY ... 31
9. SAFETY ... 32
9.1 SAFETY HYPOTHESIS ... 32
9.2 ADVERSE EVENTS ... 32
9.3 RATIONALE FOR IDENTIFICATION OF ADVERSE CLINICAL EVENTS ... 33
9.4 EXPECTED ADVERSE EVENTS IN PARTICIPANTS ... 33
9.5 SERIOUS ADVERSE EVENTS ... 33
9.6 UNANTICIPATED ADVERSE DEVICE EFFECTS ... 33
9.7 REPORTING OF ADVERSE EVENTS ... 34
9.8 REPORTING PERIOD ... 34
9.9 SAFETY MONITORING AND REPORTING ... 34
10. CLINICIAN TRAINING AND SUPERVISION ... 35
11. STATISTICAL CONSIDERATIONS ... 36
11.1 STATISTICAL PROCEDURES AND DATA ANALYSIS ... 36
11.2 MINIMUM CLINICALLY IMPORTANT DIFFERENCE (MCID) ... 38
11.3 ANALYSIS SETS, SENSITIVITY ANALYSES AND SUBGROUP ANALYSIS ... 39
11.4 ADDITIONAL CONSIDERATIONS ... 40
11.5 STOPPING RULES ... 42
12. APPROACH TO ENSURE INTEGRITY OF DATA TO SUPPORT PRIMARY ENDPOINTS 42
12.1 REDUCE LIKELIHOOD OF PLACEBO EFFECT ... 42
12.2 REDUCE LIKELIHOOD OF SELECTION BIAS IN RECRUITMENT AND ENROLLMENT ... 43
12.3 REDUCE LIKELIHOOD OF INACCURATE OR BIASED PATIENT REPORTS DURING ASSESSMENTS ... 43
12.4 REDUCE LIKELIHOOD OF STAFF DEVIATING FROM THE DATA COLLECTION PROTOCOL ... 43
13. DATA SECURITY FOR AVIVA ... 43
13.1 CYBERSECURITY AND DATA COLLECTION ... 43
13.2 SECURITY CULTURE ... 44
13.3 COMMUNICATION ... 44
13.4 ROLE-BASED PRODUCTION ACCESS ... 44
13.5 DATA ENCRYPTION ... 44
13.6 SECURITY CULTURE ... 44
13.7 TESTING ... 44

14. DIRECT ACCESS TO SOURCE DATA/DOCUMENTS 44
15. QUALITY CONTROL AND QUALITY ASSURANCE PROCEDURES 44
16. ETHICS 44
16.1 DECLARATION OF HELSINKI 44
16.2 ICH GUIDELINES FOR GOOD CLINICAL PRACTICE 44
16.3 APPROVALS 45
16.4 PARTICIPANT CONFIDENTIALITY 45
16.5 OTHER ETHICAL CONSIDERATIONS 45
17. DATA HANDLING AND RECORD KEEPING 45
18. FINANCING AND INSURANCE 45
19. PUBLICATION POLICY 45
20. REFERENCES 46
ATTACHMENT A 50
ATTACHMENT B 51
ATTACHMENT C 55
ATTACHMENT D 58

STATEMENT OF COMPLIANCE

This trial will be conducted in compliance with the protocol, International Council on Harmonization Good Clinical Practice (ICH GCP) and applicable state, local and federal regulatory requirements. Each investigational site must provide this protocol and the associated informed consent documents and recruitment materials for review and approval by an appropriate Institutional Review Board (IRB) or Ethics Committee (EC). Any amendments to the protocol or consent materials must also be approved before implementation.

Sponsor Protocol Approval Page

STUDY TITLE:	A Randomized, Double Blind Controlled Study to Evaluate the Safety and Effectiveness of a Digital Therapeutic for Adult Patients with Suicidal Ideation and/or Suicide Attempts
PROTOCOL NUMBER:	2020-Oui-001
VERSION NUMBER:	1.2

We, the undersigned, have read and approve the protocol specified above and agree on its content.

Patricia Simon<br>VP, Clinical	
	Date:<br>11/30/2021
Brian Keenaghan<br>VP, Operations	
	Date:<br>11/30/2021

INVESTIGATOR'S SIGNATURE

The signature below constitutes the approval of this protocol and provides the necessary assurances that this study will be conducted according to all stipulations of the protocol, including all statements regarding confidentiality, and according to local legal and regulatory requirements and applicable US federal regulations and ICH guidelines, as described in the Statement of Compliance above.

PI or Clinical Site Investigator:

Signed:		Date:	
Name:			
Title:			

Investigator Contact Information

Affiliation:

Address:

Telephone:

Email:

1. PROTOCOL SUMMARY

1.1 SYNOPSIS

Title	A Randomized, Double Blind Controlled Study to Evaluate the Safety and Effectiveness of a Digital Therapeutic for Adult Patients with Suicidal Ideation and/or Suicide Attempts
Sponsor	Oui Therapeutics, LLC
Funding Organization	Oui Therapeutics, LLC & National Institute of Health (NIH)
Number of Sites	Up to 8
Rationale	Suicide is a top ten cause of death in the US and rates of suicide have increased steadily for the last twenty years (CDC, 2020). Despite this, there is a paucity of research on treatment programs designed to reduce suicide attempts and there are no FDA approved or cleared digital therapeutics. The purpose of this study is to evaluate the effectiveness of Aviva™ (a digitally delivered cognitive- behavioral intervention integrated with a messaging program) + treatment as usual (TAU) compared to a sham app + TAU in reducing suicide attempts).
Study Design	This double blind, randomized controlled trial will evaluate the effectiveness of Aviva + TAU versus a sham + TAU among 391 participants, adult inpatients, who had recent suicidal ideation with intent or suicide attempts. Study participation will start when participants are ready for discharge. Participants and research assessors will be blinded to treatment assignment. The primary endpoint is time to first suicide attempt, analyzed as a time-to-event variable. The primary endpoint will be analyzed in a group sequential design with Looks at 60%, 75%, 90% and 100% of the expected number of suicide attempts. Overall Type I error (alpha) will be less than 0.025 one-sided.
Primary Objective	The primary objective of this study is to assess the effectiveness of a digital intervention (Aviva) in reducing suicide attempts in patients with a recent history of suicidal ideation or attempted suicide. The time to first suicide attempt after randomization is the primary endpoint. Time to event analysis will occur when the expected number of events occur.
Secondary Objectives	To evaluate the effectiveness of Aviva + TAU (vs. a sham app + TAU) on suicide ideation (at 24 weeks).<br><br>To explore whether changes in decision-making and emotion regulation are mechanisms of action for Aviva.
Safety Objectives	To evaluate the safety of Aviva + TAU (vs. a sham app + TAU).
Number of Participants	391

Subject Selection Criteria	Inclusion Criteria:<br>1. Ages 22 to 70 years<br>2. Patients recently hospitalized because of attempted suicide or because of suicide ideation with a total score of 5 or higher on the Scale for Suicide Ideation (SSI)) and intent to harm themselves. Hospitalized is defined as admission to a medical or psychiatric service for further assessment and care including observation units, intensive care or other medical units and psychiatric units.<br>3. Owns a smartphone capable of downloading and running apps.<br>4. Willing and able to complete enrollment procedures and allow for review of medical records for two years to look for suicide attempts<br>5. Able to understand the nature of the study and provide written informed consent<br>6. Able and willing to provide at least two verifiable contacts for emergency purposes (verification of emergency contacts occurs within 24 hours of all study visits and as needed at the discretion of the Principal Investigator (PI))<br><br>Exclusion Criteria:<br>1. Patients who have untreated psychosis or are treated but still have active psychosis<br>2. Patients who upon examination appear to be impaired by the use of alcohol or other substance(s) at the time of evaluation for inclusion in the study.<br>3. Patients who sign, or have signed, an informed consent form to participate in any clinical research, trial, investigation, study, or activity that is or will be ongoing while the patient is a participant in this study, except as authorized by the Principal Investigator.<br>4. Patients who upon clinical examination are cognitively impaired or whose cognitive performance may adversely impact the integrity of the data.<br>5. Patients with a medical condition that, in the opinion of the PI, may compromise, interfere, limit, effect or reduce the subject's ability to complete a study of 104 weeks duration or may adversely impact the safety of the subject or the integrity of the data. Examples of considerations include terminal illness, psychosis, severe or unstable medical condition.
Investigational Device / Intended Use	Aviva™ is a prescription digital therapeutic (PDTx) software as medical device (SaMD). Aviva™ is intended to reduce the risk of suicide in adults 22 years or older who recently attempted suicide or had suicidal ideation with intent to self-harm, and who are receiving treatment as usual (TAU) from a mental health prescribing provider and/or at an outpatient behavioral health (BH) setting. BH settings include clinics, mental health centers, day treatment or partial hospital programs, and group private practices (and as feasible, the elements of standard care are recommended for solo private practice therapists). Aviva™ is intended

	for adjunctive use with treatment-as-usual (TAU) in the outpatient care environment.
Control	Sham App + TAU
Duration of Subject Participation and Duration of Study	Participants will be on study for up to 104 weeks (2 years)<br>● App testing: 12 weeks<br>● Follow-up: 92 additional weeks
Effectiveness Evaluations	Suicide Attempts: Suicide Attempt Self-Injury Interview (SASII), medical record review<br>Suicide Ideation: Scale for Suicide Ideation (SSI)<br>Depression Severity: Beck Depression Inventory-II (BDI-II)<br>Hopelessness Severity: Beck Hopelessness Scale (BHS)<br>Decision Making: Monetary Choice Questionnaire (MCQ)<br>Emotion Regulation: Cognitive Emotion Regulation Questionnaire (CERQ)
Primary Endpoint	The primary endpoint for this study will be suicide attempts (time to event) by 104 weeks. Suicide attempts will be assessed using SASII and medical record review.
Secondary Endpoints	The secondary endpoint for this study will be suicide ideation (at 24 weeks).<br><br>Exploratory analyses will assess treatment effect trends using additional assessments of suicide ideation, depression, and hopelessness from weeks 0 to 104.
Other Evaluations	Clinical Impression:<br>Clinical Global Impressions Scale - severity subscale (CGI-S)<br>Clinical Global Impressions Scale - change subscale (CGI-C)
Safety Evaluations	Any adverse events or negative effects discovered during the study will be reported following standard procedures. Assessments will be carried out throughout the study to help participants in need of more extensive support to know when to contact their TAU provider and be guided to crisis contact services. Communication within the digital therapeutic will be monitored to identify participants at risk of harm to self or signaling a need for more extensive support. Potential ambiguities regarding standard safety procedures, types and numbers of measures undertaken to assure participant safety and types and numbers of unforeseen safety issues will be reported.
Statistics Primary Analysis Plan	Survival analyses will be conducted using the log-rank test for the effectiveness of the intervention on the time to the first suicide attempt after randomization while controlling for censoring effects due to the differential length of follow-up or the completion of follow-up without a suicide attempt. Time to suicide attempt will be measured by calculating the total number of days from enrollment to the first suicide attempt. For participants without a suicide attempt, the total number of days since enrollment to the last assessment will be calculated. The study will test alpha at the .025 significance-level (one-sided). This study will use a group sequential design. The group sequential design has 3 interim sequential tests (4 total looks including final analysis). The looks will occur when 60%, 75%, 90% and 100% of the expected events (i.e.,

	suicide attempts) have occurred. The O'Brien-Fleming spending function is used to determine the effectiveness test boundary. A binding futility boundary is incorporated in this design, also with an O'Brien-Fleming boundary.
Rationale for Number of Participants	A total sample size of 391 (with 52 events) is required to achieve 90% power to detect a hazard ratio of 0.3826 (for survival rates of 0.9240 in the Aviva group [group 1] and 0.8124 in sham app group [group 2] at 18 months), using a one-sided log rank test with 2.5% significance level (i.e., p = 0.025) assuming that the survival rates are exponential and that the total study time is 104 weeks (24 months) with 52 weeks (12 months) accrual. These results assume that the group sequential design has 3 interim sequential tests (4 total looks including final analysis). The O'Brien-Fleming spending function is used to determine the effectiveness test boundary. The study is designed with the expectation that participants will be followed for 104 weeks, but time to event analysis will occur when the expected number of events occur.

1.2 ABBREVIATIONS

ACE	Adverse Childhood Experiences Questionnaire
AE	Adverse Event
BCBT	Brief Cognitive Behavioral Therapy to Prevent Suicide Attempts
BDI-II	Beck Depression Inventory, 2nd Edition
BHS	Beck Hopelessness Scale
CBT	Cognitive Behavioral Therapy
CBT-SP	Cognitive Behavioral Therapy for Suicide Prevention
CDC	Center for Disease Control
CERQ	Cognitive Emotion Regulation Questionnaire
CEQ	Credibility/ Expectancy Questionnaire
CGI-C	Clinical Global Impression Change Subscale
CGI-S	Clinical Global Impression Severity Subscale
CRO	Clinical Research Organization
C-SSRS	Columbia-Suicide Severity Rating Scale
CTA	Clinical Trial Agreement
CT-SP	Cognitive Therapy for Suicide Prevention
DSMB	Data Safety Monitoring Board
FDA	Food and Drug Administration
GCP	Good Clinical Practice
GP	General Practitioner
HIPAA	Health Insurance Portability and Accountability Act
ICH	International Council for Harmonisation of Technical Requirements for Pharmaceuticals for Human Use
IRB	Internal Review Board

ISI	Insomnia Severity Index
ITT	Intent-to-Treat
GCP	Good Clinical Practice
GP	General Practitioner
LEC	Life Events Checklist
MCID	Minimum Clinically Important Difference
MCQ	Monetary Choice Questionnaire
PI	Principal Investigator
PIH	Protected Health Information
RCT	Randomized Clinical Trial
REML	Restricted Maximum Likelihood
SA	Suicide Attempt
SAE	Serious Adverse Events
SI	Suicide Ideation
SSI	Scale for Suicide Ideation
SASII	Suicide Attempt Self-Injury Interview
SCS	Suicide Cognitions Scale
SOPs	Standard Operating Procedures
SUS	System Usability Scale
TAU	Treatment as Usual
UADE	Unanticipated Adverse Device Effects

1.3 STUDY SCHEMA

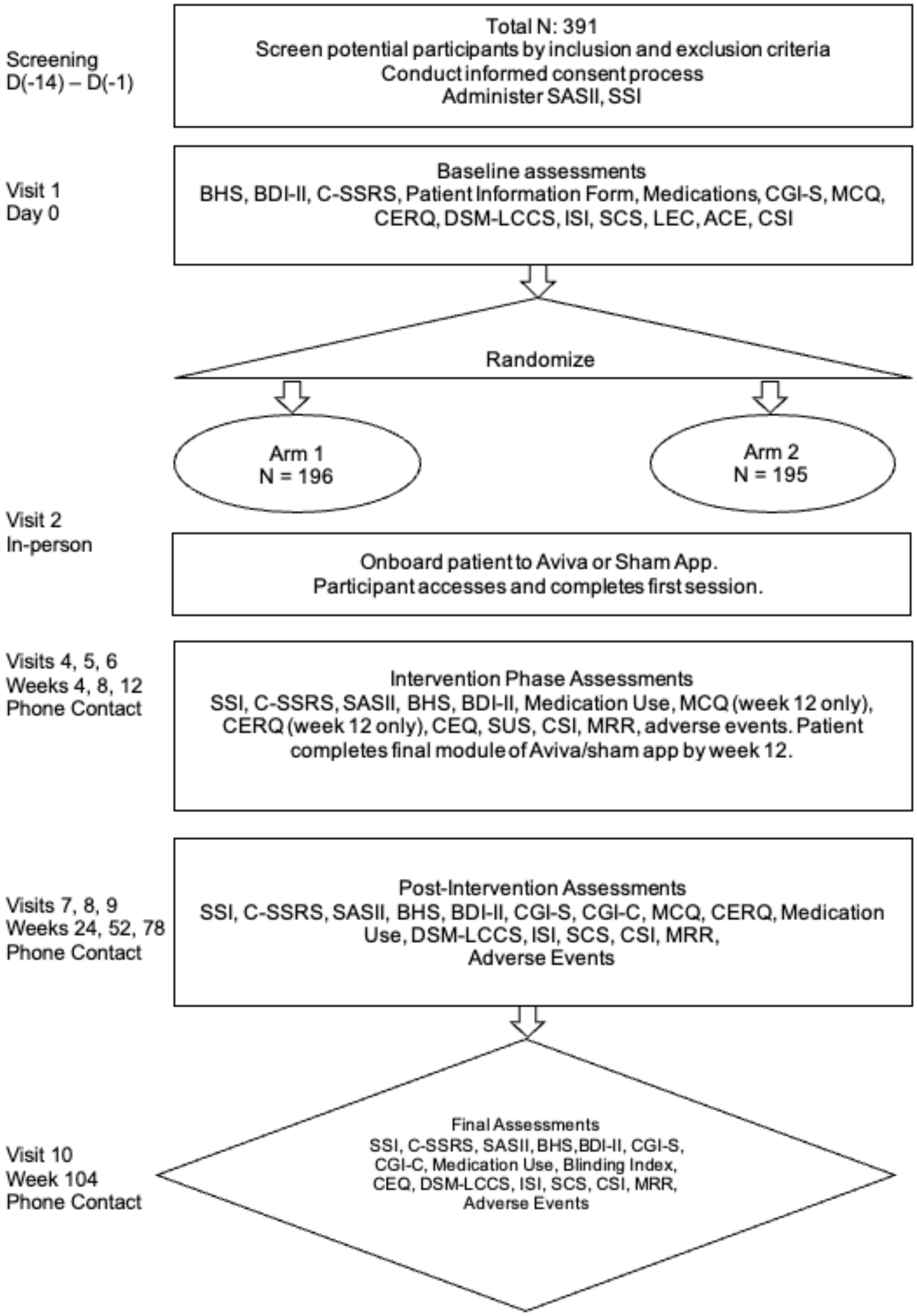

1.4 SCHEDULE OF ACTIVITIES

Table 1. Schedule of Assessments

Study Assessment	Screen Period[a]	Baseline		App Testing Period				Follow-up Period			
**Visit***	**Screening**	**1**	**2**	**3**	**4**	**5**	**6**	**7**	**8**	**9**	**10**
**Timepoint Days (D) / Weeks (W)**	**D (-14) - (-1)**	**D0**	**D1**	**W1**	**W4**	**W8**	**W12**	**W24**	**W52**	**W78**	**W104**
In Person / Phone Call	*In Person*	*In Person*	*In Person*	*Phone Call*	*Phone Call*	*Phone Call*	*Phone Call*	*Phone Call*	*Phone Call*	*Phone Call*	*Phone Call*
Visit Window				*+/- 3 days*	*+/- 3 days*	*+/- 3 days*	*+/- 3 days*	*+/- 3 days*	*+/- 3 days*	*+/- 3 days*	*+/- 3 days*
**Informed Consent**	X										
**Participant Information Form[b]** *(including contact information, demographics and medical history)*		X									
**Randomization**			X								
**Enrollment in Aviva** ***(Onboarding)***			X[c]								
**Current and past psychiatric diagnoses**	X										
**Suicide Attempt Self-Injury Interview (SASII)** [d]	X				X	X	X	X	X	X	X
**Scale for Suicide Ideation (SSI)**[d]	X				X	X	X	X	X	X	X
**Beck Hopelessness Scale (BHS)**		X			X	X	X	X	X	X	X
**Beck Depression Inventory (BDI-II)**		X			X	X	X	X	X	X	X
**Columbia-Suicide Severity Rating Scale (C-SSRS)** [d]		X			X	X	X	X	X	X	X
**Clinical Global Impressions (CGI-S) Scale -severity subscale**		X						X	X	X	X
**Clinical Global Impressions (CGI-C) Scale - change subscale**								X	X	X	X
**Monetary Choice Questionnaire (MCQ)**		X					X	X	X	X	
**Cognitive Emotion Regulation Questionnaire (CERQ)**		X					X	X	X	X	
**Blinding Index**											X
**Credibility/ Expectancy Questionnaire (CEQ)**							X				X
**System Usability Scale (SUS)**					X	X	X				

Study Assessment	Screen Period[a]	Baseline		App Testing Period				Follow-up Period			
**Visit***	**Screening**	**1**	**2**	**3**	**4**	**5**	**6**	**7**	**8**	**9**	**10**
**Timepoint Days (D) / Weeks (W)**	**D (-14) - (-1)**	**D0**	**D1**	**W1**	**W4**	**W8**	**W12**	**W24**	**W52**	**W78**	**W104**
In Person / Phone Call	*In Person*	*In Person*	*In Person*	*Phone Call*	*Phone Call*	*Phone Call*	*Phone Call*	*Phone Call*	*Phone Call*	*Phone Call*	*Phone Call*
Visit Window				*+/- 3 days*	*+/- 3 days*	*+/- 3 days*	*+/- 3 days*	*+/- 3 days*	*+/- 3 days*	*+/- 3 days*	*+/- 3 days*
**Diagnostic and Statistical Manual of Mental Disorders** *Fifth Edition (DSM-5) Level 1 Cross-Cutting Symptom Measure (DSM-LCCS)*		X						X	X	X	X
**Insomnia Severity Index (ISI)**		X						X	X	X	X
**Suicide Cognitions Scale (SCS)**		X						X	X	X	X
**Life Events Checklist (LEC)**		X									
**Adverse Childhood Experiences Questionnaire (ACE)**		X									
**Cornell Service Index (CSI)** *Assessment of healthcare resource utilization*		X			X	X	X	X	X	X	X
**Site Check- in: Compliance with Discharge Instructions**				X							
**Medical Record Review (MRR)**				X	X	X	X	X	X	X	X
**Frequency of Use of App Tracked by Software[e]**				X	X	X	X	X	X	X	X
**Adverse Events**	X	X	X	X	X	X	X	X	X	X	X
**Medication Use**		X			X	X	X	X	X	X	X

*Visit refers to the follow up contact via telephone between Principal Investigator (PI) or designee and the study participant
a: Screening will be conducted while participants are in an inpatient psychiatric facility
b: Form to collect demographic, medical history and contact information
c: App is downloaded before the end of the study visit
d: Assessments/scales/interviews administered by research team on a phone call with patient
e: Logins since the last assessment period will be recorded in the source documents & eCRF

2. INTRODUCTION

Suicide is one of the top 10 causes of death in the US (CDC, 2020). In 2018 alone more than 48,000 people died by suicide and nearly 500,000 people stayed at a hospital overnight (or longer) due to a suicide attempt (CDC, 2020). A related phenomenon, suicidal ideation (i.e., thoughts about self-harm, with deliberate consideration or planning of possible techniques for causing one's own death) impacts approximately 4.3% of adults over age 18 (SAMHSA, 2017) and causes significant distress (Bryan, et al., 2013). While Clozaril was developed to prevent suicide among those who also have Schizoaffective Disorder or Schizophrenia, to date, there are no FDA cleared or approved products for those who do not have a diagnosis of Schizoaffective Disorder or Schizophrenia. To address this gap, Oui Therapeutics has developed Aviva, a digital therapeutic that is based on CBT procedures that were designed for suicide prevention (i.e., suicide-specific CBT). The scientific premise for developing Aviva is drawn from research on non-digital versions of suicide-specific CBT that were implemented in a face-to-face setting (Brown et al., 2005; Rudd et al., 2015). This research showed that suicide-specific CBT significantly decreased suicide attempts and suicidal ideation (Rudd et al., 2015). Despite the demonstrated significant effects, face-to-face, suicide-specific CBT is not widely implemented because of logistical challenges (e.g., limited number of trained providers). Therefore, there is a significant need for effective, scalable suicide prevention treatments in the form of digital therapeutics. As a result, the developers of the original suicide-specific protocols have worked with Oui Therapeutics to develop Aviva.

2.1 BACKGROUND

2.1.1 Summary of Clinical Findings for Suicide Specific Cognitive Behavioral Therapy

Two suicide-specific Cognitive Behavioral Therapy (CBT) interventions have been proven to be efficacious in helping prevent re-attempts at suicide: Brief Cognitive Behavioral Therapy to Prevent Suicide Attempts (BCBT) and Cognitive Therapy for Suicide Prevention (CT-SP). These related CBT interventions are specialized protocols that are specific to suicide. They are based on the theory that through activation of a “suicide mode,” thoughts, beliefs and expectations (shaped by previous life experiences), combined with deficiencies in self-regulation and coping, can contribute to suicidal thoughts and/or suicide attempts (i.e. suicidal behaviors; Rudd, 2000). In these treatments, suicidal individuals are taught: 1) to identify personal indicators of emerging emotional crises, 2) to employ self-regulatory strategies that reduce or distract from emotional distress, and 3) to adopt more flexible thoughts, beliefs, and expectations that undermine suicidal behaviors. Critically, each patient is assisted in identifying their unique specific set of circumstances and configurations of suicidal thoughts that can lead to suicidal behavior. Structurally, both BCBT and CT-SP include an early phase that entails assessment and developing a plan for keeping participants safe (phase 1), an intermediate phase that involves teaching cognitive behavioral skills (phase 2) and a final relapse prevention phase that summarizes the skills participants learned. The treatments are delivered over 10 to 12 sessions.

There is strong empirical support for CT-SP and BCBT across clinical settings. Evidence to support early forms of face-to-face, suicide-specific CBT protocols first emerged in 2001 (Rudd et al., 2001). Evidence to support the comprehensive suicide prevention protocol was published in 2005 in the Journal of the American Medical Association. Brown et al. (2005) conducted a randomized clinical trial (RCT) testing CT-SP compared to treatment as usual (TAU) + tracking and referral in a sample of adults (N = 120) who attempted suicide and were evaluated at a hospital emergency department within 48 hours of the attempt. Compared to TAU, participants receiving the therapy were 50% less likely to make a suicide attempt during the 18-month follow-up period. In another study, Rudd et al. (2015) conducted an RCT among participants who either

attempted suicide or experienced suicidal ideation with intent. Participants were randomly assigned to BCBT + TAU (N=76) or TAU (N=76). From baseline to the 24-month follow-up, participants in BCBT were approximately 60% less likely to make a suicide attempt during that period than participants in TAU. Moreover, another study implementing a variation of these suicide-specific protocols showed a significantly faster decline in suicidal ideation as measured by the Scale for Suicide Ideation (Bryan et al., 2017). Two studies implementing similar protocols found support for sending short, standardized written encouragement notes at predetermined intervals (Comtois et al., 2019; Gysin-Maillart, et al., 2016).

In light of these collective findings, the procedures, mentioned above, have been recommended for use in the standard care of suicidal patients by the National Alliance for Suicide Prevention (2018) and the Joint Commission (2018, https://www.jointcommission.org/resources/patient-safety-topics/suicide-prevention). The procedures are also recommended in recent guidelines published by the VA and DO (2019,https://www.healthquality.va.gov/guidelines/MH/srb/VADoDSuicideRiskFullCPGFinal5088212019.pdf). Despite the demonstrated effectiveness of the BCBT and CT-SP interventions and national calls for their widespread adoption, neither are integrated into standard care for suicidal patients (Ghahramanlou-Holloway et al., 2015). While the expectation of national accreditation bodies such as the Joint Commission is that these interventions would be the standard of care, there are challenges to implementation. First, to deliver these interventions face-to-face, substantial training and ongoing supervision is required and is not feasible. Second, these interventions require a substantial time commitment from patients; sessions last approximately 60 minutes and often necessitate a patient taking time off from work in addition to travel time. Third, there is a shortage of appropriately trained therapists, and attempts to train more therapists have not been successful. As a result, the creators of both protocols have worked to develop Aviva, a digital therapeutic, to scale the availability of these protocols. The developers joined Oui Therapeutics to exclusively develop Aviva. Thus, there is no other known app that digitally delivers the only suicide prevention therapy protocols proven in clinical trials to reduce suicide attempts by 50% or more.

2.1.2 Overall Rationale for the Study

The purpose of this study is to conduct a double-blind randomized controlled trial to evaluate the safety and effectiveness of Aviva + TAU compared to a sham app + TAU in reducing suicide attempts (primary endpoint) at 104 weeks (two years). Aviva is a 12-week digital intervention intended to help reduce suicide attempts by teaching participants to: 1) identify personal indicators of emerging emotional crises, 2) employ self-regulatory strategies that reduce or distract from emotional distress, and 3) adopt more flexible thoughts, beliefs, and expectations that undermine suicidal thoughts and suicide attempts. This study will serve as a pivotal effectiveness and safety study in support of regulatory agency requirements.

2.2 AVIVA

2.2.1 App Overview

Aviva is designed to be user friendly and for use by patients that does not require guidance from a qualified health professional after the initial onboarding session. Patient progression through the 12 sessions of the Aviva treatment platform is divided into phases. Consistent with the procedures presented in the BCBT and CT-SP protocols, Aviva includes an early phase that entails assessment and development of a plan for keeping participants safe (phase 1), an intermediate phase that involves teaching suicide specific cognitive and behavioral skills (phase 2) and a final phase that summarizes specific skills learned to prevent relapse of suicidal thinking and suicide attempts.

The three phases are executed via 12 sessions (see Table 2). Participants complete an initial onboarding session that includes planning for safety and identification of warning signs from the most recent suicide attempt. After completion of the initial onboarding session, participants complete several skills training sessions, each designed to take 10-20 minutes to complete. For a description of the rationale for the sessions, see the *Overview of the Product Development* section. Note that the design and development of Aviva is ongoing.

Table 2. Intervention phases from proven cognitive behavioral protocols (BCBT/CT-SP) that are included in Aviva

Phase	BCBT (Rudd et al., 2015)	CT-SP (Brown et al., 2005)	Aviva
Phase 1 (Planning for Safety)	X	X	X
Phase 2 (Cognitive Behavioral Skills Training)	X	X	X
Phase 3 (Relapse Prevention)	X	X	X

Table 3. Aviva Sessions and Duration

Session Topic	App Time (min.)
Onboarding	20 – 45
Reasons for Living/Hope kit	10 – 20
Get Going (Activity Planning/Behavior Activation)	10 – 20
Relaxation and Mindfulness	10 – 20
Improve Sleep (Sleep tracking)	10 – 20
Spot it (ABC Exercise)	10 – 20
Test it (Challenging Questions)	10 – 20
Switch it (Patterns of Problematic Thinking)	10 – 20
Coping Cards	10 – 20
Review Phase 1 (Relapse Prevention)	10 – 20
Review Phase 2 (Relapse Prevention)	10 – 20
What's Next? (Termination Planning)	10 – 20

For an overview of the development process for AVIVA, refer to appendix B.

2.3 RISK/BENEFIT ASSESSMENT

2.3.1 Known Potential Risks

Potential participants will be invited to volunteer for the study if they have been voluntarily admitted to an inpatient psychiatric unit because of a suicide attempt or have clinically significant suicidal ideation and intent to harm themselves. Aviva does not involve any invasive or risky medical procedure. Aviva can be considered no greater risk than ordinarily encountered in daily life, however it will be used by individuals who have reported suicidal ideation, so there is high risk for adverse events.

Given that Aviva delivers digitized suicide prevention, there is risk that patients may experience worsened condition, delayed access/progress in the adjunct treatment or a patient data privacy breach. These risks, associated hazardous situations and mitigations are presented below.

Worsened Condition: This risk of this harm may emerge if the program is unproven, the method used does not implement a proven method effectively enough to stabilize or improve patient condition, the instructions for use are incorrect/misleading, and/or the patient uses Aviva as a standalone treatment.

We have mitigated the risk of situations that may give rise to worsened condition. Aviva is based on Rudd et al 2015 and Brown et al 2005. These protocols have been proven effective in reducing suicide attempts. Additionally, the developers of suicide-specific CBT (including M. David Rudd, Craig Bryan, and Gregory K. Brown) have reviewed the protocol to ensure adherence to the method published in Rudd et al 2015 and Brown et al 2005. We have developed detailed instructions for use that clearly guides the user on how to use Aviva and it has been reviewed by clinical experts. Furthermore, the Aviva Chatbot guides the user step-by-step to minimize the risk of error. We have reduced the risk that patients would use Aviva as a standalone treatment by making it available by prescription only and for use under the guidance of the physician. Although it is used under the guidance of a physician, Aviva therapy is patient-driven and there will not be, by design, continuous monitoring of patient progress by the treating clinician. Aviva's instructions for use and labeling clearly outline that Aviva is an adjunct treatment and must not be used as a standalone treatment or replacement to clinic or outpatient treatment.

Access to Adjunct Treatment Not Progressing or Delayed: The risk of this harm may emerge if there is registration/setup failure, incorrect, insufficient or unclear instructions for use, Aviva is unavailable or not delivering therapy, Aviva is not assisting the patient with progressing through or continuing therapy.

We have mitigated risk of treatment not progressing or being delayed. A clinician will be available during the registration process to help guide patients through the registration/setup process. Should the problem persist, patients should contact support@ouitherapeutics.com with any technical questions or concerns. Aviva's instructions for use have been informed by feedback from users who participated in the Aviva feasibility study. Furthermore, this document has been revised by experts in the field and the Aviva Chatbot guides the user throughout Aviva to reduce the risk of error.

Patient Data Privacy Breached: The risk of this harm may emerge due to the fact that this is delivered through mobile device access, due to patient actions or inactions, and/or due to physical security breach at a production facility.

We have mitigated the risk of patient data privacy breach. Any connection to the Aviva database requires a username and password. This password is known only to the end user (i.e., patient) and is not available to Oui personnel, and is thus sufficient proof of the patient's identity. Aviva directs the patient in creating a secured password after they enter their claim code. If the patient forgets to lock their phone there is a time lockout for Aviva after three minutes of being idle (i.e., the app is open on the patient's smartphone but not being used). Consistent with HIPAA compliance requirements, Oui Therapeutics has a policy to create, administer and oversee policies and procedures that ensure the prevention, detection, containment and correction of security violations that occur at Oui Therapeutics' work locations.

2.3.2 Known Potential Benefits

Aviva has digitized procedures and content from proven cognitive-behavioral protocols (Brown et al., 2005, Rudd et al., 2015, Bryan et al. 2017) to allow scalability and access to effective suicide prevention (per Joint Commission, Department of Veteran Affairs, and Department of Defense guidelines).

Aviva is intended to improve patients' adherence to CBT treatment for suicide prevention and thus lower frequency of suicide attempts. A digital approach will provide resources and evidence-based support systems to individuals who otherwise might go without them.

2.3.3 Assessment of Potential Risks and Benefits

Aviva, as a prescription digital therapeutic, is intended for use under the supervision of a provider and can be self-administered once it is prescribed. Thus, patients do not require ongoing guidance from the provider to progress through app sessions. Under the standard of care used today, patients are discharged from the hospital with medication and treatment guidelines, including follow up appointments for talk therapy for related conditions [if any]. Aviva is an additional tool that allows a patient to access suicide specific behavioral therapy exercises as needed. It is not intended to replace the standard of care. Instead, Aviva serves as an adjunct to such care. Even under standard care, a patient is likely to face the same risks as posed by Aviva: non-compliance with behavioral therapy recommendations, non-engagement, lack of follow up with clinicians, and potential re-attempts.

3. OBJECTIVES AND HYPOTHESES

3.1 PRIMARY OBJECTIVES AND HYPOTHESES

The primary objective of this study is to assess the effectiveness of a digital intervention (Aviva) in reducing suicide attempts in patients with a recent history of suicidal ideation or attempted suicide. The time to first suicide attempt after randomization is the primary endpoint. Time to event analysis will occur when the expected number of events occur.

3.2 SECONDARY OBJECTIVES AND HYPOTHESES

3.2.1 First Secondary Objective

Once the primary effectiveness analysis for suicide attempts is significant at the overall level of 0.05 (two-sided; 0.025 one-sided), then all of the significance level of 0.05 will be available to test secondary endpoints. The first secondary objective is to assess the effect of Aviva + TAU compared to the sham app + TAU on suicide ideation at 24 weeks. Suicidal ideation will be assessed using the SSI (Beck & Steer, 1993). Analyses will examine change in total score on the SSI. The hypothesis for suicide ideation is that in adults who have experienced suicidal ideation with intent or attempted suicide, Aviva will improve the SSI score compared to the control condition at 24 weeks.

3.2.2 Additional Secondary Objectives

Since cognitive behavioral therapy for suicide prevention (CBT-SP) is hypothesized to target decision-making and self-regulation processes, OUI will use bias-corrected bootstrapped estimates to conduct exploratory analyses (specifically mediation analyses; Preacher and Hayes, 2014) examining Aviva's effects on SA and SI through participants' scores on the Monetary Choice Questionnaire (MCQ) and the Cognitive Emotion Regulation Questionnaire (CERQ), respectively. Exploratory analyses will assess treatment effect trends by obtaining additional assessments of suicide ideation, depression, and hopelessness from weeks 0 to 104. Depression will be assessed using the BDI-II (Beck, Steer & Brown, 1996). Analyses will examine change in total score on the BDI-II. Hopelessness will be assessed using the BHS (Beck & Steer, 1988; Beck, Weissman, Lester, & Trexler, 1974).

3.2.3 Safety Objectives

Another objective is to assess the safety of Aviva. The safety hypothesis is that Aviva is safe for use by individuals with a previous history of suicidal ideation and attempts. Thus, no adverse events are anticipated specific to the Aviva device. Device specific adverse events are not anticipated because a) of the software nature of the treatment and b) participants in both conditions will receive treatment as usual. The primary risk to the participant is ineffective

treatment, resulting in a worsening of existing symptoms. This study will assess anticipated and unanticipated adverse events in the Aviva and sham conditions and assess for device relatedness. The primary anticipated events, therefore, include increased suicidal ideation and suicide attempts. Similar to the prior research, results from the SASII will be coded to match the C-SSRS (Comtois et al., 2019; FDA, 2012), which is consistent with FDA guidance for tracking suicide as an adverse event. For details on adverse events reporting in clinical trial see section 9.0.

4. STUDY DESIGN

4.1 OVERALL DESIGN

This double-blind RCT will evaluate the effectiveness of **Aviva + TAU versus a Sham + TAU** among 391 participants, adult inpatients, who had recent suicidal ideation with intent or suicide attempts. Study participation starts when consent is signed and patients are ready for discharge from an inpatient or emergency unit. Participants and assessors will be blinded to treatment assignment. The study is planned to take a total of 104 weeks from first patient randomized (52 weeks for enrollment, up to 104 weeks for patient follow up). An additional 4 months is planned for study close out. The primary endpoint will be analyzed in a group sequential design with looks at 60%, 75%, 90% and 100% of the expected number of suicide attempts. Overall Type I error (alpha) will be less than 0.025 one-sided.

4.2 DESCRIPTION OF THE TWO TREATMENT CONDITIONS

4.2.1 Sham App + Treatment as Usual

All patients enrolled will have scheduled follow up appointments consistent with standard of care guidelines, as part of TAU (NAASP, 2018). These will include appointments with psychiatrists and other mental health clinicians. Although TAU usually differs from site to site, the standard of care for patients who meet our inclusion criteria includes a suicide risk assessment, supportive listening, crisis resources, clinician assessment and referral to outpatient treatment. In addition to these TAU activities, a sham app will be used. The sham app will be a digitized version of the content and information included in standard of care (i.e., TAU). Specifically, the sham app will include the TAU information and materials that a participant normally would receive regarding the importance of compliance with treatment appointments, compliance with therapy, compliance with medications and how to follow up as needed. The sham app will also remind participants to check back with the content in the sham app. The sham app will be made to closely approximate the experience of the digital engagement of the Aviva treatment device arm. Towards this goal, the sham app activities will occur at the same frequency and duration as Aviva. The sham app will contain helpful information for the user. Participants' ability to determine whether they are in the active vs. control condition will be mitigated by the following:

- The sham app will be built on the same user interface as Aviva. For example, similar to Aviva, the sham app will include a chatbot, videos and reminder notifications.
- Similar to Aviva, the sham app will include 12 sessions.
- Sham app content will contain psychoeducational material, but will not include the suicide-specific cognitive behavioral skills training (i.e., active ingredients) included in Aviva.

Note, participants in the sham + TAU control group will not receive access to Aviva.

4.2.2 Aviva + Treatment as Usual

Participants in the Aviva condition will complete the procedures described under the description of "Aviva" Appendix B. At the time of preparation for discharge, participants will

complete the initial onboarding session with their clinician and then progress through sessions in the Aviva app on their own.

Table 4. Overlap of the components of Aviva +TAU and sham + TAU.

**Overlap of the TAU components in Aviva and Sham conditions.**			
**Component**	**Description**	**Sham + TAU**	**Aviva + TAU**
Suicide risk assessment	Structured interview regarding recent SI and lifetime history of SAs	X	X
Supportive listening	Unstructured conversation about recent stressors and current complaints	X	X
Cognitive behavioral skills training	Skills to teach individuals to observe personal behaviors and thoughts that may be problematic.		X
Plan for safety & crisis resources	Document warning signs, coping strategies, phone numbers of medical providers, phone numbers for other professional sources of help, and plans for making the environment safe.	X	X
Referral to treatment	Follow-up appointment with mental health care provider, and referral to other professional resources	X	X
Follow-up assessment	Research assessor contacts participant for interview	X	X

4.3 DEFINITION OF END OF TRIAL

The end of trial is the date of the last visit/ telephone follow up/ home visit of the last participant. A participant is considered to have completed the study if he or she has completed the baseline assessment, all intervention sessions, and all follow-up assessments.

5. STUDY POPULATION

Participants are those that have attempted suicide or have documented clinically significant suicide ideation (i.e., total score of 5 or higher on the SSI) and intent to harm themselves. Despite prior research indicating females are more likely to experience nonfatal suicide attempts and suicidal ideation than males, attempts will be made to recruit an equal number of men and women.

5.1 PARTICIPANT INCLUSION CRITERIA

1. Ages 22 to 70 years
2. Patients recently hospitalized because of attempted suicide or because of suicide ideation with a total score of 5 or higher on the Scale for Suicide Ideation (SSI)) and intent to harm themselves. Hospitalized is defined as admission to a medical or psychiatric service for further assessment and care including observation units, intensive care or other medical units and psychiatric units.
3. Owns a smartphone capable of downloading and running apps.
4. Willing and able to complete enrollment procedures and allow for review of medical records for two years to look for suicide attempts
5. Able to understand the nature of the study and provide written informed consent

6. Able and willing to provide at least two verifiable contacts for emergency purposes (Verification and update of emergency contacts will occur within 24 hours of all study visits and as needed at the discretion of the PI)

5.2 PARTICIPANT EXCLUSION CRITERIA

1. Patients who have untreated psychosis or are treated but still have active psychosis
2. Patients who upon examination appear to be impaired by the use of alcohol or other substance(s) at the time of evaluation for inclusion in the study.
3. Patients who sign, or have signed, an informed consent form to participate in any clinical research, trial, investigation, study, or activity that is or will be ongoing while the patient is a participant in this study, except as authorized by the Principal Investigator.
4. Patients who upon clinical examination are cognitively impaired or whose cognitive performance may adversely impact the integrity of the data.
5. Patients with a medical condition that, in the opinion of the PI, may compromise, interfere, limit, effect or reduce the subject's ability to complete a study of 104 weeks duration or may adversely impact the safety of the subject or the integrity of the data. Examples of considerations include terminal illness, psychosis, severe or unstable medical condition.

6. PARTICIPANT RECRUITMENT

The PI will be onsite and will work with hospital staff to identify eligible participants. Eligible participants will be invited to participate in the study while they are patients at the hospital site. The study staff will include licensed mental health professionals (i.e., 'research assessors') who will be trained by the investigative team (see section 10.0) and are experienced in working with suicidal patients. The PI will obtain permission to interact with the prospective participant by consulting with the assigned nurse. It is anticipated that these recruitment procedures will be effective, as the selected sites will have infrastructure that is supportive of research. Thus, from the pool of potential participants it is expected that 391 participants will be enrolled into the study.

7. STUDY ASSESSMENTS AND PROCEDURES

7.1 ASSESSMENTS

The assessments for this study are shown in Table 1. Baseline assessments are done in person at the Screening or Baseline visit. All assessments after Day 0 will be administered over the phone with site staff. These assessments are consistent with the set of assessments used by other studies (Rudd, et al. 2015; Brown, et al. 2005). All assessors will be blinded to participant treatment assignment. Additional details as follows.

Assessment	Construct	Description	Study Week (W)
**Primary Effectiveness Outcome**			
Suicide Attempt Self-Injury Interview (SASII)	Suicide attempts	Two methods will be used to assess suicide attempts: a) SASII and b) medical record review. The incidence of suicide-related behavior (actual, interrupted, aborted, preparatory behaviors) is of interest, but actual attempts is the primary effectiveness outcome. If either the **SASII** clinical interview or the medical record review indicates a suicide attempt,	Screening, W4, W8, W12, W24, W52, W78, W104

Assessment	Construct	Description	Study Week (W)
		participants will be coded as having attempted suicide during the timeframe assessed. For suicide attempts, the SASII will assess the method used, intent to die, highest level of medical treatment received, and greatest potential lethality of all self-directed violence in a specified time period (Linehan et al., 2006). A lifetime version will be administered at baseline. A recent version assessing only the time since the last assessment will be administered at future assessments.	
Medical Record Review		Clinical interviews will be supplemented by a medical record review to identify medically documented SAs during follow up that may have been missed in our interviews because they were not reported by participants or were unknown due to participant nonresponse or unavailability.	
**Secondary Effectiveness Outcome**			
Scale for Suicide Ideation (SSI)	Suicide ideation	Suicide ideation will be assessed with the clinician reported outcome, the Scale for Suicide Ideation (SSI). The SSI is a semi structured interview that assesses the intensity of various thoughts, urges, and attitudes about suicide during the past week. The SSI reliability and validity are well supported (Beck et al., 1979; Brown, 2002). The SSI has been standardized with adult psychiatric patients in the inpatient (Beck et al., 1985) and outpatient settings (Beck, Brown, & Steer, 1997). The SSI has been utilized in a variety of settings such as primary care practices, emergency rooms, rehabilitation programs, and private practice etc. (Brown, 2002). Reliability data show internal consistency with Cronbach coefficient alpha at 0.88 (Heisel & Flett, 2006) and high interrater reliability with correlations at 0.83 (Beck et al., 1979). Additionally, there is evidence that the SSI has predictive validity. Patients with a higher SSI score are more likely to die by suicide than those with lower scores (Brown et al., 2000). Lastly, the SSI is used to establish construct validity for related scales (Posner et al., 2011). Thus, the SSI is included in the list of recommended instruments in a number of toolkits: the Joint Commission suicide prevention resources (The Joint Commission, 2018); the NIH PhenX Toolkit (Hamilton, et al., 2011); and the National Institute of Mental Health (NIMH) toolbox.	Screening, W4, W8, W12, W24, W52, W78, W104
Beck Depression Index (BDI)	Depression Severity	Depression severity will be assessed with the 21-item self-report BDI-II (Beck, Steer & Brown, 1996). The BDI-II is a measure that taps major depression symptoms according to diagnostic criteria listed in the Diagnostic and Statistical Manual for Mental Disorders (American Psychiatric Association, 2000). Items are summed to create a total score. Higher scores indicate higher levels of depression. Studies have reported good internal consistency and test-retest reliability for the BDI-II across different populations, including community (Gomes-Oliveira et al., 2012; Kojima et al., 2002; Segal et al., 2008; Wang et al., 2013) adolescent and adult clinical outpatients (Grothe et al., 2005) and adult clinical inpatients (Subica et al., 2014). Criterion-based validity has also shown acceptable sensitivity and specificity of the BDI-II	Baseline, W4, W8, W12, W24, W52, W78, W104

Assessment	Construct	Description	Study Week (W)
		for detecting depression, supporting its clinical utility (Osman et al., 2008).	
Beck Hopelessness Scale (BHS)		Severity of hopelessness will be assessed with the 20-item BHS (Beck & Steer, 1988; Beck, Weissman, Lester, & Trexler, 1974). The scale questions are presented in a true-false format. This scale was designed to assess the degree to which a person has negative future expectancies. Examinations of reliability have demonstrated high internal consistency (e.g., Kuder-Richardson estimate = .93) and adequate test-retest reliability (Beck & Steer, 1988; Katz, Katz, & Shaw, 1999).	Baseline, W4, W8, W12, W24, W52, W78, W104
Clinical Global Impression severity subscale (CGI-S)	Clinical improvement	Clinical improvement will be assessed with the Clinical Global Impression severity subscale (CGI-S), which provides a reliable and valid summary measure of a participant illness that takes into account the participant history, psychosocial circumstances, symptoms, behavior, and functional impairment (Guy, 1976). The CGI S evaluates the severity of psychopathology on a scale of 1 (normal, not at all ill) to 7.	Baseline, W24, W52, W78, W104
Clinical Global Impression change subscale (CGI-C)	Clinical improvement	Clinical improvement will also be assessed with the Clinical Global Impression change subscale (CGI-C). It assesses how much the participant's illness has improved or worsened relative to their baseline state at the beginning of the intervention on a scale ranging from 1 (very much improved) to 8 (very much worse).	W12, W24, W52, W78, W104
**Mechanisms of Action**			
Monetary Choice Questionnaire (MCQ)	Impulsive decision making	Impulsive decision making will be assessed using the Monetary Choice Questionnaire (MCQ), a self-report questionnaire that presents a series of 27 monetary choices, each of which includes a smaller, immediate monetary reward and a larger, delayed monetary reward (Kirby et al., 2019). Higher values indicate a preference for immediate rewards and impulsivity. MCQ is temporally stable and has been shown to be associated with suicidal behaviors (Dombrovski et al., 2011, 2012).	Baseline, W12, W24, W52, W78
Cognitive Emotion Regulation Questionnaire (CERQ)	Emotion regulation	Emotion regulation will be assessed using the Cognitive Emotion Regulation Questionnaire (CERQ) self-report questionnaire that assesses a range of psychological strategies used in response to emotionally threatening or stressful events (e.g., self-blame, rumination, positive reappraisal). The CERQ is a reliable and valid measure of emotion regulation, and its subscales correlate with emotional distress and psychopathology in the expected directions (Garnefski, N., Kraaij, V., 2007).	Baseline, W12, W24, W52, W78
**Blinding Assessment**			

Assessment	Construct	Description	Study Week (W)
	The blinding index (BI)	The blinding index (BI) will be calculated by asking participants: "Do you think you received a suicide prevention app? Why?: (1) Yes (2) No (3) I don't know".	W104
**Perceptions of the app and treatment**			
Credibility/ Expectancy Questionnaire (CEQ)	App credibility	App credibility will be assessed using the Credibility/Expectancy Questionnaire (CEQ), a 6 item self-report scale that asks respondents to rate their perceptions about probable helpfulness of a treatment/intervention. CEQ reliability and validity are supported (Devilly, et al., 2000), and is used to measure perceived legitimacy of psychosocial and behavioral treatments.	W12, W104
System Usability Scale (SUS)	App acceptability and usability	App acceptability and usability will be assessed using the System Usability Scale (SUS; Bangor et al, 2008), a 10-item self-report scale that assesses a range of perceptions and opinions about a device usability (e.g., perceived complexity, ease of use, user friendliness). Item responses are summed and can be converted to a score ranging from 0 to 100, with scores above 68 indicating above average usability. Scores can be normalized to compare usability derived from hundreds of products.	W4, W8, W12
**Covariates**			
DSM-5 Self-Rated Level 1 Cross-Cutting Symptom Measure	Severity of psychiatric symptoms and psycho-pathology (SPS&P)	Severity of psychiatric symptoms and psychopathology (SPS&P) will be measured using the DSM-5 Self-Rated Level 1 Cross-Cutting Symptom Measure, a 23-item self-report scale of symptoms that cut across 13 diagnostic domains (e.g., depression, anger, mania, anxiety, psychosis, etc.). Respondents are directed to rate the intensity of each symptom within the past 2 weeks on a 5-point scale ranging from 0 (none/not at all) to 4 (severe/nearly every day).	Baseline, W24, W52, W78, W104
Insomnia Severity Index (ISI)	SPS&P/ insomnia	SPS&P will also be measured with the Insomnia Severity Index (ISI), a brief screening assessment tool designed to evaluate insomnia. The ISI is one of the most widely used assessment instruments in clinical studies of insomnia.	Baseline, W24, W52, W78, W104
Suicide Cognitions Scale (SCS)	SPS&P/suicide specific thoughts and beliefs	SPS&P will also be measured with a shortened 18-item version of the Suicide Cognitions Scale (SCS) as an indicator of current and future risk for suicidal thoughts and behaviors. SCS- is designed to assess suicide-specific thoughts and beliefs.	Baseline, W24, W52, W78, W104
Life Events Checklist (LEC)	Trauma exposure	Trauma exposure will be assessed with the Life Events Checklist (LEC) a self-report measure designed to screen for potentially traumatic events in a respondent lifetime.	Baseline
Adverse Childhood Experiences Questionnaire (ACE)	Trauma exposure	Trauma exposure will be assessed with the Adverse Childhood Experiences Questionnaire (ACE) a 10-item self-report measure developed to identify childhood experiences of abuse and neglect.	Baseline

Assessment	Construct	Description	Study Week (W)
Cornell Services Index (CSI)	Treatment utilization	Treatment utilization will be assessed using the Cornell Services Index (CSI), an interview-based method that assesses engagement in mental health treatment (e.g., outpatient psychotherapy, inpatient hospitalization).	Baseline, W4, W8, W24, W52, W78, W104
	Medication use	Medication use will be recorded at the baseline assessment using a combination of medical record review and interview. The use of ongoing and new medications will then be noted at each following study visit. The following information will be recorded for each medication: name, indication, dose, frequency, start + end date.	Baseline, W4, W8, W24, W52, W78, W104
	Demographic	Interviewer will ask participants their Age, Sex, Race/Ethnicity, Sexual Orientation, Education, Marital Status.	Baseline
	Frequency of app use	Frequency of app use will be tracked by downloading metadata from each participant user profile. We will extract the following use metrics: date of enrollment, number of sessions engaged (e.g., completing worksheet, listening to relaxation script), and number of sessions completed. Note: Participants may continue to use the app to review completed sessions and activities on their own after the 12 sessions are complete.	W4, W8, W24, W52, W78, W104
	Current and past psychiatric diagnoses	Current and past psychiatric diagnoses will be obtained by reviewing admission reports and electronic medical records.	Screening
**Adverse Events**			
Columbia-Suicide Severity Rating Scale (C-SSRS)	Adverse events	Adverse event monitoring will also be assessed with the Columbia-Suicide Severity Rating Scale (C-SSRS). The CSSRS is a semi structured, rater based interview to assess the severity and intensity of suicidal ideation and behaviors (Mundt et al., 2013; Posner et al., 2011; FDA, 2012). The scale has four constructs: severity of ideation, intensity of ideation, suicidal behavior, and suicidal behavior lethality subscales.<br><br>Similar to previous research (Comtois et al., 2019), results from the SASII interview will be coded to match the C-SSRS, which is consistent with FDA guidance for tracking suicide as an adverse event (Posner et al., 2011; FDA, 2012).	Baseline, W4, W8, W24, W52, W78, W104
**Other Measures**			
	Participant Information	Form to collect demographic, medical history and contact information. The contact info for each participant and 2 close contacts of the participant will be collected by the site staff. Detailed information will also be collected about other community contacts. Thus, if participants cannot be contacted, family, friends, clergy, probation officers, and mental health workers will be contacted by research staff with permission from the participants. These contacts will be confirmed within 24 hours of each assessment.	Baseline

Assessment	Construct	Description	Study Week (W)
	Medical History	Medical diagnoses will be evaluated during screening to determine eligibility	Screening

7.2. INFORMED CONSENT

The participant must personally sign and date the latest approved version of the informed consent form before any study specific procedures are performed. Written and verbal versions of the informed consent will be presented to the participants detailing no less than: the exact nature of the study; the implications and constraints of the protocol; the known side effects and any risks involved in taking part. It will be clearly stated that the participant is free to withdraw from the study at any time for any reason without prejudice to future care, and with no obligation to give the reason for withdrawal.

The participant will be allowed as much time as needed to consider the information, and the opportunity to question the PI, his/her General Practitioner (GP) or other independent parties to decide whether they will participate in the study. Written consent will then be obtained by means of a dated signature of the participant and a dated signature of the person who presented and obtained the informed consent. The person who obtained the consent must be suitably qualified and experienced and have been authorized to do so by the PI. A copy of the signed Informed Consent will be given to the participant. The original signed form will be retained at the study site.

7.3 SCREENING AND ELIGIBILITY ASSESSMENT

Everyday the PI or designee will check with hospital staff to see if new potential participant(s) have been admitted. Patients will be told about the opportunity to participate in the study and informed by the research therapists about the voluntary nature of their participation and that their data will remain confidential. Once the patient agrees to participate, the PI will review the patient’s medical history and psychological evaluation with the patient and gather additional information to determine whether the patient meets eligibility criteria. After informed consent is given, research therapists will administer a structured clinical interview focused on suicide attempt history.

Prior to enrollment, the following information will be collected:

- Demographic Information
- Medical History
- Concomitant Medications/psychotherapy

Following enrollment, participants will receive instructions on how to install the app on their phone.

7.4 PARTICIPANT ENROLLMENT

To preserve participant blinding, participants will be informed that they will be assigned to “one of two digital interventions.” Participants will be informed that both interventions include some combination of supportive listening, education about crisis services, software services and referrals to treatment services. Once the patient agrees to participate, the PI will review the patient’s medical history and psychological evaluation with the patient and gather additional information to determine whether the patient meets eligibility criteria. The site PI will obtain informed consent from participants using procedures that will be Internal Review Board (IRB) approved. After informed consent is given, research therapists will administer a structured clinical interview focused on suicide attempt history. Upon completion, the research therapist will execute a randomization procedure and the participant will be randomized to the Aviva + TAU or sham +

TAU group (See Section 7.5). Following randomization, participants in both groups will receive an access (SMS) link and instructions on how to install the Aviva or sham apps on their phone. The app is to be downloaded before the end of the study visit.

7.5 RANDOMIZATION CRITERIA

Participants who meet the eligibility criteria will be assigned a participant number and will then be randomly assigned to the Aviva + TAU or sham + TAU groups. A computerized modified minimization randomization procedure within the electronic Case Report Forms (eCRFs) will be used. Eligible participants will be randomized based on study site, sex, race/ethnicity and whether they have had no attempts, single attempts or multiple suicide attempts. This adaptive randomization method has been shown to be superior to stratified randomization in producing balance for separate prognostic variables, particularly when the number of strata is large compared with the number of subjects. Once the participant is randomized the site staff will make sure that the participant has properly loaded Aviva ***or*** sham app onto his/her phone. The PI or designee will confirm that the app has been downloaded and, if the participant is having difficulty, assist them beginning the onboarding session.

7.6 CONCOMITANT MEDICATIONS/PSYCHOTHERAPY

All concomitant medications will be recorded at the Baseline Visit. The use of ongoing and any new medications will then be noted at each following study visit. All medication use will be captured by research assessors from participants in follow-up visits in the source documents and on the appropriate eCRF page. The following information will be recorded for each medication: name, indication, dose, frequency, start date, end date. Participation in outpatient psychotherapy will also be recorded with the Cornell Services Index (CSI). Additionally we will collect data from the psychotherapy providers about attendance, medication, and anything related to the patient safety.

7.7 RETENTION METHODS

The researcher staff will collect contact information for participants, including participant’s email addresses, telephone numbers and addresses for home and employment locations as well as contact information of their TAU provider. The study staff will also collect contact information (telephone number and email) for two individuals that the participant identifies as people who will always know how to reach them. Study staff will also ask study participants to provide contact information for their outpatient mental health care provider and then verify that contact information. Detailed information will also be collected about other community contacts. Thus, if participants cannot be contacted, family, friends, clergy, probation officers, and mental health workers will be contacted by research staff with permission from the participants. These contacts will be confirmed within 24 hours of each assessment. Study staff will also follow up with outpatient mental health care providers using the same cadence as study participants' follow up visits: at weeks 4, 8, 12, 24, 52, 78 and 104. Participants will be assured of confidentiality when they share this data (and throughout the study). At enrollment, site staff will ask participants to report their preferred method of communication (i.e., email, text message, or phone call) in order to use the notification strategy that they’re most likely to pay attention to. The app will collect participant usage metrics with the goal of improving participants’ adherence to this recommended treatment. If participants miss a session, these metrics will allow Aviva to send prompts to participants until they initiate or reschedule a session. Messages will be sent to participants, via their preferred method of communication, which will include reminders to engage with the study, motivating statements (e.g., Great job!) for participating and encrypted emails to acknowledge important milestones in their participation. When participants are not attending outpatient mental health

sessions the Sponsor will send participants correspondence, based on the participants preferred method of contact, to remind them of their treatment plan.

7.8 STUDY INTERVENTION COMPLIANCE

Compliance will be measured with weekly reports by the study sponsor. The study sites will be notified of participant non-compliance.

8. STUDY DISCONTINUATION AND PARTICIPANT DISCONTINUATION/WITHDRAWAL

8.1 DISCONTINUATION OF STUDY SITE OR STUDY

The Sponsor has the right to terminate the study at any time. Reasons for site discontinuation include:

- Conduct of the study at this site is not in accordance with the GCP guidelines
- Repeated failure to complete documentation (source documents or eCRFs) / quality of data
- Failure to obtain Informed Consent
- Failure to report Serious Adverse Events (SAEs) within 24 hours of knowledge
- Repeated protocol deviations
- Failure to enroll an adequate number of subjects

The Study Sponsor also reserves the right to discontinue the entire study. Reasons the entire study could be discontinued include, but are not limited to:

- SAEs or if special circumstances concerning the Aviva therapy or the company itself occur, making further treatment of subjects impossible

In the event of site or study discontinuation, the study investigator(s) will be informed of the reason for study termination. Study materials must be returned, disposed of or retained as directed by the Study Sponsor.

8.2 PARTICIPANT DISCONTINUATION/WITHDRAWAL FROM THE STUDY

Each participant has the right to voluntarily withdraw from the study at any time. To do so, he/she may notify a member of the research team of their decision to withdraw during study assessments or may contact the study PI at the phone number(s) listed on the informed consent document. Participants may stop participation without notifying a member of the research team (e.g., cease interaction with the app). Participants may be withdrawn from the study by the PI if they are unable to be contacted (lost to follow-up), become incarcerated, or are determined to be adversely affected by the study procedures. Participants who withdraw early from the study will have their access to the app revoked.

In addition, the investigator may discontinue or withdraw a participant from the study at any time if the investigator considers it necessary for any other reason including, but not limited to:

- Ineligibility (either arising during the study or retrospectively having been overlooked at screening)
- Significant protocol deviation
- Significant non-compliance with protocol or study requirements
- An adverse Event (AE) which requires discontinuation or results in inability to continue to comply with study procedures
- Participant are unable to be contacted (lost to follow-up)
- Participant becomes incarcerated
- Participant is determined to be adversely affected by the study procedures

Per the FDA regulations, when a subject withdraws from a study, the data collected on the subject to the point of withdrawal remains part of the study dataset and may not be removed. However, (as described in the informed consent) the investigator may ask a subject who is withdrawing

whether the subject wishes to provide continued follow-up and further data collection subsequent to the withdrawal from the treatment portion of the study. Staff will make every effort to collect final study visit assessments prior to withdrawal. Withdrawn participants will not be replaced for this study. The reason for withdrawal will be recorded in the source documents and eCRF. If the participant is withdrawn due to an AE, the investigator will arrange for follow-up visits or telephone calls until the AE has resolved or stabilized.

9. SAFETY

9.1 SAFETY HYPOTHESIS

Aviva is safe for use by individuals with a previous history of suicidal ideation and attempts.

9.2 ADVERSE EVENTS

An AE is any untoward medical occurrence, unintended disease or injury, or any untoward clinical sign (including an abnormal laboratory finding) in subjects, users, or other persons, regardless of whether or not it is related to the investigational medical device.

- This definition includes adverse events related to the medical device of this investigation or comparator.
- This definition includes adverse events related to the procedures involved (any procedures in the clinical investigational plan)
- For adverse events involving "users or other persons", this definition is restricted to adverse events related to the medical device of this investigation.

Adverse events are classified and tabulated by relationship to procedure or device, severity, and body system. Serious Adverse Events (SAEs), deaths and Unanticipated Adverse Device Effects (UADEs) will be listed separately.

The following should not be recorded as AEs:

- Pre-planned procedure unless the condition for which the procedure was planned has worsened from the first study-related activity after the subject has signed the informed consent form.
- Pre-existing conditions found as a result of screening procedures. These should be recorded as medical history/concomitant illness.

Adverse events are graded on a 3-point scale and reported as indicated on the eCRF. The intensity of an adverse experience is defined as follows:

- **Mild:** Discomfort noticed, but no disruption to daily activity
- **Moderate:** Discomfort sufficient to reduce or affect normal daily activity
- **Severe:** Inability to work or perform normal daily activity

Study Relationship: the relationship of an adverse event to participation in the study will be assessed by the investigator as follows:

- **Definitely Related**: Clear-cut temporal association and no other possible cause.
- **Probably Related:** Clear-cut temporal association and a potential alternative etiology is not apparent.
- **Possibly Related:** Temporal association is less clear and other etiologies are also possible.
- **Not Related:** There is no temporal association and/or evidence exists that the event is definitely related to another etiology

All adverse event reports are filed as required by IRB and FDA regulations. For all adverse events (whether device-related or not), all sections of the appropriate Adverse Event Form(s) must be completed. **In this study, all adverse events are collected starting from the time informed consent has been signed.**

AE data will be collected on an adverse reporting form at the follow up assessments. Research assessors will ask participants, "Have you had any physical or mental health problems since our last call?" If the participant responds "yes", research assessors will note the specific event, event start and end dates, event intensity (whether the event was associated with participation in the study, actions taken to resolve the event (e.g., hospitalization) and the outcome of those actions (e.g., resolved). All adverse events (AEs) will be coded using the standardized Medical Dictionary for Regulatory Activities (MedDRA) central coding dictionary, version 24.1 or greater.

9.3 RATIONALE FOR IDENTIFICATION OF ADVERSE CLINICAL EVENTS

The proposed study protocol is based on previous trials of BCBT/CT-SP. Anticipated adverse clinical events are therefore derived from these previous trials. The BCBT/CT-SP developers have observed the following adverse events in their studies:

- suicidality
- hospitalization
- death by suicide

9.4 EXPECTED ADVERSE EVENTS IN PARTICIPANTS

Primary anticipated events therefore include:

- increased suicidal ideation
- hospitalization
- death by suicide

Any study participation related adverse events are to be followed until there is evidence of resolution or permanent change.

The determination of whether an adverse event is classified as a SAE or UADE is based on the definitions below, taking into account the clinical judgment of the investigator.

9.5 SERIOUS ADVERSE EVENTS

A SAE is any untoward medical occurrence that:

- Results in death
- Is immediately life-threatening
- Results in disability or permanent damage
- Requires intervention to prevent permanent impairment or damage
- Requires participant hospitalization or prolongation of existing hospitalization
- Is a congenital anomaly/birth defect, or
- Is any other serious or important medical event

Given the high risk of suicide attempt in this study population a missed follow-up visit, for collecting assessment data, will be treated as an SAE.

Important medical events that may not result in death, be life-threatening, or require hospitalization may be considered serious when, based upon appropriate medical judgment, they may jeopardize the participants' ability to participate and may require medical or surgical intervention to prevent one of the outcomes listed in this definition.

Planned hospitalization for a pre-existing condition, or a procedure required by the protocol, without serious deterioration in health, is not considered a SAE.

A SAE may or may not be considered related to study participation.

9.6 UNANTICIPATED ADVERSE DEVICE EFFECTS

An UADE is defined as any serious adverse effect on health or safety or any life-threatening problem or death caused by, or associated with, the device if that effect, problem or death was not previously identified in nature, severity or degree of incidence in the investigational plan; or any other unanticipated serious problem associated with the device that relates to the rights, safety or welfare of subjects.

9.7 REPORTING OF ADVERSE EVENTS

AEs observed during the course of this study, regardless of severity or relationship to the study participation will be recorded on the appropriate Adverse Event Form and reported to the Sponsor.

For sites and reporting to FDA, the procedures for handling and reporting/notification of SAEs shall be carried out in accordance with the applicable sections of 21 CFR Part 812 and local IRB requirements.

9.7.1 Investigator Safety Reporting Requirements

SAEs will be recorded in the Adverse Event Form and the event is to be reported to the Sponsor within 24 hours of knowledge of the event. Information not available at the time of the initial report must be documented in the Adverse Event Form within 24 hours of receipt of the new information. Substantiating data such as relevant hospital or medical records and diagnostic test reports should also be submitted by scan/email to the Sponsor.

An Investigator shall submit to the Sponsor and to the reviewing IRB a report of any UADE occurring during an investigation as soon as possible, but in no event later than 10 working days after the investigator first learns of the effect. The Investigator must adhere to all reporting requirements for his IRB and national authorities.

9.7.2 Sponsor Safety Reporting Requirements

SAEs and UADEs will be reported to the FDA, in accordance with 21 CFR Part 812.

In the event of a UADE, Sponsor will immediately conduct an evaluation of a UADE, and report the results of such evaluation to FDA and to all reviewing IRB's and participating investigators within see above comment. The Sponsor first receives notice of the effect. Thereafter, the Sponsor shall submit such additional reports concerning the effect as FDA requests.

9.8 REPORTING PERIOD

In this study, all adverse events are collected starting at the time informed consent has been signed. AEs are collected until the last study visit and exit from the study.

9.9 SAFETY MONITORING AND REPORTING

All observed or volunteered adverse events or suspected causal relationship to the investigational product will be reported as described above. For all adverse events, the PI will pursue and obtain information adequate to determine the outcome of the adverse event and to assess whether it meets the criteria for classification as a serious adverse event requiring immediate notification to the Study Sponsor or its designated representative. For all adverse events, sufficient information should be obtained by the investigator to determine the causality of the adverse event. The PI is required to assess causality. Follow-up by the investigator may be required until the event or its *sequelae* resolve or stabilize at a level acceptable to the investigator, and the Study Sponsor concurs with that assessment.

This study will involve a Data Safety Monitoring Board (DSMB) because this study will include participants who are at risk of attempting suicide. Oversight of the study is provided by the site PIs who will be actively involved in the conduct of the study. The PIs will lead ongoing and systematic reviews of data integrity, protocol adherence, and participant safety. Considering

the high risk and ethical concerns when doing research on suicide, the site PIs will ensure these two monitoring components are followed: 1) the clinical/safety monitoring of individual participants; 2) review of study data to assure data accuracy and consistency. DSMB members will include individuals who have extensive experience in clinical trials and suicide prevention. Site PIs will monitor data to determine whether study participation is leading to increased risk of harm to subjects by comparing rates and types of harm events across treatment arms. The DSMB will guide our team in assessing the risk of potential liability and matters of safety monitoring. During interim looks at the data for safety, the sponsor will continue to be blinded to condition, as will the assessors. The data will be tabulated by a CRO and forwarded to a DSMB for adjudication. There will be standard operating procedures for the DSMB meetings. Safety events/SAEs are clearly defined elsewhere in the protocol, with stopping rules (see section 11.5 STOPPING RULES). Safety events and SAEs will be reported to the DSMB for review by the DSMB in a timely manner (i.e. within 5 days). The study personnel and sponsor will remain blinded until all enrolled participants have completed the 104 week follow-up. If the DSMB requires the study be stopped (either for SAEs or positive outcomes/benefit), the study will be unblinded and a decision will be communicated to the Sponsor. The sponsor will notify the FDA.

9.9.1 DSMB Charter, Interim Analysis, and Statistician Blinding

An independent statistician will provide the DSMB with the tables and analyses for the DSMB meetings. The independent statistician will participate in the DSMB meetings as a non-voting member and will be a different person from the statistician who is a member of the DSMB. Appropriate firewalls will be in place so that all persons involved with the operations of the study remain blinded to study results (the sponsor and researcher assessors will be blinded), except if the DSMB recommends stopping the study. In that case, members of the executive committee of Oui, the study investigators may be unblinded. This information will be included in the finalized DSMB charter when it becomes available. The finalized DSMB charter will be available once the DSMB is constituted and the Statistical Analysis Plan (SAP) has been finalized. Avania, a clinical research organization, will play the role of the independent statistician. The charter and DSMB will be convened in advance of opening study for enrollment.

10. CLINICIAN TRAINING AND SUPERVISION

Overview

Oui Therapeutics or Consultants for the Study Sponsor, who are clinical psychologists with extensive experience administering and supervising assessment interviews within the context of clinical trials along with other experienced clinical trial professionals, will train the site PI’s on the full battery of assessments for this research and existing protocols. The PI’s will train the research therapists on the study protocol. The training will primarily focus on how to build rapport with the participant while they are in the hospital and establish a collaborative agreement to monitor suicidal ideation. The training will also include procedures for recruitment, enrollment, assessment and app installation, as well as safety protocols. The site staff will be trained to answer participant questions. If study participants self-identify changes in their condition, i.e. increased suicidal intent or behavior, the app will guide them to contact the PI for follow-up assessments. As part of their supervisory role, PIs are responsible for recruitment, enrollment, assessment, app installation, tracking outcomes, safety protocols, and ongoing participant care following enrollment.

Specific strategies for obtaining complete records

To obtain complete records before randomization, a research staff member at each site will be trained to collect information regarding inclusion/exclusion criteria. There will be a standardized form for the research staff to complete to document the presence or absence of inclusion and exclusion criteria and the source of the information. The form will contain specific

steps and procedures for collecting data from participant's medical record (e.g., search for self-injury and suicide attempt ICD codes [https://www.cdc.gov/nchs/data/nhsr/nhsr108.pdf]), the participant and the provider supervising the patient's care. A waiver of consent and HIPAA will be requested so that the study team can review medical records of potential participants to determine whether they are likely to be eligible for the study and the consent form will explain the procedures that were used. A research staff member will conduct the medical record search prior to the baseline assessment, then provide the list of identified events to the interviewer prior to baseline assessments.

To obtain complete medical records after randomization, structured follow-up assessments and electronic medical record reviews will be conducted by study staff.

- Follow-up assessments: The follow up assessments will be clinician-administered interviews designed to assess incidence of suicidal behaviors, adverse events and other variables of interest during the target assessment period. To reduce likelihood of missed follow-up assessments specific strategies for tracking will be used such as (1) utilization of research staff ("study managers") to obtain detailed contact information regarding participants' family, friends, clergy, probation officers, and mental health workers and (2) utilization of research staff who will use medical records and the participants' healthcare providers, whenever possible, to track participants over time and to assist with scheduling follow-up assessments. If participants cannot be contacted, family, friends, clergy, probation officers, and mental health workers will be contacted by research staff, with permission from the participants.

- Electronic Medical Records: Review of electronic medical records will be conducted to identify suicide attempts that might have been missed during assessment interviews (e.g., participants who drop out early or miss follow-up assessments) as well as to quantify use of mental health and medical services by participants (e.g., frequency and type of appointments, frequency and duration of inpatient hospitalization, etc.). We will conduct a search within the medical records for self-injury and suicide attempt ICD codes (https://www.cdc.gov/nchs/data/nhsr/nhsr108.pdf).

11. STATISTICAL CONSIDERATIONS

11.1 STATISTICAL PROCEDURES AND DATA ANALYSIS

11.1.1 Primary Effectiveness: Definition of Success (or Failure) of the Endpoint

The time to first suicide attempt after randomization is the primary endpoint. The study will be a success if the primary endpoint is statistically significant in favor of Aviva (compared to sham app) based on time to suicide attempt, a time-to-event variable. At 90% power, fifty suicide attempts (i.e., event) are expected while following 391 participants for 104 weeks. In anticipation that treatment effects may be observed before 50 suicide attempts occur, the primary endpoint will be tested via a group sequential test with K = 4 Looks using an O'Brien-Fleming like alpha spending function boundary. A non-binding futility boundary is incorporated in this design, also with an O'Brien-Fleming boundary. The Type I error level alpha will be protected at level 0.025 one-sided (which is equal to 0.05 two-sided).

Study failure will be defined as lack of a statistically significant finding for suicide attempts in favor of Aviva. Details are provided below.

11.1.2 Rationale

It is anticipated that suicide attempts will reflect benefits of Aviva over sham that are discernable at 104 weeks after randomization. A group sequential design is appropriate because it would be prudent to stop the study earlier for significant effectiveness in the case where the time of participants who attempt suicide is significantly less in the Aviva group compared to the sham group (and thus reducing risk of suicide shortly after discharge).

11.1.3 Justification of sample size-suicide attempts

Power and sample size calculations were made in nQuery, Version 8.7.2. Initial sample size calculations for suicide attempts were based on Rudd et al. (2015). A total sample size of 391 (with 52 events) is required to achieve 90% power to detect a hazard ratio of 0.3826 (for survival rates of 0.9240 in the Aviva group [group 1] and 0.8124 in sham app group [group 2] at 18 months), using a one-sided log rank test with 2.5% significance level (i.e., p = 0.025) assuming that the survival rates are exponential and that the total study time is 104 weeks (24 months) with 52 weeks (12 months) accrual.

These results assume that the group sequential design has 3 interim sequential tests (4 total looks including final analysis). The O'Brien-Fleming spending function is used to determine the effectiveness test boundary. See Attachment C: Power Calculation and Group Sequential Design for Aviva Trial – Suicide Attempts (Mathematical Formulations) for full details of sample size calculations.

11.1.4 Group Sequential Design for Suicide Attempts

Although the dropouts will not provide the clinical interview information at study end, it will be possible to review hospital records to obtain information regarding suicide attempts by participants (this will be confirmed during informed consent). We will randomize 196 participants to the (Aviva) treatment group and 195 participants to the active control (sham) group and expect to have suicide attempt data for 100% of these participants. We investigated several reasonable variations of the group sequential design and determined that the following is most reasonable. We will test at the 0.025 significance-level (one-sided). The group sequential design has 3 interim sequential tests (4 total looks including final analysis). The looks will occur when 60%, 75%, 90% and 100% of the expected events (i.e., suicide attempts) have occurred. The O'Brien-Fleming spending function is used to determine the effectiveness test boundary. The group sequential design boundaries (on a z scale) are presented in Attachment C.

The power of this GSD is 90%.

Survival analyses will be conducted using the log-rank test for the effectiveness of the intervention on the time to the first suicide attempt after randomization while controlling for censoring effects due to the differential length of follow-up or the completion of follow-up without a suicide attempt. Time to suicide attempt will be measured by calculating the total number of days from enrollment to the first suicide attempt. For participants without a suicide attempt, the total number of days since enrollment to the last assessment will be calculated.

While every effort will be made to track suicide attempts (SA), there may be some missing data. Consistent with standard statistical procedures, missing data are included in the time-to-event analysis as censored observations, with the censoring time at the last contact time with the patient (Friedman et al., 2015).

Estimates of participants making at least 1 subsequent suicide attempt before study end and reattempt-free probabilities at any time point will be derived by the Kaplan-Meier method. The

between-group difference in the proportion of participants making at least 1 repeat suicide attempt by 104 weeks will be evaluated using the asymptotic properties of the Kaplan-Meier estimators of survival probabilities. Although we do not anticipate dropout (because suicide attempt data can be collected via multiple means), this method was chosen to account for dropouts based on the Intent-to-Treat (ITT) principle.

11.1.5 Secondary Endpoints

SSI will be the first secondary endpoint. Mean changes from baseline will be analyzed using a restricted maximum likelihood (REML)-based repeated measures approach. Analyses will include the fixed, categorical effects of treatment, investigative site, visit, and treatment-by-visit interaction, as well as the continuous, fixed covariates of baseline score and baseline score-by-visit-interaction. Although we will provide training to minimize differences in site, it is recommended the investigative site be included in models of clinical trial data to account for any chance that site-specific subjectivity may influence outcomes (Ting, 2018). A treatment-by-visit interaction allows the effects of the treatment to differ by visit (Ting, 2018). It is reasonable to model the possibility that treatment effects differ by visit because each "dose" of treatment includes different content, study follow-up occurs after treatment has ended, and the study follow-up occurs over an extended period of time. The score-by-visit interaction accounts for the possibility that the impact of baseline to responses at different visits could be different (Ting, 2018). An unstructured (co)variance structure will be used to model the within-subject errors. The Kenward-Roger approximation will be used to estimate denominator degrees of freedom and adjust standard errors. Significance tests will be based on differences of least-squares means using a two-sided $\alpha=0.05$ (two-sided 95% confidence intervals). Analyses will be implemented using SAS Version 9.4. For SSI, the primary treatment comparison will be the contrast between treatments at 24 weeks. Exploratory models will examine SSI at study end. The same model will be applied for exploratory testing intervention effects on the BDI-II and BHS at study end.

The hypothesized mediators are impulsive decision-making and emotion regulation. In the mediation model, treatment condition will be the independent variable and, SAs (defined as a binary variable) will be the outcome variable. We will use regression parameters and their standard errors with 10,000 bootstrapped resamples as recommended by Preacher et al. (2004) to test for mediation. Mathematically, the mediation path will be defined as the product of coefficients for the path from the independent variable to the mediator (path a) and the path from the mediator to outcome variable (path b). Thus, we will test the statistical significance of a*b.

11.2 MINIMUM CLINICALLY IMPORTANT DIFFERENCE (MCID)

11.2.1 Suicide Attempts

Suicide Attempts are one of the leading causes of morbidity in the US health care system with more than 1,400,000 attempts in 2018 and are the best predictors of further suicide attempts and death from suicide. The ability to prevent even just one suicide attempt reflects the ability to 1) prevent a tragedy that affects not just the individual, but also their families and communities and 2) potentially reduce costs of healthcare system utilization related to suicide attempts with a relatively low-cost intervention. **There are currently NO FDA approved products that are suitable for a broad audience of adults.** Given the importance of suicide attempts and the binary nature of attempts any statistically significant difference between the control and treatment arms would be meaningful for clinicians and their patients. While studies using face-to-face implementation of the techniques used by Aviva have shown more than 40% reduction in incidence of suicide attempts relative to a control condition (Brown et al., 2005, Bryan et al., 2017, Rudd et al., 2015) we do not feel patients should be kept from an intervention that shows a lower, but significant, chance of decreasing an attempt. Given the lack of any FDA approved or cleared

products available, any statistically significant result at the 0.025 level of significance (one-sided) is clinically important and has the potential to have meaningful impact both at the societal and individual level, particularly in this moderately sized clinical study. Statistical significance is important at this stage because a suicide attempt is a clinically important life event. The clinical significance of the findings of studies using our proposed techniques is evidenced by the adoption of these techniques by the Joint Commission (The Joint Commission, 2016) and the U.S. Department of Veterans Affairs (U.S. Department of Veterans Affairs, 2008).

11.2.2 Suicidal Ideation

Effect size will be used as a measure of minimally clinically significant difference (MCID). This study has been designed to detect an effect size of 0.50 for suicidal ideation (assessed through the SSI), which is both a moderate effect size and a clinically significant amount. To calculate this expected effect size, the standard deviations presented in research that used the same suicide prevention techniques proposed to implement with Aviva (Bryan et al. 2017) were reviewed. The clinical significance of the findings of studies using the proposed techniques (Brown et al., 2005, Bryan et al., 2017, Rudd et al., 2015) is evidenced by the adoption of these techniques by the Joint Commission (The Joint Commission, 2016) and the U.S. Department of Veterans Affairs (U.S. Department of Veterans Affairs, 2008).

To support the effectiveness of Aviva for SI, it will be beneficial to demonstrate what constitutes a clinically meaningful within-patient change in scores (i.e., improvement threshold), from the patient perspective, in addition to an MCID across all patients. To estimate a clinically meaningful within-patient change, the following scales will be included to support a clinically meaningful within-patient change analysis (an improvement score of 1 unit on the CGI-C scale), and aim to achieve statistically significant and clinically meaningful differences in a responder analysis between the treatment and control groups for the two scales identified below:

- Clinician Global Impression of Severity (CGI-S) administered at baseline, 24 weeks and at study end
- Clinician Global Impression of Change (CGI-C) administered at 24 weeks and at study end

11.3 ANALYSIS SETS, SENSITIVITY ANALYSES AND SUBGROUP ANALYSIS

The analysis sets will be:

1. ITT: all participants randomized into our study.
2. Per-protocol population (PP): The PP population will include all ITT participants with no major protocol deviations. Major protocol deviations are defined as follow:
    a. Participant did not complete informed consent procedures
    b. Participant is not randomly assigned to a treatment condition
    c. Participant is enrolled in the wrong treatment arm (i.e., a different treatment arm than the one to which they were randomly assigned)
    d. Enrolled participant did not meet eligibility criteria
    e. Participant did not complete onboarding. Onboarding entails downloading the app and completing session 1
    f. Participant is unblinded due to staff error
3. The Safety Analysis population set consists of all randomized patients that have begun treatment, as defined by completion of the onboarding process. This set will be used for all safety analyses using treatment actually received. Since onboarding occurs prior to discharge, it is assumed that both populations will be identical.

The sensitivity analyses will proceed in the following manner: the primary analysis will be repeated in the per-protocol population and the Cox proportional hazards model will be carried out on the ITT population to investigate the robustness of the finding for suicide attempt in the presence of potentially important factors such as sex, race/ethnicity, and site. The ITT will be used for the primary and secondary endpoint analysis either here or in the sections above where each is discussed. Associated Wald tests will be conducted using a significance level of .05 (2-sided) to test the null hypothesis that the two time-to-event distributions are the same for the Aviva and sham groups at any time point. We will conduct subgroup analysis based on the stratification factors used in our randomization (sex, race/ethnicity and previous suicide attempt history [i.e., whether participants had no suicide attempts, a single attempt or multiple suicide attempts] and investigative site). We will include sex, race/ethnicity because differences in rates (or increases in rates) have been observed for each. Regarding sex, the age adjusted suicide death rate for males (22.79 per 100,000) is higher than the rate for females (6.18 per 100,000; CDC, 2020). For race/ethnicity, American Indian/Alaska Native adults are at highest risk for past-year suicide attempts, followed by Black and Hispanic adults (CDC, 2020). Lastly, while all efforts will be made to standardize procedures across sites, it is possible that site differences may emerge. Thus, we will examine whether there are any treatment differences based on sites. Lastly, previous suicide attempt history is a strong predictor of future suicide attempts.

11.4 ADDITIONAL CONSIDERATIONS

Poolability analysis: Descriptive statistics will be provided by site to present an overall study summary. We will remove any sites with <10% of patients from the poolability analysis. Poolability analysis will examine site as a covariate in a Cox proportional hazards model for the primary endpoint. The site effect will be evaluated at a 0.15 level of significance. Due to the relatively small rate of expected events, we feel this is more appropriate than a site-by-treatment interaction term analysis. A similar analysis will be repeated to assess poolability of race, sex and age. Sites with fewer than 5 subjects from the poolability analysis.

Sites: To prevent the scenario of one site dominating the study, we will cap enrollment at 36% of the total enrollment at any one site in any one geographic region. We have based the proportion on the 4 sites that are currently identified as potential participating sites. At present, current patient flow at the 4 sites suggests it is not likely that more than 1 site would reach this threshold. Should we identify an additional 4 sites (bringing the total to 8), we will reduce the percentage to 20%. Center will be used as a nuisance factor in the analyses.

Additional time points: While we are primarily interested in outcomes at 104 weeks, at the conclusion of the study, additional time points will be investigated.

Prior to the start of the study, we will determine the subject and study level primary outcome data elements that can be reported from all participating sites. We will collect (and confirm) information about the data elements that can be reported from all participating sites in a systematic way through a Site Assessment Questionnaire and Site Qualification Visits. Formal training for the sites will be conducted at Site Initiation Visits.

Uniform methods for collecting safety and primary outcome data will be in place across each site. We will ensure implementation of these methods through the site selection process, training of staff and additional data collection procedures for participants who drop out or are lost to follow-up.

Site selection and evaluation

The site selection process will ensure that each site can provide the required safety and primary outcome data. Specifically, we will evaluate whether the site has appropriate technical capabilities and personnel training to ensure proper capture of data.

1. Identification of Sites: We will identify suitable clinical trial sites through a variety of sources (e.g., professional referrals, literature review and the Clinicaltrials.gov website).
2. Feasibility Assessment Questionnaire: Each site will complete a feasibility assessment questionnaire. The questionnaire was specifically designed for this study and will allow Oui to gather specific information on the site staff qualifications and experience as well as gain an initial understanding of the site set up and access for the monitor as well as an idea of the patient volume. Based on the responses to the Feasibility Assessment Questionnaire, the sites will be selected for Site Qualification Visit.
3. Site Qualification Visit: The Site Qualification Visit will be conducted either by web meeting or in person as is allowed by the site and current Covid conditions. If conducted by web meeting, a video tour of the facility will be requested. If a video tour cannot be conducted, the sponsor will request pictures of the facility. The Qualification Visit will be conducted by the study sponsor or designee. The visit will include a meeting with the proposed PI and Study Coordinator as well as any other relevant site staff. During the visit, the Sponsor or designee will obtain information on the IRB and predicted timelines, the contract process, the subject population, the staff resources available, as well as assess the sites interest in conducting the study.
4. Site Initiation Visit and Ongoing Monitoring: Site initiation visits and ongoing monitoring will ensure that the sites are conducting the study in a manner that ensures subject safety and protocol compliance and are able (and continue) to abide by the uniform methods for collecting safety and primary outcome data.

Staff training at study initiation

1. Trained/Designated Staff: Each site will have designated research staff who will be trained to collect the study outcomes.
2. Clear Definitions and Electronic Data Capture: We will use a structured method for obtaining all study outcomes. For variables assessed, there will be clear definitions. Staff will receive training in how to apply those definitions and record them in the study electronic data capture (EDC) system.

During study monitoring and support

1. Study Monitoring and Data Fidelity Checks: The sponsor or its representative may visit the study facilities at any time (remotely and /or in person) in order to maintain current and personal knowledge of the study through review of the records, comparison with source documents, observation and discussion of the conduct and progress of the study. The clinical site will permit trial-related monitoring, audits, IRB/IEC review, and regulatory inspection(s) by providing direct access to source data/documents.
2. Fidelity Checks: As part of study monitoring, we will conduct ongoing fidelity checks for the primary outcomes (and other variables of interest). During fidelity checks, records will be pulled randomly. If errors in coding the variables are observed, the nature of the error will be evaluated to determine whether study procedures need to be updated to improve clarity (though unlikely as we are using methodologies established in prior work) and/or staff require additional training in how to capture the study variables.

Procedures for participants who drop out or are lost to follow-up
If subjects drop out or are lost to follow-up, there will be agreements with the subject per the Informed Consent that the research staff can access different sources of information other than medical records. Additional sources of information will be the persons listed as individuals the research staff may reach out to if the research staff cannot contact the participants. We will also obtain permission to request medical records from other treatment facilities if the primary outcome or adverse event is reported at another treatment facility. See prior discussion regarding ensuring final study assessments be performed if possible before withdrawal of consent.

11.5 STOPPING RULES

A Data Safety and Monitoring Board (DSMB) will have oversight over the three stopping rules proposed below. Though we have proposed stopping rules within this protocol, the DSMB will finalize the stopping rules for safety. Safety Monitoring will be guided by previous research and study data to determine whether there is an excess of suicide attempts or death. Prior research has shown that, following a suicide attempt, up to half of the patients in treatment make another suicide attempt. If a suicide attempt occurs within the first year of treatment, on average a patient will attempt suicide 2.5 times. Monitoring of suicide attempts will occur by tracking hospitalization records, reviewing medical records, and carrying out interviews at all time points. Other specific strategies for tracking include (1) utilization of research staff ("study managers") to obtain detailed contact information regarding participants' family, friends, clergy, probation officers, and mental health workers and (2) research staff will use medical records and coordinate with the participants' healthcare providers, whenever possible, to track participants over time and to assist with scheduling follow-up assessments. If participants cannot be contacted, family, friends, clergy, probation officers, and mental health workers will be contacted by research staff with permission from the participants. The DSMB may recommend stopping the study if there is:

1. Early demonstration of effectiveness (assessed through the group sequential design) for suicide attempts. Effectiveness boundaries have been fully specified in Appendix C.
2. Early demonstration of lack of effectiveness for Aviva (assessed by specifying the group sequential design with binding futility). Futility boundaries have been fully specified in Appendix C.
3. Determination of unexpected, significant or unacceptable risk to participants by the monitoring committee. Based on historical prevalence of suicide attempts in this target population, we anticipate that up to half of enrolled patients could attempt suicide during a 104 week follow-up assessment period. DSMB will review all suicide attempts for causality to the investigational device, and if there are more SAs in the treatment arm than control arm, the study enrollment will be paused until DSMB can review under this stopping rule.

12. APPROACH TO ENSURE INTEGRITY OF DATA TO SUPPORT PRIMARY ENDPOINTS

12.1 REDUCE LIKELIHOOD OF PLACEBO EFFECT

- During the consent process, participants will be informed that they will be assigned to "one of two digital interventions." Participants will be informed that both interventions include some combination of supportive listening, education about crisis services, software services and referrals to treatment services. Thus, participants in both conditions will have the expectation that they are participating in a potentially efficacious intervention.
- Participants will be randomized to treatment and control conditions. We will maintain blinding of the participant to treatment through the use of a sham app. The app will be robust as it will include the same number of weekly interactions with subjects(frequency

and duration) as the treatment. The sham app content will include digitized versions of patient information that is typically distributed at discharge.

- We will conduct a blinding assessment. At 104 weeks, we will ask the participant: The blinding index (BI) will be calculated by asking participants: “Do you think you received a suicide prevention app? Why?: (1) Yes (2) No (3) I don’t know”.

12.2 REDUCE LIKELIHOOD OF SELECTION BIAS IN RECRUITMENT AND ENROLLMENT

- Recruitment: There will be training sessions that explain the inclusion/exclusion criteria and importance of the study and randomization to the staff.
- Enrollment in treatment arms: As previously noted, we will reduce selection bias by using a randomization procedure that adjusts for factors that may affect outcomes. Our randomization will occur with allocation concealment.

12.3 REDUCE LIKELIHOOD OF INACCURATE OR BIASED PATIENT REPORTS DURING ASSESSMENTS

- Participants will be instructed to schedule their phone call during a time when they are free from distractions and can provide reliable and accurate responses.
- Data will be collected by research assessors. The Clinicians (PIs) may be responsible for patient care during study participation, so having independent research assessors helps mitigate the risk of potential bias

12.4 REDUCE LIKELIHOOD OF STAFF DEVIATING FROM THE DATA COLLECTION PROTOCOL

- The data collectors will be trained on conducting the interviews and recording data. The training will include role-playing and simulations. Training will cover how to respond to adverse events.
- The data safety and monitoring plan will be shared with the clinicians. For example, they will be trained on how to differentiate an active suicide crisis from a person who has suicide intent.
- Data collectors will receive ongoing supervision.
- The study team will make appointments for assessments when there will not be distractions for the participants.
- Data will be complete because our research assessors are asking participants the questions. Participants are not completing paper and pencil questionnaires, wherein they could accidentally skip responses.

13. DATA SECURITY FOR AVIVA

13.1 CYBERSECURITY AND DATA COLLECTION

Data will be collected on a platform built on the fundamental principle of anonymity with security and privacy engineered into the core design. This includes (1) applying a security framework, and (2) ensuring data segregation of Patient Health Information (PHI). The data will not retain participant names when entered into our system and will only be accessible by authorized users. Study measures to ensure cybersecurity and data protection include:

- Unique password-protected user accounts.
- Detailed user account enrollment. When users begin the enrollment process and have provided their access code, they will be prompted to fill in their identity information, including their name, date of birth, email address, etc., and a personal password to protect their account. The combination of both their enrolled phone number and personal password will allow them to access the application on a supported mobile device.

- Strong password requirements. User passwords will be required to have: 1) 8 or more characters in length; 2) a mixture of both uppercase and lowercase letters; 3) a mixture of letters and numbers; and 4) at least one special character (e.g., ! @ # ?).
- Security and penetration testing.

13.2 SECURITY CULTURE

The technology group's primary tool to ensure security for the users will be by starting with a security culture. All server-side processes will be run in docker containers with minimal privileges. Access to non-development environments will be restricted to a few employees and our policy will require it to be used only in emergency measures and with oversight.

13.3 COMMUNICATION

Connectivity to and within Aviva will be severely restricted, and all communication will be encrypted.

13.4 ROLE-BASED PRODUCTION ACCESS

Access across all deployment tiers (development, beta, production) leverages a role-based entitlement system that associates privileges with a certain role, which will be then associated with appropriate users to ensure comprehensive access management.

13.5 DATA ENCRYPTION

All personally identifiable information will be decrypted only in memory and encryption keys will provide a second check on access controls.

13.6 SECURITY CULTURE

All activity within, access of, and attempted access of Aviva will be monitored, logged, and stored for anomaly detection and auditing.

13.7 TESTING

Aviva will be declaratively provisioned and will have an extensive testing process run with automated scripts.

14. DIRECT ACCESS TO SOURCE DATA/DOCUMENTS

Direct access will be granted to authorized representatives from the sponsor, host institution and the regulatory authorities to permit trial-related monitoring, audits and inspections.

15. QUALITY CONTROL AND QUALITY ASSURANCE PROCEDURES

The study will be conducted in accordance with the current approved protocol, ICH GCP, relevant regulations and standard operating procedures. Regular monitoring will be performed according to ICH GCP. Data will be evaluated for compliance with the protocol and accuracy in relation to source documents. Following written standard operating procedures, the monitors will verify that the clinical trial is conducted, and the data generated, documented and reported in compliance with the protocol, GCP and applicable regulatory requirements.

16. ETHICS

16.1 DECLARATION OF HELSINKI

The Investigator will ensure that this study is conducted in accordance with the principles of the Declaration of Helsinki.

16.2 ICH GUIDELINES FOR GOOD CLINICAL PRACTICE

The Investigator will ensure that this study is conducted in full conformity with relevant regulations and with the ICH Guidelines for Good Clinical Practice (CPMP/ICH/135/95) July 1996.

16.3 APPROVALS

The protocol, informed consent form, participant information sheet and any proposed advertising material will be submitted to an appropriate Institutional Review Board (IRB), regulatory authorities, and host institution(s) for written approval. The Investigator will submit and, where necessary, obtain approval from the above parties for all substantial amendments to the original approved documents.

16.4 PARTICIPANT CONFIDENTIALITY

The trial staff will ensure that the participants' anonymity is maintained. The participants will be identified only by a participant's ID number on the eCRF and in any electronic database. All documents will be stored securely and only accessible by trial staff and authorized personnel. The study will comply with the Data Protection Act 1998 which requires data to be anonymized as soon as it is practical to do so.

16.5 OTHER ETHICAL CONSIDERATIONS

Notable concerns apply to the digital delivery of CBT that may not be as problematic in traditional face-to-face interventions. Primary concerns about the digital delivery of therapy surround confidentiality and the absence of a potentially vital ingredient: the human therapist.34,35,36,37 From an ethical standpoint, the convenience, accessibility and effectiveness of digital CBT must be balanced against the potential disadvantages and harms associated with deviating from more traditional face-to-face interventions.

17. DATA HANDLING AND RECORD KEEPING

All study data will be entered on a HIPAA compliant software. ICH GCP requires that electronic data entry systems are validated, and that Standard Operating Procedures are maintained. The participants will be identified by a study specific participants number and/or code in any database. The name and any other identifying detail will NOT be included in any study data electronic file.

18. FINANCING AND INSURANCE

The funding for this study comes from seed/venture capital raised by the sponsor. The sponsor has insurance arrangements and product liability in place.

19. PUBLICATION POLICY

Publication of study results is governed by the clinical trial agreement (CTA) with each study site.

20. REFERENCES

American Psychiatric Association (2000). Diagnostic and statistical manual of mental disorders. 4th ed. text revision. Washington, DC: American Psychiatric Association. 2000.

Bang, H., Flaherty, S. P., Kolahi, J., & Park, J. (2010). Blinding assessment in clinical trials: a review of statistical methods and a proposal of blinding assessment protocol. Clinical Research and Regulatory Affairs, 27(2), 42-51.

Beck, A. T., Kovacs, M., & Weissman, A. (1979). Assessment of suicidal intention: The Scale for Suicide Ideation. Journal of Consulting and Clinical Psychology, 47, 343–352. doi:10.1037//0022-006x.47.2.343

Beck, A. T., & Steer, R. A. (1993). Beck Scale for Suicide Ideation manual. San Antonio, TX: Psychological Corporation.

Beck A.T., Steer R.A. (1988) Manual for the Beck Hopelessness Scale. San Antonio, TX: Psychological Corporation.

Beck, A. T., Steer, R. A., Brown, G. K. (1996). The Beck Depression Inventory, 2nd ed. San Antonio, TX: Psychological Corporation.

Beck, A. T., Weissman, A., Lester, D., & Trexler, L. (1974). The measurement of pessimism: The Hopelessness Scale. Journal of Consulting and Clinical Psychology, 42, 861–865.

Betz, M. E., Arias, S. A., Miller, M., Barber, C., Espinola, J. A., Sullivan, A. F., Manton, A. P., Boudreaux, E. D. (2015). Change in Emergency Department Providers' Beliefs and Practices After Use of New Protocols for Suicidal Patients. Psychiatric Services, 66, 6, 625-631. https://www.ncbi.nlm.nih.gov/pubmed/25726978

Brown, G. K, Have, T. T., Henriques, G. R., Xie, S. X., Hollander, J. E., & Beck, A. T. (2005). Cognitive therapy for the prevention of suicide attempts: A randomized controlled trial. Journal of the American Medical Association, 294, 563-570. https://www.ncbi.nlm.nih.gov/pubmed/16077050

Bryan, C. J., Kanzler, K. E., Grieser, E., Martinez, A., Allison, S., & McGeary, D. (2017). A Shortened Version of the Suicide Cognitions Scale for Identifying Chronic Pain Patients at Risk for Suicide. Pain Practice : the Official Journal of World Institute of Pain, 17, 3, 371-381. https://www.ncbi.nlm.nih.gov/pubmed/27317370

Bryan, C.J., Andreski, S.R., McNaughton-Cassill, M., & Osman, A. (2014). Agency is associated with decreased emotional distress and suicidal ideation in military personnel. Archives of Suicide Research, 18, 241-250. https://www.ncbi.nlm.nih.gov/pubmed/24712868

Bryan, C.J., Mintz, J., Clemans, T.A., Leeson, B., Burch, T.S., Williams, S.R., Maney, E., & Rudd, M.D. (2017). Effect of crisis response planning vs. contracts for safety on suicide risk in U.S. Army Soldiers: a randomized clinical trial. Journal of Affective Disorders, 212, 64-72. https://www.ncbi.nlm.nih.gov/pubmed/28142085

Bryan, C.J., Morrow, C.E., Etienne, N., & Ray-Sannerud, B. (2013). Guilt, Shame, and suicidal ideation in a military outpatient clinical sample. Depression and Anxiety, 30, 55-60. https://www.ncbi.nlm.nih.gov/pubmed/23077111

Bryan, C.J., Oakey, D.N., & Harris, J.A. (2018). Reasons for living among U.S. Army personnel thinking about suicide. Cognitive Therapy and Research, 42, 758-768. https://www.semanticscholar.org/paper/Reasons-for-Living-Among-U.S.-Army-Personnel-About-Bryan-Oakey/3ce727487a1dee817306916bb3219f9f002d2a5

Carter, G. L., Clover, K., Whyte, I. M., Dawson, A. H., & D, E. C. (2007). Postcards from the EDge: 24- month outcomes of a randomised controlled trial for hospital-treated self-poisoning. The British Journal of Psychiatry, 548-553. https://www.ncbi.nlm.nih.gov/pubmed/18055960

Carter, G. L., Clover, K., Whyte, I. M., Dawson, A. H., & D'Este, C. (2013). Postcards from the EDge: 5-year outcomes of a randomised controlled trial for hospital-treated self-poisoning. British Journal of Psychiatry, 202, 5, 372-380. https://www.ncbi.nlm.nih.gov/pubmed/23520223

Centers for Disease Control and Prevention. (2018). Vital Signs: Trends in State Suicide Rates. https://www.cdc.gov/vitalsigns/suicide/index.html

Centers for Disease Control and Prevention. (2020). Web-based Injury Statistics Query and Reporting System (WISQARS). National Center for Injury Prevention and Control. www.cdc.gov/injury/wisqars/index.html.

Centers for Disease Control and Prevention, National Center for Health Statistics. (2020). 1999-2018 Wide Ranging Online Data for Epidemiological Research (WONDER), Multiple Cause of Death files [Data file]. Retrieved from http://wonder.cdc.gov/ucd-icd10.html

Comtois, K. A., Kerbrat, A. H., DeCou, C. R., Atkins, D. C., Majeres, J. J., Baker, J. C., & Ries, R. K. (2019). Effect of augmenting standard care for military personnel with brief caring text messages for suicide prevention: a randomized clinical trial. JAMA psychiatry, 76(5), 474-483.

Food and Drug Administration. (2012). Guidance for industry: suicidal ideation and behavior: prospective assessment of occurrence in clinical trials. Rockville, MD.

Friedman, L. M., Furberg, C. D., DeMets, D. L., Reboussin, D. M., & Granger, C. B. (2015). Fundamentals of clinical trials. springer.

Ghahramanlou-Holloway, M., Neely, L. L., Tucker, J., Caffery, K., Colborn, V., & Koltko, V. (2015). Inpatient Cognitive Behavior Therapy Approaches for Suicide Prevention. Current Treatment Options in Psychiatry, 2, 4, 371-382. https://link.springer.com/article/10.1007/s40501-015-0063-4

Gomes-Oliveira MH, Gorenstein C, Neto FL, Andrade LH, & Wang YP. (2012). Validation of the Brazilian Portuguese version of the Beck Depression Inventory-II in a community sample. Revista Brasileira de Psiquiatria. 34: 389–394. pmid:23429809

Grothe KB, Dutton GR, Jones GN, Bodenlos J, Ancona M, & Brantley PJ. (2005). Validation of the Beck Depression Inventory-II in a low-income African American sample of medical outpatients. Psychological Assessment. 17: 110–114. pmid:15769232

International Organization for Standardization. (2011). ISO 14155: 2011 Clinical investigation of medical devices for human subjects–Good clinical practice.

Guy W. ECDEU Assessment Manual for Psychopharmacology. (1976). Rockville, MD: U.S. Department of Health, Education, and Welfare.

Gysin-Maillart, A., Schwab, S., Soravia, L., Megert, M., Michel, K., & Tsai, A. C. (2016). A Novel Brief Therapy for Patients Who Attempt Suicide: A 24-months Follow-Up Randomized Controlled Study of the Attempted Suicide Short Intervention Program (ASSIP). Plos Medicine, 13, 3. https://www.ncbi.nlm.nih.gov/pubmed/26930055

Heisel, M. J., & Flett, G. L. (2006). The development and initial validation of the Geriatric Suicide Ideation Scale (GSIS). The American Journal of Geriatric Psychiatry, 14, 742–751. doi:10.1097/01.JGP.0000218699.27899.f9

Katz, R., Katz, J., & Shaw, B. F. (1999). Beck Depression Inventory and Hopelessness Scale. In M. E. Maruish (Ed.), The use of psychological testing for treatment planning and outcomes assessment (2nd ed.; pp. 921–933). Mahwah, NJ: Erlbaum.

Knesper D. J., American Association of Suicidology, & Suicide Prevention Resource Center. (2011). Continuity of care for suicide prevention and research: Suicide attempts and suicide deaths subsequent to discharge from the Emergency Department of Psychiatry Inpatient Unit. Newton, MA: Education Development Center, Inc. http://www.sprc.org/sites/default/files/migrate/library/continuityofcare.pdf

Kojima M, Furukawa TA, Takahashi H, Kawai M, Nagaya T, & Tokudome S. (2002). Cross-cultural validation of the Beck Depression Inventory-II in Japan. Psychiatry Research. 110(3): 291–299. pmid:12127479

Linehan, M. M., Comtois, K. A., Brown, M. Z., Heard, H. L., & Wagner, A. (2006). Suicide Attempt Self-Injury Interview (SASII): development, reliability, and validity of a scale to assess suicide attempts and intentional self-injury. Psychological assessment, 18(3), 303.

Lippert, S. C., Nesper, A., Jain, N., Fahimi, J., Pirrotta, E., & Wang, N. E. (2016). 142 Mental Health Emergency Department Visits: 24 Hours and Counting, Characteristics Associated with Prolonged Length of Stay. Annals of Emergency Medicine, 68, 4. https://www.annemergmed.com/article/S0196-0644(16)30612-6/fulltext

Miller, I. W., Camargo, C. A. J., Arias, S. A., Sullivan, A. F., Allen, M. H., Goldstein, A. B., Manton, A. P. (2017). Suicide Prevention in an Emergency Department Population: The ED-SAFE Study. Jama Psychiatry, 74, 6, 563-570. https://www.ncbi.nlm.nih.gov/pubmed/28456130

Motto, J. A., & Bostrom, A. G. (2001). A Randomized Controlled Trial of Postcrisis Suicide Prevention. Psychiatric Services, 52, 6, 828-833. https://www.ncbi.nlm.nih.gov/pubmed/11376235

National Action Alliance for Suicide Prevention: Transforming Health Systems Initiative Work Group. (2018). Recommended standard care for people with suicide risk: Making health care suicide safe. Washington, DC: Education Development Center, Inc.

National Institutes of Health. (2018). National Cancer Institute. Common Terminology Criteria for Adverse Events (CTCAE), Version 5.0, November 2017. https://ctep.cancer.gov/protocoldevelopment/electronic_applications/docs/CTCAE_v5_Quick_Reference_8.5x11.pdf

Nock, M. K., Hwang, I., Sampson, N. A., & Kessler, R. C. (2010). Mental disorders, comorbidity and suicidal behavior: Results from the National Comorbidity Survey Replication. Molecular Psychiatry, 15, 8, 868-876. https://www.ncbi.nlm.nih.gov/pmc/articles/PMC2889009/

Nock, M., Park, J., Finn, C., Deliberto, T., Dour, H., & Banaji, M. (2010). Measuring the Suicidal Mind: Implicit Cognition Predicts Suicidal Behavior. Psychological Science, 21, 4, 511-517. https://www.ncbi.nlm.nih.gov/pubmed/20424092

nQuery8 – Power and Sample Size for Group Sequential Trials, Ver. 8.5.2.0 (2020), Statistical Solutions Ltd., Cork, Ireland, www.statsols.com

Olfson, M., Wall, M., Wang, S., Liu, S.-M., Crystal, S., Gerhard, T., & Blanco, C. (2016). Short-Term suicide risk after psychiatric hospital discharge. JAMA Psychiatry, 73, 11, 1119-1126. https://www.ncbi.nlm.nih.gov/pubmed/27654151

Osman A, Barrios F, Gutierrez P, Williams J, Bailey J. (2008). Psychometric properties of the Beck Depression Inventory-II in nonclinical adolescent samples. Journal Clinical Psychology. 64(1): 83–102. pmid:18161034

Park, J., Bang, H., & Cañette, I. (2008). Blinding in clinical trials, time to do it better. Complementary Therapies in Medicine, 3(16), 121-123.

PASS 2019 Power Analysis and Sample Size Software (2019). NCSS, LLC. Kaysville, Utah, USA, ncss.com/software/pass.

Pocock, S. J., Clayton, T. C., & Stone, G. W. (2015). Challenging issues in clinical trial design: part 4 of a 4-part series on statistics for clinical trials. Journal of the american college of cardiology, 66(25), 2886-2898.

Posner, K., Brown, G.K., Stanley, B. (2011). The Columbia-Suicide Severity Rating Scale: initial validity and internal consistency findings from three multisite studies with adolescents and adults. Am J Psychiatry.;168(12):1266–1277. https://www.ncbi.nlm.nih.gov/pubmed/22193671

Rudd, M. D. (2000). The suicidal mode: a cognitive-behavioral model of suicidality. Suicide and Life-Threatening Behavior, 30(1), 18-33.

Rudd, M. D., Bryan, C. J., Wertenberger, E. G., Peterson, A. L., Young-McCaughan, S., Mintz, J., Williams, S. R., Bruce, T. O. (2015). Brief cognitive-behavioral therapy effects on post-treatment suicide attempts in a military sample: results of a randomized clinical trial with 2-year follow-up. The American Journal of Psychiatry, 172, 5, 441-9. 7. https://www.ncbi.nlm.nih.gov/pubmed/25677353

Segal DL, Coolidge FL, Cahill BS, & O'Riley AA. (2008). Psychometric properties of the Beck Depression Inventory—II (BDI-II) among community-dwelling older adults. Behavior Modification. 32: 3–20. pmid:18096969

Shepard, D. S., Lwin, A. K., Gurewich, D., Reed, G. A., & Silverman, M. M. (2015). Suicide and Suicidal Attempts in the United States: Costs and Policy Implications. Suicide and Life-Threatening Behavior, 46 (3), 352-62. https://www.ncbi.nlm.nih.gov/pubmed/26511788

Stanley, B., Brown, G. K., Brenner, L. A., Galfalvy, H. C., Currier, G. W., Knox, K. L., Chaudhury, S. R., & Green, K. L. (2018). Comparison of the Safety Planning Intervention With Follow-up vs Usual Care of Suicidal Patients Treated in the Emergency Department. Jama Psychiatry. https://www.ncbi.nlm.nih.gov/pubmed/29998307

Subica A, Fowler C, Elhai J, Frueh C, Sharp C, Kelly E, et al. (2014). Factor structure and diagnostic validity of the Beck Depression Inventory-II with adult clinical inpatients: Comparison to a gold-standard diagnostic interview. Psychological Assessment. 26(4): 1106–1115. pmid:24932646

Substance Abuse and Mental Health Services Administration. (2017). Suicide prevention. https://www.samhsa.gov/find-help/suicide-prevention

Tarrier, N., Taylor, K., & Gooding, P. (2008). Cognitive-behavioral interventions to reduce suicide behavior: A systematic review and meta-analysis. Behavior Modification, 32, 77–108.

The Joint Commission. (2016). Sentinel event alert: Detecting and treating suicide ideation in all settings. Retrieved from https://www.jointcommission.org/assets/1/18/SEA_56_Suicide.pdf

The Zero Suicide Initiative. (2018). The National Action Alliance for Suicide Prevention and the Suicide Prevention Resource Center. http://zerosuicide.sprc.org

Ting, N. (2018). Statistical interactions in a clinical trial. Therapeutic innovation & regulatory science, 52(1), 14-21.

Togo, K., & Iwasaki, M. (2013). Optimal timing for interim analyses in clinical trials. Journal of biopharmaceutical statistics, 23(5), 1067-1080.

U.S. Department of Veterans Affairs. (2008). Safety plan treatment manual to reduce suicide risk: Veteran version. Retrieved from https://www.mentalhealth.va.gov/docs/va_safety_planning_manual.pdf

US Food and Drug Administration. (2012). Guidance for industry: Suicidal ideation and behavior: Prospective assessment of occurrence in clinical trials. Silver Spring, MD: US Department of Health and Human Services.

Wang Y, Gorenstein C. (2013). Psychometric properties of the Beck depression inventory-II: a comprehensive review. Revista Brasileira de Psiquiatria. 35(4): 416–431. pmid:24402217

ATTACHMENT A

TITLE: Formal Statistical Hypotheses and Mathematical Forms

- Null Hypothesis: HR = 1.
- Alternative Hypothesis: HR < 1.
- Where HR = Hazard Ratio

By way of background, here is the supportive information from the protocol (see Attachment 0601 IDE Clinical Protocol, page 218):

- Design a group sequential test with four Looks when 60%, 75%, 90% and 100% of the statistical information has been obtained.
- Assume exponential distributions for Group 1 Sham and Group 2 Aviva time to first SA after randomization.
  Set overall Type I error rate to 0.025 for the one-sided alternative hypothesis.
  Set the power to 90% when HR = 0.3826 as reported in Rudd (2015).
- Set the total study time to 24 months.
  Set the accrual time to 12 months.
  Assume uniform accrual of patients over 12 months.
  Assume equal randomization to the Sham and Aviva groups.
  Determine the stopping boundaries for the GST for efficacy and for non-binding futility.
  Determine the number of events needed over time for statistical significance at the end of the study and estimate the sample size in each group when testing the null hypothesis with the log-rank test. N has increased from 374 to 391 because of using the non binding futility instead of binding futility

ATTACHMENT B

TITLE: Overview of Aviva Product Development

Given the lack of available FDA cleared and approved products for adults who do not have Schizophrenia and Schizoaffective Disorder, the scientists who developed BCBT/CT-SP (the only proven protocols for reducing suicide attempts in adults at risk) created Aviva. Thus, Aviva has the same theoretical basis as the face-to-face, suicide-specific CBT protocols. As such, sessions and practice exercises are guided by the new understanding that suicidal ideation and attempts emerge from deficits in cognitive, behavioral, emotional, and physical domains. Aviva is designed to reduce attempts regardless of psychiatric diagnosis. The ability to address suicide risk in the absence of other psychiatric diagnoses is significant, as it is estimated that approximately 10-40% of those at risk do not have another mental health diagnosis such as Major Depressive Disorder or Schizophrenia (Asnis et al., 1993). Below is a description of the cognitive, behavioral, emotional, and physical risk factors.

- Cognitive risk factors for suicide include negative self-perceptions, low cognitive flexibility (which impairs executive functioning), problem-solving deficits, and a pessimistic style of thinking that underestimates the likelihood of positive outcomes.
- Behavioral risk factors for suicide include deficits in distress tolerance, emotion regulation, and interpersonal communication.
- Emotional risk factors for suicide include psychiatric disorders and emotional liability.
- Physical risk factors include vulnerabilities at the genetic and biological levels and medical conditions.

These deficits result in an inability to regulate distressing thoughts and emotions when presented with a stressor (Bryan & Rudd, 2018). Typical CBT protocols do not focus on these areas in a manner that is suicide specific. Thus, to prevent suicidal ideation and attempts, the precise interventions included in Aviva specifically target cognitive, behavioral, emotional, and physical domains with a focus on suicide risk and teaches patients how to:

- Be able to understand their risk factors and identify warning signs of impending suicide risk;
- Know what to do when their suicide risk is increasing; and
- Develop skills to prevent suicide risk from increasing and from emerging.

Table B1 shows the topics discussed in Aviva sessions. These topics are all from the original face-to-face protocols. Consistent with the theoretical foundation of the face-to-face protocols, Aviva targets the cognitive (8 sessions), behavioral (6 sessions), emotional (5 sessions), and physical (5 sessions) domains that contribute to suicide attempts (Note, a session topic may target multiple domains).

To build Aviva, Oui incorporated into the app the important elements for effective suicide prevention (Bryan et al., 2018): a useful theoretical model, a manualized protocol that can be delivered with fidelity, emphasis on patient adherence, skills training, respect for patient autonomy, a plan for staying safe and an individual therapy format. Table B1 shows how the procedures and content of Aviva are consistent with the important elements of the BCBT/CT-SP protocols.

Table B1. Aviva sessions and domains targeted by each session.

Session Topic	Cognition	Behavior	Emotion	Physical	Clinician Assisted
Onboarding		X			X
Reasons for Living / Hope kit	X		X		
Get going (Activity Planning/Behavior Activation)		X			
Relaxation and Mindfulness		X	X	X	
Improve Sleep (Sleep Tracking)				X	
Spot it (ABC Exercise)	X				
Test it (Challenging Questions)	X				
Switch it (Patterns of Problematic Thinking)	X				
Coping Cards	X	X			
Review Phase 1 (Relapse Prevention)	X	X	X	X	
Review Phase 2 (Relapse Prevention)	X	X	X	X	
What’s Next? (Termination Planning)	X	X	X	X	

Table B2. Elements of Effective Suicide Prevention: How Aviva has digitized procedures and content from proven cognitive-behavioral protocols (Brown et al., 2005, Rudd et al., 2015, Bryan et al. 2017) to allow scalability and access to effective suicide prevention per Joint Commission, Department of Veteran Affairs, and Department of Defense guidelines.

Elements of Effective Suicide Prevention	Brief Cognitive-Behavioral Therapy for Suicide Prevention and Cognitive therapy for suicide prevention (BCBT/CT-SP)	Aviva
Clinically Useful Theoretical Model	Clinicians deliver BCBT/CT-SP after a theoretically informed assessment has deemed that the patient is appropriate for treatment.	Clinicians provide a link and an access code after a theoretically informed assessment has deemed that the patient is appropriate for treatment.
	Discussions are based on the understanding of the etiology of suicidal ideation and suicide attempts.	Interactive features (e.g., chat bot messaging, video, audio, and quizzes) present content that is based on the understanding of the etiology of suicidal ideation and suicide attempts.
Manualized Protocol that is Specific to Suicide Prevention	Clinicians deliver BCBT/CT-SP's time-limited, specialized protocol. Additionally, BCBT/CT-SP consists of standardized psychoeducational material about suicidal ideation and attempts.	Aviva delivers time-limited, specialized and structured modular learning experiences designed around BCBT/CT-SP manualized protocol. Additionally, Aviva consists of tutorial videos that deliver standardized psychoeducational material about suicidal ideation and attempts.
Patient Adherence	Clinicians ask patients to agree in writing to participate in the intervention fully. Additionally, clinicians regularly check in with patients about their progress.	Aviva asks patients to agree in writing to participate in the intervention fully. Additionally, Aviva follows-up with patients about their progress via smartphone notifications, text messages, emails, and/or in-app messages.
Skills-Training Focus	Clinicians engage in conversations that teach BCBT/CT-SP skills and allow patients to apply the skills to their experiences.	Aviva's chat feature supports conversations that teach BCBT/CT-SP skills and allow patients to apply the skills to their experiences.
	Patients create a paper log to document "lessons learned."	Patients create a digital treatment log to document "lessons learned." The log contains short summaries of critical lessons from Aviva sessions to help patients remember new material.
	Patients are instructed to create paper cards that help them cope with challenging situations. The cards contain short summaries of critical	Patients are instructed to create digital cards that help them cope with challenging situations.

Elements of Effective Suicide Prevention	Brief Cognitive-Behavioral Therapy for Suicide Prevention and Cognitive therapy for suicide prevention (BCBT/CT-SP)	Aviva
	lessons from BCBT/CT-SP sessions to help patients remember new material.	
	Clinicians describe stories about how others process key principles and apply the skills.	Aviva uses videos to show how individuals with similar conditions (portrayed by actors) process key principles and apply the skills.
Respect Patient Autonomy	Clinicians invite patients to participate in the planning of their care and the development of plans to prevent a suicidal crisis.	Aviva invites patients to participate in the planning of their care by providing practice recommendations that are based on the patient’s stated preferences, and stated confidence with a particular skill.<br><br>Patients participate in the development of plans to prevent a suicidal crisis.
Plan for Staying Safe	Clinicians help patients create a customized plan to avoid a crisis. The plan includes things they can do to avoid a crisis, friends/family they can contact, and professionals they can contact.	Aviva helps patients create a customized plan to avoid a crisis. The plan includes things they can do to avoid a crisis, friends/family they can contact, and professionals they can contact.
Individual Therapy Format	Clinicians deliver BCBT/CT-SP in an individual therapy format.	Aviva delivers BCBT/CT-SP in an individual therapy format.

ATTACHMENT C

TITLE: Power Calculation and Group Sequential Design for Aviva Trial – Suicide Attempts (Mathematical Formulations) – Non-Binding Futility

The primary effectiveness variable is the time to first suicide attempt (SA) measured from randomization in days. The hazard ratio (HR) reported by Rudd (2015) was 0.3826. Survival curves were estimated using the plot in his paper and the HR of 0.3826. The same HR was taken as the measure of success in the planned trial. The overall study would last up to 2 years (24 months) and accrual would take 12 months. Multiple scenarios were considered and a group sequential test (GST) with a total of 4 Looks as utilized in similar trials was adopted. Sample size calculations and operating characteristics were derived from nQuery (2021)[1]. It was agreed that power would be 90% to detect such an HR. Here are the study specifications in the following two tables. Note that the first table includes the sample size estimate and the estimated number of events or patients with a first suicide attempt (SA) post randomization.

STT15-2 / GST Survival with Accrual

	1
Test Significance Level, α	0.0250
1 or 2 Sided Test?	1
Accrual Period, a	12.0000
Maximum Follow-up Period, f	24.0000
Group 1 Exponential Parameter, λ_1	0.0115
Group 2 Exponential Parameter, λ_2	0.0044
Hazard Ratio, $h = \lambda_2/\lambda_1$	0.3826
Sample Size Ratio, n_2/n_1	**1.0051**
Group 1 Sample Size, n_1	**195**
Group 2 Sample Size, n_2	**196**
Power (%)	**90**
Events Required in Group 1, e_1	37
Events Required in Group 2, e_2	15
Looks Side Table Name	Looks-1

The second table of specifications is used to derive the group sequential test that is reported in the third table.

[1] nQuery 8 – Power and Sample Size for Group Sequential Trials, Version 8.7.2.0, Statistical Solutions Ltd., Cork, Ireland, 2021.

Looks-1

Interim Monitoring & Sample Size Re-Estimation

GST Parameters	
**Number of Looks**	4
**Information Times**	User Input
**Max Times**	1.0000
**Efficacy Bounds**	Spending Function
**Alpha Spending Function**	O'Brien-Fleming
**Power/HSD Parameter**	
**Truncate Bounds**	No
**Truncate At**	
**Futility Boundaries**	Non-Binding
**Beta Spending Function**	O'Brien-Fleming
**Power/HSD Parameter**	

The combination of the two sets of specifications resulted in the following table:

	1	2	3	4
**Information Time**	0.6000	0.7500	0.9000	1.0000
**Events**	31.2000	39.0000	46.8000	52.0000
**Lower Efficacy Bound**	-8.0000	-8.0000	-8.0000	-8.0000
**Upper Efficacy Bound**	2.6686	2.3865	2.1685	2.0748
**Futility bound**	0.8347	1.3133	1.7538	2.0748
**Nominal alpha**	0.0038	0.0085	0.0151	0.0190
**Incremental alpha**	0.0038	0.0058	0.0085	0.0069
**Cumulative alpha**	0.0038	0.0096	0.0181	0.0250
**Exit probability under H_1**	53.1685	25.6796	15.9430	5.2089
**Cumulative exit probability under H_1**	53.1685	78.8481	94.7911	100.0000
**Nominal beta**	0.2019	0.0945	0.0397	0.0190
**Incremental beta**	0.0337	0.0238	0.0254	0.0171
**Cumulative beta**	0.0337	0.0575	0.0829	0.1000
**Exit probability under H_0**	80.1869	12.1123	5.9180	1.7829
**Cumulative exit probability under H_0**	80.1869	92.2992	98.2171	100.0000

The null hypothesis is that the hazard ratio (HR) is 1, that is that the ratio of medians is 1 for assumed exponential distributions. A one-sided Log-Rank test with a group 1 (Sham) sample size of 195 and a group 2 (Aviva) sample size of 196 would achieve 90% power at a 0.025 significance

level to detect a hazard ratio of 0.3826 when the group 1 exponential hazard rate is 0.0115 (this gives a group 2 exponential hazard rate of 0.0044). Under these assumptions, the number of events will be 37 and 15 in group 1 and group 2 respectively. The maximum follow-up time for a subject is 24 time units (months) with an accrual period of 12 time units.

These results assume that the group sequential design has 3 interim sequential tests (4 total looks including final analysis). The O'Brien-Fleming spending function is used to determine the effectiveness test boundary. The O'Brien-Fleming spending function is used to determine the non-binding futility boundary.

The drift parameter for this design equals 3.4387. The drift parameter equals the square root of the maximum information level by the log hazard ratio. The ratio of this drift parameter to the drift parameter from the equivalent fixed term design represents the ratio of the sample sizes required for this GST design to that of its fixed term equivalent.

GST Boundaries (Z scale):

Upper effectiveness bounds: 2.6686, 2.3865, 2.1685, 2.0748
Futility bounds: 0.8347, 1.3133, 1.7538, 2.0748

ATTACHMENT D

TITLE: Mathematical Formulations for Suicidal Ideation

PASS 2020, v20.0.3   9/10/2020 2:59:16 PM 1

Two-Sample T-Tests Assuming Equal Variance

Numeric Results for an Equal-Variance T-Test
δ = μ1 - μ2
Hypotheses: H0: δ = 0 vs. H1: δ ≠ 0

Power	N1	N2	N	δ	σ	Alpha
0.83582	70	70	140	3.8	7.6	0.050
0.88160	80	80	160	3.8	7.6	0.050
0.91559	90	90	180	3.8	7.6	0.050
0.94043	100	100	200	3.8	7.6	0.050

References
Chow, S.C., Shao, J., Wang, H., and Lokhnygina, Y. 2018. Sample Size Calculations in Clinical Research, Third Edition.
Taylor & Francis/CRC. Boca Raton, Florida.
Julious, S. A. 2010. Sample Sizes for Clinical Trials. Chapman & Hall/CRC. Boca Raton, FL.
Machin, D., Campbell, M., Fayers, P., and Pinol, A. 1997. Sample Size Tables for Clinical Studies, 2nd Edition.
Blackwell Science. Malden, MA.
Zar, Jerrold H. 1984. Biostatistical Analysis (Second Edition). Prentice-Hall. Englewood Cliffs, New Jersey.

Report Definitions

- Power is the probability of rejecting a false null hypothesis.
- N1 and N2 are the number of items sampled from each population.
- N = N1 + N2 is the total sample size.
- μ1 and μ2 are the assumed population means.
- δ = μ1 - μ2 is the difference between population means at which power and sample size calculations are made.
- σ is the assumed population standard deviation for each of the two groups.
- Alpha is the probability of rejecting a true null hypothesis.

Summary Statements ———————————————

Group sample sizes of 70 and 70 achieve 83.582% power to reject the null hypothesis of equal means when the population mean difference is 3.8 (Aviva group mean change from baseline = 6.7 and Sham group mean change from baseline = 2.9) with a common standard deviation for both groups of 7.6 and with a significance level (alpha) of 0.050 using a two-sided two-sample equal-variance t-test. Even if the sample sizes are 80 and 80 and there are 20% dropouts in each group, the power is 88.16% when the same mean difference of 3.8 and standard deviation of 7.6 is observed. These are the values obtained from Bryan (2017). Therefore, the sample size required for the analyses involving suicide attempts (i.e., N= 391), exceeds the sample size required for the analysis involving suicide ideation (i.e., N = 140).

Dropout-Inflated Enrollment Sample Size			Sample Size				Expected Number of Dropouts		
Dropout Rate	N1	N2	N	N1'	N2'	N'	D1	D2	D
20%	70	70	140	88	88	176	18	18	36
20%	80	80	160	100	100	200	20	20	40
20%	90	90	180	113	113	226	23	23	46
20%	100	100	200	125	125	250	25	25	50

PASS 2020, v20.0.3 9/10/2020 2:59:16 PM 2

Two-Sample T-Tests Assuming Equal Variance

Definitions

- Dropout Rate (DR) is the percentage of subjects (or items) that are expected to be lost at random during the course of the study and for whom no response data will be collected (i.e. will be treated as "missing").
- N1, N2, and N are the evaluable sample sizes at which power is computed (as entered by the user). If N1 and N2 subjects are evaluated out of the N1' and N2' subjects that are enrolled in the study, the design will achieve the stated power.
- N1', N2', and N' are the number of subjects that should be enrolled in the study in order to end up with N1, N2, and N evaluable subjects, based on the assumed dropout rate. N1' and N2' are calculated by inflating N1 and N2 using the formulas N1' = N1 / (1 - DR) and N2' = N2 / (1 - DR), with N1' and N2' always rounded up. (See Julious, S.A. (2010) pages 52-53, or Chow, S.C., Shao, J., Wang, H., and Lokhnygina, Y. (2018) pages 32-

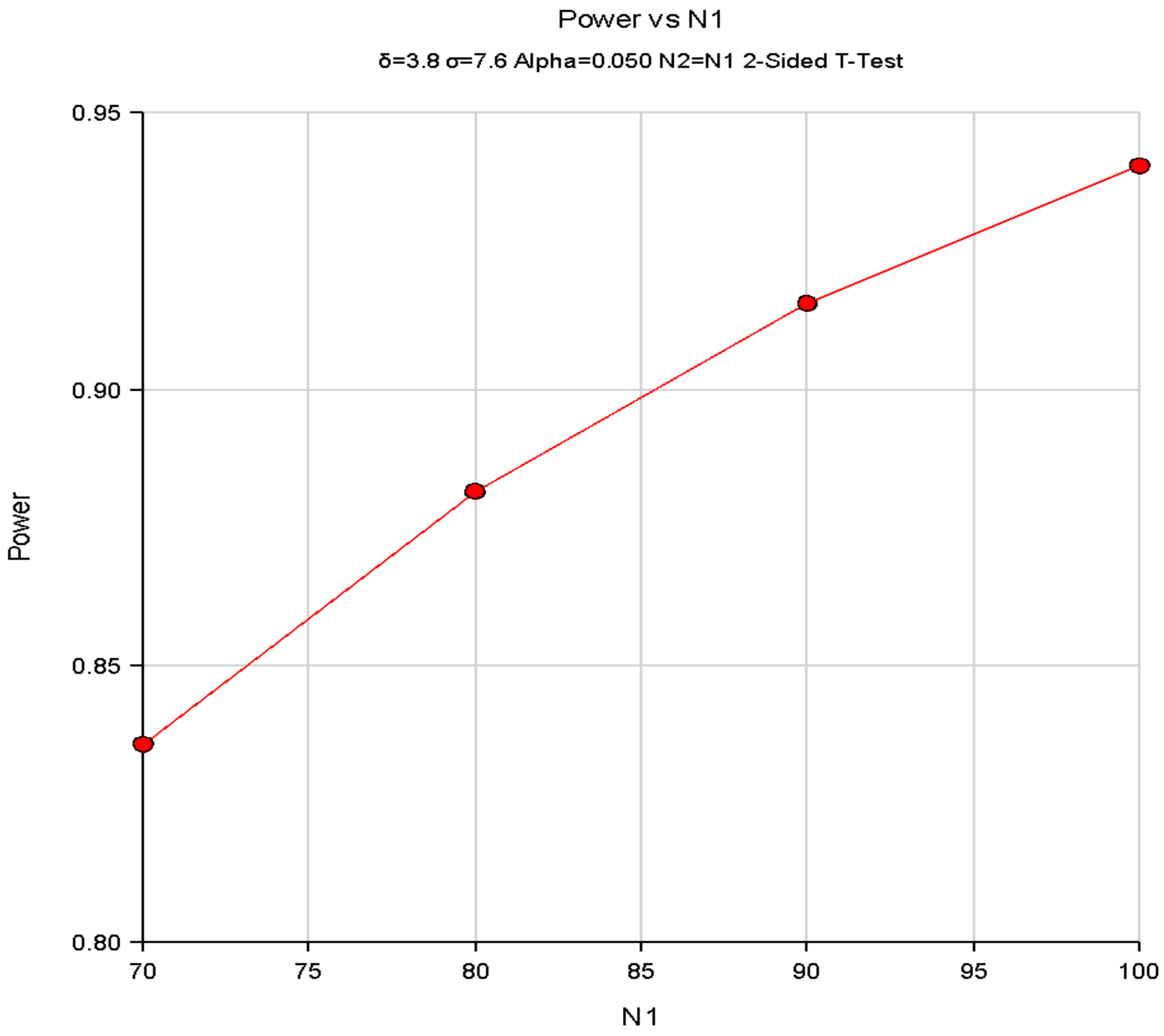

33.) D1, D2, and D are the expected number of dropouts. D1 = N1' - N1, D2 = N2' - N2, and D = D1 + D2.

Chart Section

PASS 2020, v20.0.3 9/10/2020 2:59:16 PM 3

Two-Sample T-Tests Assuming Equal Variance

Procedure Input Settings ————————————————

Autosaved Template File
C:\Users\tadarch\Documents\PASS 2020\Procedure Templates\Autosave\Two-Sample T-Tests Assuming Equal Variance - Autosaved 2020_9_10-14_59_17.t388

Design Tab

Solve For:	Power
Alternative Hypothesis:	Two-Sided
Alpha:	0.05
Group Allocation:	Equal (N1 = N2)
Sample Size Per Group:	70 to 100 by 10
Input Type:	Difference
δ:	3.8
σ:	7.6

- SAS code for MMRM #1 below; same for MMRM #2 based on completers only (with the same fixed value of overall baseline mean in LSMEANS)

```
PROC MIXED DATA=allpatients;
 CLASS visit patient; * visit refers to all scheduled post-baseline visits;
 MODEL cfb=base visit base*visit/DDFM=KR;
 REPEATED visit/SUBJECT=patient TYPE=UN;
 LSMEANS visit/AT basval=&basemn; * overall baseline mean (dropouts and completers
pooled);
```
