# Supplementary material for: A Digital Therapeutic Intervention for Inpatients With Elevated Suicide Risk: A Randomized Clinical Trial
Source: JAMA Netw Open. 2025 Aug 8;8(8):e2525809. doi: 10.1001/jamanetworkopen.2025.25809 (PMC12334960; doi:10.1001/jamanetworkopen.2025.25809)
Supplement: Supplement 2. — eMethods. Description of OTX-202, A Prescription Digital Therapeutic eTable 1. Site Characteristics eTable 2. Percentage of Patients in Each Treatment Group Who Completed Each Module eTable 3. Adverse Events (AEs) Reported by Enrolled Participants, Full Sample (n=331) eTable 4. Adverse Events (AEs) Reported by Participants Assigned to OTX-202 (n=164) eTable 5. Adverse Events (AEs) Reported by Participants Assigned to Control (n=167) [file jamanetwopen-e2525809-s002.pdf]

Supplemental Online Content

Bryan CJ, Simon P, Wilkinson ST, et al. A digital therapeutic intervention for inpatients with elevated suicide risk: a randomized clinical trial. *JAMA Netw Open*. 2025;8(8):e2525809. doi:10.1001/jamanetworkopen.2025.25809

eMethods. Description of OTX-202, A Prescription Digital Therapeutic
eTable 1. Site Characteristics
eTable 2. Percentage of Patients in Each Treatment Group Who Completed Each Module
eTable 3. Adverse Events (AEs) Reported by Enrolled Participants, Full Sample (n=331)
eTable 4. Adverse Events (AEs) Reported by Participants Assigned to OTX-202 (n=164)
eTable 5. Adverse Events (AEs) Reported by Participants Assigned to Control (n=167)

This supplemental material has been provided by the authors to give readers additional information about their work.

© 2025 Bryan CJ et al. *JAMA Network Open*

eMethods. Description of OTX-202, a prescription digital therapeutic

Theoretical and Scientific Foundation

The app's digitized suicide prevention protocol is based on the BCBT and CT-SP protocols, which together reduce suicide attempts by 40-76%.[1-6]

Content

OTX-202 includes 12 sessions, delivered in three phases:

a) Suicide-specific assessment and developing a plan to stay safe
b) Teaching suicide-specific cognitive-behavioral skills
c) Reinforcing suicide-specific skills to prevent relapse and suicide attempts (SAs)

Technology Format

OTX-202 will be deployed through smartphone devices on both the iOS and Android operating systems. A chatbot guides users through sessions. Explanatory animated videos explain new ideas to users and why they are important for reducing suicide risk. OTX-202 also includes clinical vignette videos, which complement the explanatory videos by having people discuss their personal experiences with each session. These clinical vignettes demonstrate the relevance and utility of the app to the user. Homework exercises from BCBT and CT-SP have been converted into interactive widgets that prompt practice. Example screenshots from the app are included below in the Figure.

Figure

Example screenshots from the OTX-202 smartphone app displaying (on the left) the menu of cognitive-behavioral skills (on the right) and an embedded tracking system to monitor skill use during the week.

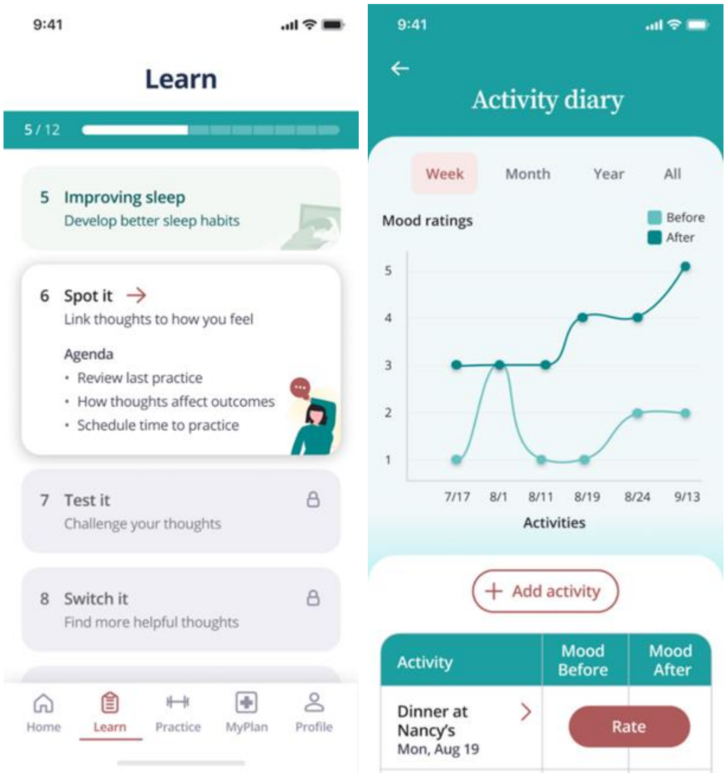

© 2025 Bryan CJ et al. *JAMA Network Open*

eTable 1. Site characteristics

	Site Characteristics n			Patient Ethnicity n (%)			Patient Race n (%)						
**Name**	**No. of Psych Beds**	**No. of Suicide-Related Admissions per Month**	**No. of Patients Enrolled**	**Hispanic**	**Non-Hispanic**	**Unknown**	**American Indian or Alaska Native**	**Asian**	**Black or African American**	**Native Hawaiian or Pacific Islander**	**White**	**Unknown or Not Reported**	**Other**
**The Ohio State University** Columbus, OH	100	55	91	8 (8.8)	82 (90.1)	1 (1.1)	0 (0)	3 (3.3)	10 (11.0)	1 (1.1)	70 (76.9)	0 (0)	7 (7.7)
**Yale University** New Haven, CT	76	10	62	12 (19.4)	50 (80.6)	0 (0)	1 (1.6)	5 (8.1)	4 (6.5)	0 (0)	49 (79.0)	1 (1.6)	2 (3.2)
**University of Colorado** Denver, CO	40	17.6	58	17 (29.3)	40 (69.0)	1 (1.7)	2 (3.4)	0(0)	5 (8.6)	0 (0)	45 (77.6)	3 (5.2)	3 (5.2)
**Northwell Health** New York City, NY	228	40	91	24 (26.4)	65 (71.4)	2 (2.2)	1 (1.1)	15 (16.5)	27 (29.7)	2 (2.2)	30 (33.0)	2 (2.2)	14 (15.4)
**University of Cincinnati** Cincinnati, OH	65	50	26	1 (3.8)	25 (96.2)	0 (0)	0 (0)	2 (7.7)	1 (3.8)	0 (0)	23 (88.5)	0 (0)	0 (0)
**CommonSpirit Health** Omaha, NE	84	400	11	3 (27.3)	8 (72.7)	0(0)	0 (0)	0 (0)	0 (0)	0 (0)	11 (100)	0 (0)	0 (0)

© 2025 Bryan CJ et al. *JAMA Network Open*

eTable 2. Number and percentage of patients in each treatment group completing each module across treatment groups

	OTX-202			Control	
	(n=168)			(n=171)	
	n	%		n	%
**Module 1**	149	(88.7)		156	(91.2)
**Module 2**	117	(69.6)		117	(68.4)
**Module 3**	98	(58.3)		105	(61.4)
**Module 4**	80	(47.6)		98	(57.3)
**Module 5**	69	(41.1)		85	(49.7)
**Module 6**	52	(31.0)		80	(46.8)
**Module 7**	40	(23.8)		72	(42.1)
**Module 8**	32	(19.0)		69	(40.4)
**Module 9**	29	(17.3)		66	(38.6)
**Module 10**	25	(14.9)		59	(34.5)
**Module 11**	23	(13.7)		55	(32.2)
**Module 12**	21	(12.5)		53	(31.0)

© 2025 Bryan CJ et al. *JAMA Network Open*

eTable 3. All adverse events (AEs) reported by enrolled participants, full sample (N=331)

		Related to Device? [a]			Severity [b]			Expected?	
**Adverse Event Type & Subtype**	**Total**	**Definitely No**	**Possibly**	**Definitely Yes**	**Mild**	**Moderate**	**Severe**	**No**	**Yes**
Total	398 (64.0%)	384 (62.2%)	8 (2.1%)	2 (0.6%)	215 (42.0%)	100 (23.3%)	76 (17.5%)	386 (63.7%)	12 (3.0%)
Blood and lymphatic system disorders	2 (0.6%)	2 (0.6%)	0 (0%)	0 (0%)	2 (0.6%)	0 (0%)	0 (0%)	2 (0.6%)	0 (0%)
Lymphadenopathy	1 (0.3%)	1 (0.3%)	0 (0%)	0 (0%)	1 (0.3%)	0 (0%)	0 (0%)	1 (0.3%)	0 (0%)
Thrombocytosis	1 (0.3%)	1 (0.3%)	0 (0%)	0 (0%)	1 (0.3%)	0 (0%)	0 (0%)	1 (0.3%)	0 (0%)
Cardiac disorders	4 (1.2%)	4 (1.2%)	0 (0%)	0 (0%)	3 (0.9%)	0 (0%)	1 (0.3%)	4 (1.2%)	0 (0%)
Chest pain	4 (1.2%)	4 (1.2%)	0 (0%)	0 (0%)	3 (0.9%)	0 (0%)	1 (0.3%)	4 (1.2%)	0 (0%)
Eye disorders	1 (0.3%)	1 (0.3%)	0 (0%)	0 (0%)	1 (0.3%)	0 (0%)	0 (0%)	1 (0.3%)	0 (0%)
Eye irritation	1 (0.3%)	1 (0.3%)	0 (0%)	0 (0%)	1 (0.3%)	0 (0%)	0 (0%)	1 (0.3%)	0 (0%)
Gastrointestinal disorders	16 (3.6%)	15 (3.3%)	0 (0%)	0 (0%)	9 (2.7%)	4 (1.2%)	2 (0.3%)	16 (3.6%)	0 (0%)
Abdominal pain	5 (1.5%)	4 (1.2%)	0 (0%)	0 (0%)	2 (0.6%)	2 (0.6%)	0 (0%)	5 (1.5%)	0 (0%)
Abdominal pain upper	2 (0.6%)	2 (0.6%)	0 (0%)	0 (0%)	1 (0.3%)	1 (0.3%)	0 (0%)	2 (0.6%)	0 (0%)
Cyclic vomiting syndrome	1 (0.3%)	1 (0.3%)	0 (0%)	0 (0%)	0 (0%)	0 (0%)	1 (0.3%)	1 (0.3%)	0 (0%)
Diarrhoea	1 (0.3%)	1 (0.3%)	0 (0%)	0 (0%)	0 (0%)	1 (0.3%)	0 (0%)	1 (0.3%)	0 (0%)
Gastric ulcer	1 (0.3%)	1 (0.3%)	0 (0%)	0 (0%)	1 (0.3%)	0 (0%)	0 (0%)	1 (0.3%)	0 (0%)
Irritable bowel syndrome	1 (0.3%)	1 (0.3%)	0 (0%)	0 (0%)	1 (0.3%)	0 (0%)	0 (0%)	1 (0.3%)	0 (0%)
Nausea	1 (0.3%)	1 (0.3%)	0 (0%)	0 (0%)	1 (0.3%)	0 (0%)	0 (0%)	1 (0.3%)	0 (0%)
Pancreatitis chronic	1 (0.3%)	1 (0.3%)	0 (0%)	0 (0%)	1 (0.3%)	0 (0%)	0 (0%)	1 (0.3%)	0 (0%)
Salivary hypersecretion	1 (0.3%)	1 (0.3%)	0 (0%)	0 (0%)	1 (0.3%)	0 (0%)	0 (0%)	1 (0.3%)	0 (0%)
Vomiting	2 (0.6%)	2 (0.6%)	0 (0%)	0 (0%)	1 (0.3%)	0 (0%)	1 (0.3%)	2 (0.6%)	0 (0%)
General disorders and administration site conditions	14 (3.6%)	14 (3.6%)	0 (0%)	0 (0%)	12 (3.0%)	1 (0.3%)	1 (0.3%)	14 (3.6%)	0 (0%)
Adverse drug reaction	8 (2.4%)	8 (2.4%)	0 (0%)	0 (0%)	7 (2.1%)	1 (0.3%)	0 (0%)	8 (2.4%)	0 (0%)

© 2025 Bryan CJ et al. *JAMA Network Open*

		Related to Device? [a]			Severity [b]			Expected?	
**Adverse Event Type & Subtype**	**Total**	**Definitely No**	**Possibly**	**Definitely Yes**	**Mild**	**Moderate**	**Severe**	**No**	**Yes**
Death	1 (0.3%)	1 (0.3%)	0 (0%)	0 (0%)	0 (0%)	0 (0%)	1 (0.3%)	1 (0.3%)	0 (0%)
Drug withdrawal syndrome	2 (0.6%)	2 (0.6%)	0 (0%)	0 (0%)	2 (0.6%)	0 (0%)	0 (0%)	2 (0.6%)	0 (0%)
Fatigue	1 (0.3%)	1 (0.3%)	0 (0%)	0 (0%)	1 (0.3%)	0 (0%)	0 (0%)	1 (0.3%)	0 (0%)
Inflammation	1 (0.3%)	1 (0.3%)	0 (0%)	0 (0%)	1 (0.3%)	0 (0%)	0 (0%)	1 (0.3%)	0 (0%)
Oedema	1 (0.3%)	1 (0.3%)	0 (0%)	0 (0%)	1 (0.3%)	0 (0%)	0 (0%)	1 (0.3%)	0 (0%)
Immune system disorders	4 (1.2%)	4 (1.2%)	0 (0%)	0 (0%)	2 (0.6%)	2 (0.6%)	0 (0%)	4 (1.2%)	0 (0%)
Asthma	1 (0.3%)	1 (0.3%)	0 (0%)	0 (0%)	0 (0%)	1 (0.3%)	0 (0%)	1 (0.3%)	0 (0%)
Drug hypersensitivity	1 (0.3%)	1 (0.3%)	0 (0%)	0 (0%)	1 (0.3%)	0 (0%)	0 (0%)	1 (0.3%)	0 (0%)
Hypersensitivity	2 (0.6%)	2 (0.6%)	0 (0%)	0 (0%)	1 (0.3%)	1 (0.3%)	0 (0%)	2 (0.6%)	0 (0%)
Infections and infestations	44 (11.2%)	44 (11.2%)	0 (0%)	0 (0%)	32 (8.2%)	10 (3.0%)	2 (0.6%)	44 (11.2%)	0 (0%)
Abscess	1 (0.3%)	1 (0.3%)	0 (0%)	0 (0%)	0 (0%)	1 (0.3%)	0 (0%)	1 (0.3%)	0 (0%)
Bacterial vaginosis	1 (0.3%)	1 (0.3%)	0 (0%)	0 (0%)	1 (0.3%)	0 (0%)	0 (0%)	1 (0.3%)	0 (0%)
Bronchitis	1 (0.3%)	1 (0.3%)	0 (0%)	0 (0%)	1 (0.3%)	0 (0%)	0 (0%)	1 (0.3%)	0 (0%)
COVID-19	12 (3.6%)	12 (3.6%)	0 (0%)	0 (0%)	9 (2.7%)	3 (0.9%)	0 (0%)	12 (3.6%)	0 (0%)
Gastroenteritis viral	1 (0.3%)	1 (0.3%)	0 (0%)	0 (0%)	0 (0%)	1 (0.3%)	0 (0%)	1 (0.3%)	0 (0%)
Herpes simplex	2 (0.6%)	2 (0.6%)	0 (0%)	0 (0%)	1 (0.3%)	1 (0.3%)	0 (0%)	2 (0.6%)	0 (0%)
Influenza	3 (0.9%)	3 (0.9%)	0 (0%)	0 (0%)	2 (0.6%)	0 (0%)	1 (0.3%)	3 (0.9%)	0 (0%)
Nasopharyngitis	5 (1.5%)	5 (1.5%)	0 (0%)	0 (0%)	5 (1.5%)	0 (0%)	0 (0%)	5 (1.5%)	0 (0%)
Norovirus infection	1 (0.3%)	1 (0.3%)	0 (0%)	0 (0%)	1 (0.3%)	0 (0%)	0 (0%)	1 (0.3%)	0 (0%)
Pharyngitis	1 (0.3%)	1 (0.3%)	0 (0%)	0 (0%)	1 (0.3%)	0 (0%)	0 (0%)	1 (0.3%)	0 (0%)
Pharyngitis streptococcal	1 (0.3%)	1 (0.3%)	0 (0%)	0 (0%)	1 (0.3%)	0 (0%)	0 (0%)	1 (0.3%)	0 (0%)
Pneumonia aspiration	1 (0.3%)	1 (0.3%)	0 (0%)	0 (0%)	0 (0%)	0 (0%)	1 (0.3%)	1 (0.3%)	0 (0%)
Respiratory syncytial virus infection	1 (0.3%)	1 (0.3%)	0 (0%)	0 (0%)	1 (0.3%)	0 (0%)	0 (0%)	1 (0.3%)	0 (0%)

© 2025 Bryan CJ et al. *JAMA Network Open*

		Related to Device? [a]			Severity [b]			Expected?	
Adverse Event Type & Subtype	Total	Definitely No	Possibly	Definitely Yes	Mild	Moderate	Severe	No	Yes
Rhinovirus infection	1 (0.3%)	1 (0.3%)	0 (0%)	0 (0%)	1 (0.3%)	0 (0%)	0 (0%)	1 (0.3%)	0 (0%)
Sepsis	1 (0.3%)	1 (0.3%)	0 (0%)	0 (0%)	0 (0%)	1 (0.3%)	0 (0%)	1 (0.3%)	0 (0%)
Sinusitis	3 (0.9%)	3 (0.9%)	0 (0%)	0 (0%)	2 (0.6%)	1 (0.3%)	0 (0%)	3 (0.9%)	0 (0%)
Upper respiratory tract infection	3 (0.9%)	3 (0.9%)	0 (0%)	0 (0%)	2 (0.6%)	1 (0.3%)	0 (0%)	3 (0.9%)	0 (0%)
Urinary tract infection	4 (1.2%)	4 (1.2%)	0 (0%)	0 (0%)	4 (1.2%)	0 (0%)	0 (0%)	4 (1.2%)	0 (0%)
Wound infection	1 (0.3%)	1 (0.3%)	0 (0%)	0 (0%)	0 (0%)	1 (0.3%)	0 (0%)	1 (0.3%)	0 (0%)
Injury, poisoning and procedural complications	23 (5.7%)	23 (5.7%)	0 (0%)	0 (0%)	9 (2.4%)	9 (2.4%)	4 (1.2%)	23 (5.7%)	0 (0%)
Accident at work	1 (0.3%)	1 (0.3%)	0 (0%)	0 (0%)	0 (0%)	1 (0.3%)	0 (0%)	1 (0.3%)	0 (0%)
Animal bite	1 (0.3%)	1 (0.3%)	0 (0%)	0 (0%)	1 (0.3%)	0 (0%)	0 (0%)	1 (0.3%)	0 (0%)
Arthropod bite	1 (0.3%)	1 (0.3%)	0 (0%)	0 (0%)	0 (0%)	1 (0.3%)	0 (0%)	1 (0.3%)	0 (0%)
Bursitis	1 (0.3%)	1 (0.3%)	0 (0%)	0 (0%)	1 (0.3%)	0 (0%)	0 (0%)	1 (0.3%)	0 (0%)
Concussion	1 (0.3%)	1 (0.3%)	0 (0%)	0 (0%)	1 (0.3%)	0 (0%)	0 (0%)	1 (0.3%)	0 (0%)
Fall	1 (0.3%)	1 (0.3%)	0 (0%)	0 (0%)	1 (0.3%)	0 (0%)	0 (0%)	1 (0.3%)	0 (0%)
Foreign body ingestion	1 (0.3%)	1 (0.3%)	0 (0%)	0 (0%)	0 (0%)	1 (0.3%)	0 (0%)	1 (0.3%)	0 (0%)
Gastrointestinal stoma complication	1 (0.3%)	1 (0.3%)	0 (0%)	0 (0%)	0 (0%)	0 (0%)	1 (0.3%)	1 (0.3%)	0 (0%)
Joint injury	1 (0.3%)	1 (0.3%)	0 (0%)	0 (0%)	1 (0.3%)	0 (0%)	0 (0%)	1 (0.3%)	0 (0%)
Ligament sprain	3 (0.9%)	3 (0.9%)	0 (0%)	0 (0%)	1 (0.3%)	1 (0.3%)	0 (0%)	3 (0.9%)	0 (0%)
Overdose	3 (0.9%)	3 (0.9%)	0 (0%)	0 (0%)	0 (0%)	0 (0%)	3 (0.9%)	3 (0.9%)	0 (0%)
Road traffic accident	2 (0.6%)	2 (0.6%)	0 (0%)	0 (0%)	1 (0.3%)	1 (0.3%)	0 (0%)	2 (0.6%)	0 (0%)
Skin laceration	3 (0.9%)	3 (0.9%)	0 (0%)	0 (0%)	1 (0.3%)	2 (0.6%)	0 (0%)	3 (0.9%)	0 (0%)
Sunburn	1 (0.3%)	1 (0.3%)	0 (0%)	0 (0%)	0 (0%)	1 (0.3%)	0 (0%)	1 (0.3%)	0 (0%)
Tendonitis	1 (0.3%)	1 (0.3%)	0 (0%)	0 (0%)	1 (0.3%)	0 (0%)	0 (0%)	1 (0.3%)	0 (0%)

© 2025 Bryan CJ et al. *JAMA Network Open*

		Related to Device? [a]			Severity [b]			Expected?	
**Adverse Event Type & Subtype**	**Total**	**Definitely No**	**Possibly**	**Definitely Yes**	**Mild**	**Moderate**	**Severe**	**No**	**Yes**
Thermal burn	1 (0.3%)	1 (0.3%)	0 (0%)	0 (0%)	0 (0%)	1 (0.3%)	0 (0%)	1 (0.3%)	0 (0%)
Investigations	4 (1.2%)	4 (1.2%)	0 (0%)	0 (0%)	2 (0.6%)	2 (0.6%)	0 (0%)	4 (1.2%)	0 (0%)
Blood urine present	1 (0.3%)	1 (0.3%)	0 (0%)	0 (0%)	1 (0.3%)	0 (0%)	0 (0%)	1 (0.3%)	0 (0%)
Psychiatric evaluation	1 (0.3%)	1 (0.3%)	0 (0%)	0 (0%)	0 (0%)	1 (0.3%)	0 (0%)	1 (0.3%)	0 (0%)
Sexually transmitted disease test	1 (0.3%)	1 (0.3%)	0 (0%)	0 (0%)	1 (0.3%)	0 (0%)	0 (0%)	1 (0.3%)	0 (0%)
Smear cervix abnormal	1 (0.3%)	1 (0.3%)	0 (0%)	0 (0%)	0 (0%)	1 (0.3%)	0 (0%)	1 (0.3%)	0 (0%)
Metabolism and nutrition disorders	4 (1.2%)	4 (1.2%)	0 (0%)	0 (0%)	3 (0.9%)	0 (0%)	1 (0.3%)	4 (1.2%)	0 (0%)
Dehydration	2 (0.6%)	2 (0.6%)	0 (0%)	0 (0%)	2 (0.6%)	0 (0%)	0 (0%)	2 (0.6%)	0 (0%)
Hypoglycaemia	1 (0.3%)	1 (0.3%)	0 (0%)	0 (0%)	0 (0%)	0 (0%)	1 (0.3%)	1 (0.3%)	0 (0%)
Vitamin D deficiency	1 (0.3%)	1 (0.3%)	0 (0%)	0 (0%)	1 (0.3%)	0 (0%)	0 (0%)	1 (0.3%)	0 (0%)
Musculoskeletal and connective tissue disorders	6 (1.8%)	6 (1.8%)	0 (0%)	0 (0%)	1 (0.3%)	5 (1.5%)	0 (0%)	6 (1.8%)	0 (0%)
Ankle fracture	1 (0.3%)	1 (0.3%)	0 (0%)	0 (0%)	0 (0%)	1 (0.3%)	0 (0%)	1 (0.3%)	0 (0%)
Arthralgia	3 (0.9%)	3 (0.9%)	0 (0%)	0 (0%)	0 (0%)	3 (0.9%)	0 (0%)	3 (0.9%)	0 (0%)
Costochondritis	1 (0.3%)	1 (0.3%)	0 (0%)	0 (0%)	0 (0%)	1 (0.3%)	0 (0%)	1 (0.3%)	0 (0%)
Pain in extremity	1 (0.3%)	1 (0.3%)	0 (0%)	0 (0%)	1 (0.3%)	0 (0%)	0 (0%)	1 (0.3%)	0 (0%)
Nervous system disorders	21 (6.0%)	20 (5.7%)	0 (0%)	0 (0%)	14 (4.2%)	5 (1.2%)	1 (0.3%)	21 (6.0%)	0 (0%)
Dizziness	1 (0.3%)	1 (0.3%)	0 (0%)	0 (0%)	0 (0%)	0 (0%)	1 (0.3%)	1 (0.3%)	0 (0%)
Headache	6 (1.8%)	5 (1.5%)	0 (0%)	0 (0%)	5 (1.5%)	0 (0%)	0 (0%)	6 (1.8%)	0 (0%)
Hypoaesthesia	3 (0.9%)	3 (0.9%)	0 (0%)	0 (0%)	3 (0.9%)	0 (0%)	0 (0%)	3 (0.9%)	0 (0%)
Loss of consciousness	1 (0.3%)	1 (0.3%)	0 (0%)	0 (0%)	1 (0.3%)	0 (0%)	0 (0%)	1 (0.3%)	0 (0%)
Migraine	4 (1.2%)	4 (1.2%)	0 (0%)	0 (0%)	2 (0.6%)	2 (0.6%)	0 (0%)	4 (1.2%)	0 (0%)
Myoclonic epilepsy	1 (0.3%)	1 (0.3%)	0 (0%)	0 (0%)	1 (0.3%)	0 (0%)	0 (0%)	1 (0.3%)	0 (0%)
Paraesthesia	1 (0.3%)	1 (0.3%)	0 (0%)	0 (0%)	1 (0.3%)	0 (0%)	0 (0%)	1 (0.3%)	0 (0%)

© 2025 Bryan CJ et al. *JAMA Network Open*

		Related to Device? [a]			Severity [b]			Expected?	
Adverse Event Type & Subtype	Total	Definitely No	Possibly	Definitely Yes	Mild	Moderate	Severe	No	Yes
Sciatica	1 (0.3%)	1 (0.3%)	0 (0%)	0 (0%)	0 (0%)	1 (0.3%)	0 (0%)	1 (0.3%)	0 (0%)
Seizure	2 (0.6%)	2 (0.6%)	0 (0%)	0 (0%)	1 (0.3%)	1 (0.3%)	0 (0%)	2 (0.6%)	0 (0%)
Tremor	1 (0.3%)	1 (0.3%)	0 (0%)	0 (0%)	0 (0%)	1 (0.3%)	0 (0%)	1 (0.3%)	0 (0%)
Pregnancy, puerperium and perinatal conditions	1 (0.3%)	1 (0.3%)	0 (0%)	0 (0%)	0 (0%)	1 (0.3%)	0 (0%)	1 (0.3%)	0 (0%)
Abortion spontaneous	1 (0.3%)	1 (0.3%)	0 (0%)	0 (0%)	0 (0%)	1 (0.3%)	0 (0%)	1 (0.3%)	0 (0%)
Product issues	2 (0.6%)	1 (0.3%)	0 (0%)	1 (0.3%)	2 (0.6%)	0 (0%)	0 (0%)	2 (0.6%)	0 (0%)
Device malfunction	1 (0.3%)	0 (0%)	0 (0%)	1 (0.3%)	1 (0.3%)	0 (0%)	0 (0%)	1 (0.3%)	0 (0%)
Embedded device	1 (0.3%)	1 (0.3%)	0 (0%)	0 (0%)	1 (0.3%)	0 (0%)	0 (0%)	1 (0.3%)	0 (0%)
Psychiatric disorders	118 (26.9%)	111 (26.0%)	6 (1.5%)	1 (0.3%)	30 (7.9%)	39 (11.2%)	48 (12.4%)	107 (25.4%)	11 (2.7%)
Anxiety	6 (1.8%)	5 (1.5%)	1 (0.3%)	0 (0%)	4 (1.2%)	2 (0.6%)	0 (0%)	5 (1.5%)	1 (0.3%)
Bipolar I disorder	1 (0.3%)	1 (0.3%)	0 (0%)	0 (0%)	1 (0.3%)	0 (0%)	0 (0%)	1 (0.3%)	0 (0%)
Bipolar II disorder	1 (0.3%)	1 (0.3%)	0 (0%)	0 (0%)	1 (0.3%)	0 (0%)	0 (0%)	1 (0.3%)	0 (0%)
Bipolar disorder	1 (0.3%)	1 (0.3%)	0 (0%)	0 (0%)	1 (0.3%)	0 (0%)	0 (0%)	1 (0.3%)	0 (0%)
Borderline personality disorder	3 (0.9%)	3 (0.9%)	0 (0%)	0 (0%)	3 (0.9%)	0 (0%)	0 (0%)	3 (0.9%)	0 (0%)
Completed suicide	1 (0.3%)	1 (0.3%)	0 (0%)	0 (0%)	0 (0%)	0 (0%)	1 (0.3%)	1 (0.3%)	0 (0%)
Depression	4 (1.2%)	4 (1.2%)	0 (0%)	0 (0%)	0 (0%)	3 (0.9%)	1 (0.3%)	3 (0.9%)	1 (0.3%)
Eating disorder	4 (1.2%)	4 (1.2%)	0 (0%)	0 (0%)	1 (0.3%)	1 (0.3%)	2 (0.6%)	4 (1.2%)	0 (0%)
Eating disorder symptom	1 (0.3%)	1 (0.3%)	0 (0%)	0 (0%)	0 (0%)	1 (0.3%)	0 (0%)	1 (0.3%)	0 (0%)
Hypomania	1 (0.3%)	1 (0.3%)	0 (0%)	0 (0%)	1 (0.3%)	0 (0%)	0 (0%)	1 (0.3%)	0 (0%)
Insomnia	1 (0.3%)	1 (0.3%)	0 (0%)	0 (0%)	1 (0.3%)	0 (0%)	0 (0%)	1 (0.3%)	0 (0%)
Intentional self-injury	5 (1.5%)	4 (1.2%)	1 (0.3%)	0 (0%)	2 (0.6%)	2 (0.6%)	1 (0.3%)	5 (1.5%)	0 (0%)
Major depression	2 (0.6%)	2 (0.6%)	0 (0%)	0 (0%)	2 (0.6%)	0 (0%)	0 (0%)	2 (0.6%)	0 (0%)
Panic attack	2 (0.6%)	2 (0.6%)	0 (0%)	0 (0%)	0 (0%)	2 (0.6%)	0 (0%)	2 (0.6%)	0 (0%)

© 2025 Bryan CJ et al. *JAMA Network Open*

		Related to Device? [a]			Severity [b]			Expected?	
**Adverse Event Type & Subtype**	**Total**	**Definitely No**	**Possibly**	**Definitely Yes**	**Mild**	**Moderate**	**Severe**	**No**	**Yes**
Post-traumatic stress disorder	1 (0.3%)	1 (0.3%)	0 (0%)	0 (0%)	1 (0.3%)	0 (0%)	0 (0%)	1 (0.3%)	0 (0%)
Psychotic disorder	1 (0.3%)	1 (0.3%)	0 (0%)	0 (0%)	0 (0%)	1 (0.3%)	0 (0%)	1 (0.3%)	0 (0%)
Restlessness	1 (0.3%)	1 (0.3%)	0 (0%)	0 (0%)	1 (0.3%)	0 (0%)	0 (0%)	1 (0.3%)	0 (0%)
Suicidal ideation	43 (13.0%)	40 (12.1%)	2 (0.6%)	1 (0.3%)	9 (2.7%)	13 (3.9%)	20 (6.0%)	38 (11.5%)	5 (1.5%)
Suicide attempt	39 (11.8%)	37 (11.2%)	2 (0.6%)	0 (0%)	2 (0.6%)	14 (4.2%)	23 (6.9%)	35 (10.6%)	4 (1.2%)
Renal and urinary disorders	2 (0.6%)	2 (0.6%)	0 (0%)	0 (0%)	2 (0.6%)	0 (0%)	0 (0%)	2 (0.6%)	0 (0%)
Nephrolithiasis	1 (0.3%)	1 (0.3%)	0 (0%)	0 (0%)	1 (0.3%)	0 (0%)	0 (0%)	1 (0.3%)	0 (0%)
Pollakiuria	1 (0.3%)	1 (0.3%)	0 (0%)	0 (0%)	1 (0.3%)	0 (0%)	0 (0%)	1 (0.3%)	0 (0%)
Reproductive system and breast disorders	3 (0.6%)	3 (0.6%)	0 (0%)	0 (0%)	1 (0.3%)	0 (0%)	2 (0.3%)	3 (0.6%)	0 (0%)
Ovarian cyst	1 (0.3%)	1 (0.3%)	0 (0%)	0 (0%)	1 (0.3%)	0 (0%)	0 (0%)	1 (0.3%)	0 (0%)
Ovulation pain	1 (0.3%)	1 (0.3%)	0 (0%)	0 (0%)	0 (0%)	0 (0%)	1 (0.3%)	1 (0.3%)	0 (0%)
Pelvic pain	1 (0.3%)	1 (0.3%)	0 (0%)	0 (0%)	0 (0%)	0 (0%)	1 (0.3%)	1 (0.3%)	0 (0%)
Respiratory, thoracic and mediastinal disorders	4 (1.2%)	4 (1.2%)	0 (0%)	0 (0%)	1 (0.3%)	3 (0.9%)	0 (0%)	4 (1.2%)	0 (0%)
Chronic obstructive pulmonary disease	1 (0.3%)	1 (0.3%)	0 (0%)	0 (0%)	0 (0%)	1 (0.3%)	0 (0%)	1 (0.3%)	0 (0%)
Hypoxia	1 (0.3%)	1 (0.3%)	0 (0%)	0 (0%)	0 (0%)	1 (0.3%)	0 (0%)	1 (0.3%)	0 (0%)
Nasal congestion	1 (0.3%)	1 (0.3%)	0 (0%)	0 (0%)	1 (0.3%)	0 (0%)	0 (0%)	1 (0.3%)	0 (0%)
Tonsillar hypertrophy	1 (0.3%)	1 (0.3%)	0 (0%)	0 (0%)	0 (0%)	1 (0.3%)	0 (0%)	1 (0.3%)	0 (0%)
Skin and subcutaneous tissue disorders	5 (1.5%)	5 (1.5%)	0 (0%)	0 (0%)	3 (0.9%)	1 (0.3%)	1 (0.3%)	5 (1.5%)	0 (0%)
Dermatitis	1 (0.3%)	1 (0.3%)	0 (0%)	0 (0%)	0 (0%)	0 (0%)	1 (0.3%)	1 (0.3%)	0 (0%)
Granuloma skin	1 (0.3%)	1 (0.3%)	0 (0%)	0 (0%)	0 (0%)	1 (0.3%)	0 (0%)	1 (0.3%)	0 (0%)
Rash	1 (0.3%)	1 (0.3%)	0 (0%)	0 (0%)	1 (0.3%)	0 (0%)	0 (0%)	1 (0.3%)	0 (0%)
Scratch	1 (0.3%)	1 (0.3%)	0 (0%)	0 (0%)	1 (0.3%)	0 (0%)	0 (0%)	1 (0.3%)	0 (0%)

© 2025 Bryan CJ et al. *JAMA Network Open*

		Related to Device? [a]			Severity [b]			Expected?	
Adverse Event Type & Subtype	Total	Definitely No	Possibly	Definitely Yes	Mild	Moderate	Severe	No	Yes
Urticaria	1 (0.3%)	1 (0.3%)	0 (0%)	0 (0%)	1 (0.3%)	0 (0%)	0 (0%)	1 (0.3%)	0 (0%)
Surgical and medical procedures	4 (1.2%)	4 (1.2%)	0 (0%)	0 (0%)	0 (0%)	2 (0.6%)	2 (0.6%)	3 (0.9%)	1 (0.3%)
Colostomy	1 (0.3%)	1 (0.3%)	0 (0%)	0 (0%)	0 (0%)	1 (0.3%)	0 (0%)	1 (0.3%)	0 (0%)
Hospitalisation	3 (0.9%)	3 (0.9%)	0 (0%)	0 (0%)	0 (0%)	1 (0.3%)	2 (0.6%)	2 (0.6%)	1 (0.3%)
Uncoded [c]	110 (29.6%)	106 (28.7%)	2 (0.6%)	0 (0%)	82 (23.0%)	14 (3.6%)	11 (3.3%)	110 (29.6%)	0 (0%)
Acute on chronic pancreatitis	1 (0.3%)	1 (0.3%)	0 (0%)	0 (0%)	0 (0%)	1 (0.3%)	0 (0%)	1 (0.3%)	0 (0%)
COVID	1 (0.3%)	1 (0.3%)	0 (0%)	0 (0%)	1 (0.3%)	0 (0%)	0 (0%)	1 (0.3%)	0 (0%)
Crohn's disease exacerbation	1 (0.3%)	1 (0.3%)	0 (0%)	0 (0%)	0 (0%)	1 (0.3%)	0 (0%)	1 (0.3%)	0 (0%)
ED Visit for Viral Gastroenteritis	1 (0.3%)	1 (0.3%)	0 (0%)	0 (0%)	0 (0%)	1 (0.3%)	0 (0%)	1 (0.3%)	0 (0%)
ED appearance and subsequent psychiat	1 (0.3%)	1 (0.3%)	0 (0%)	0 (0%)	0 (0%)	0 (0%)	1 (0.3%)	1 (0.3%)	0 (0%)
ED appearance for alcohol intoxicatio	1 (0.3%)	1 (0.3%)	0 (0%)	0 (0%)	0 (0%)	1 (0.3%)	0 (0%)	1 (0.3%)	0 (0%)
ED appearance for dizziness	1 (0.3%)	1 (0.3%)	0 (0%)	0 (0%)	1 (0.3%)	0 (0%)	0 (0%)	1 (0.3%)	0 (0%)
ED appearance for lower GI bleed	1 (0.3%)	1 (0.3%)	0 (0%)	0 (0%)	1 (0.3%)	0 (0%)	0 (0%)	1 (0.3%)	0 (0%)
ED presentation	5 (1.5%)	5 (1.5%)	0 (0%)	0 (0%)	0 (0%)	2 (0.6%)	3 (0.9%)	5 (1.5%)	0 (0%)
ED presentation for SI	1 (0.3%)	1 (0.3%)	0 (0%)	0 (0%)	0 (0%)	0 (0%)	1 (0.3%)	1 (0.3%)	0 (0%)
ED visit and subsequent psychiatric a	1 (0.3%)	1 (0.3%)	0 (0%)	0 (0%)	0 (0%)	0 (0%)	1 (0.3%)	1 (0.3%)	0 (0%)
Hospitalization due to a preexisting	1 (0.3%)	1 (0.3%)	0 (0%)	0 (0%)	0 (0%)	1 (0.3%)	0 (0%)	1 (0.3%)	0 (0%)
Hospitalization for management of chr	1 (0.3%)	1 (0.3%)	0 (0%)	0 (0%)	1 (0.3%)	0 (0%)	0 (0%)	1 (0.3%)	0 (0%)
Increased Suicidal Ideation from Base	1 (0.3%)	1 (0.3%)	0 (0%)	0 (0%)	0 (0%)	1 (0.3%)	0 (0%)	1 (0.3%)	0 (0%)
Low Vitamin D Level	1 (0.3%)	1 (0.3%)	0 (0%)	0 (0%)	1 (0.3%)	0 (0%)	0 (0%)	1 (0.3%)	0 (0%)

© 2025 Bryan CJ et al. *JAMA Network Open*

		Related to Device? [a]			Severity [b]			Expected?	
Adverse Event Type & Subtype	Total	Definitely No	Possibly	Definitely Yes	Mild	Moderate	Severe	No	Yes
Missed Visit	75 (22.7%)	73 (22.1%)	1 (0.3%)	0 (0%)	69 (20.8%)	1 (0.3%)	4 (1.2%)	75 (22.7%)	0 (0%)
Non suicidal self harm (cutting) need	1 (0.3%)	0 (0%)	1 (0.3%)	0 (0%)	0 (0%)	1 (0.3%)	0 (0%)	1 (0.3%)	0 (0%)
Other	1 (0.3%)	0 (0%)	0 (0%)	0 (0%)	0 (0%)	0 (0%)	0 (0%)	1 (0.3%)	0 (0%)
Risk Concern - Per Outpatient Provide	1 (0.3%)	1 (0.3%)	0 (0%)	0 (0%)	1 (0.3%)	0 (0%)	0 (0%)	1 (0.3%)	0 (0%)
Suicidal Ideation with preparatory ac	1 (0.3%)	1 (0.3%)	0 (0%)	0 (0%)	1 (0.3%)	0 (0%)	0 (0%)	1 (0.3%)	0 (0%)
Suicide attempt 08Jan2024	1 (0.3%)	1 (0.3%)	0 (0%)	0 (0%)	0 (0%)	0 (0%)	0 (0%)	1 (0.3%)	0 (0%)
Suicide attempt via self-strangulatio	1 (0.3%)	1 (0.3%)	0 (0%)	0 (0%)	1 (0.3%)	0 (0%)	0 (0%)	1 (0.3%)	0 (0%)
Tracheal Dilation- outpatient procedu	1 (0.3%)	1 (0.3%)	0 (0%)	0 (0%)	0 (0%)	1 (0.3%)	0 (0%)	1 (0.3%)	0 (0%)
Tracheal dilation- outpatient procedu	1 (0.3%)	1 (0.3%)	0 (0%)	0 (0%)	0 (0%)	1 (0.3%)	0 (0%)	1 (0.3%)	0 (0%)
Urgent care appearance for Dizziness	1 (0.3%)	1 (0.3%)	0 (0%)	0 (0%)	1 (0.3%)	0 (0%)	0 (0%)	1 (0.3%)	0 (0%)
Urgent care appearance for abdominal	1 (0.3%)	1 (0.3%)	0 (0%)	0 (0%)	0 (0%)	1 (0.3%)	0 (0%)	1 (0.3%)	0 (0%)
Urgent care visit for back/arm pain	1 (0.3%)	1 (0.3%)	0 (0%)	0 (0%)	1 (0.3%)	0 (0%)	0 (0%)	1 (0.3%)	0 (0%)
Urgent care visit for upper respirato	1 (0.3%)	1 (0.3%)	0 (0%)	0 (0%)	1 (0.3%)	0 (0%)	0 (0%)	1 (0.3%)	0 (0%)
Withdrawal due to study involvement	1 (0.3%)	1 (0.3%)	0 (0%)	0 (0%)	1 (0.3%)	0 (0%)	0 (0%)	1 (0.3%)	0 (0%)
left ankle pain	1 (0.3%)	1 (0.3%)	0 (0%)	0 (0%)	1 (0.3%)	0 (0%)	0 (0%)	1 (0.3%)	0 (0%)
suicide attempt via ataraxic ingestio	1 (0.3%)	1 (0.3%)	0 (0%)	0 (0%)	0 (0%)	0 (0%)	1 (0.3%)	1 (0.3%)	0 (0%)
worsening anxiety	1 (0.3%)	1 (0.3%)	0 (0%)	0 (0%)	0 (0%)	1 (0.3%)	0 (0%)	1 (0.3%)	0 (0%)

© 2025 Bryan CJ et al. *JAMA Network Open*

		Related to Device? [a]			Severity [b]			Expected?	
**Adverse Event Type & Subtype**	**Total**	**Definitely No**	**Possibly**	**Definitely Yes**	**Mild**	**Moderate**	**Severe**	**No**	**Yes**
Vascular disorders	6 (1.8%)	6 (1.8%)	0 (0%)	0 (0%)	4 (1.2%)	2 (0.6%)	0 (0%)	6 (1.8%)	0 (0%)
Haematochezia	1 (0.3%)	1 (0.3%)	0 (0%)	0 (0%)	0 (0%)	1 (0.3%)	0 (0%)	1 (0.3%)	0 (0%)
Hypertension	1 (0.3%)	1 (0.3%)	0 (0%)	0 (0%)	1 (0.3%)	0 (0%)	0 (0%)	1 (0.3%)	0 (0%)
Hypotension	1 (0.3%)	1 (0.3%)	0 (0%)	0 (0%)	1 (0.3%)	0 (0%)	0 (0%)	1 (0.3%)	0 (0%)
Peripheral coldness	1 (0.3%)	1 (0.3%)	0 (0%)	0 (0%)	1 (0.3%)	0 (0%)	0 (0%)	1 (0.3%)	0 (0%)
Rectal haemorrhage	1 (0.3%)	1 (0.3%)	0 (0%)	0 (0%)	0 (0%)	1 (0.3%)	0 (0%)	1 (0.3%)	0 (0%)
Vaginal haemorrhage	1 (0.3%)	1 (0.3%)	0 (0%)	0 (0%)	1 (0.3%)	0 (0%)	0 (0%)	1 (0.3%)	0 (0%)

Notes:
- Table entries represent: # of AEs (% of Subjects experiencing the event)
- Participants experiencing an event within a given PT and SOC more than once will be counted under the maximum severity/relationship experienced.
[a] Based on n=394 due to missing data from 4 participants
[b] Based on n=391 due to missing data from 7 participants
[c] Uncoded AEs are as reported by site.

© 2025 Bryan CJ et al. *JAMA Network Open*

eTable 4. Adverse events (AEs) reported by participants assigned to OTX-202 (n=164)

		Related to Device? [a]			Severity [b]			Expected?	
	**Total**	**Definitely No**	**Possibly**	**Definitely Yes**	**Mild**	**Moderate**	**Severe**	**No**	**Yes**
**Any AEs**	198 (64.0%)	192 (62.8%)	3 (1.8%)	2 (1.2%)	106 (41.5%)	50 (24.4%)	40 (20.1%)	189 (64.0%)	9 (4.3%)
Blood and lymphatic system disorders	2 (1.2%)	2 (1.2%)	0 (0%)	0 (0%)	2 (1.2%)	0 (0%)	0 (0%)	2 (1.2%)	0 (0%)
Lymphadenopathy	1 (0.6%)	1 (0.6%)	0 (0%)	0 (0%)	1 (0.6%)	0 (0%)	0 (0%)	1 (0.6%)	0 (0%)
Thrombocytosis	1 (0.6%)	1 (0.6%)	0 (0%)	0 (0%)	1 (0.6%)	0 (0%)	0 (0%)	1 (0.6%)	0 (0%)
Cardiac disorders	2 (1.2%)	2 (1.2%)	0 (0%)	0 (0%)	2 (1.2%)	0 (0%)	0 (0%)	2 (1.2%)	0 (0%)
Chest pain	2 (1.2%)	2 (1.2%)	0 (0%)	0 (0%)	2 (1.2%)	0 (0%)	0 (0%)	2 (1.2%)	0 (0%)
Gastrointestinal disorders	7 (3.7%)	6 (3.0%)	0 (0%)	0 (0%)	5 (3.0%)	1 (0.6%)	0 (0%)	7 (3.7%)	0 (0%)
Abdominal pain	3 (1.8%)	2 (1.2%)	0 (0%)	0 (0%)	1 (0.6%)	1 (0.6%)	0 (0%)	3 (1.8%)	0 (0%)
Nausea	1 (0.6%)	1 (0.6%)	0 (0%)	0 (0%)	1 (0.6%)	0 (0%)	0 (0%)	1 (0.6%)	0 (0%)
Pancreatitis chronic	1 (0.6%)	1 (0.6%)	0 (0%)	0 (0%)	1 (0.6%)	0 (0%)	0 (0%)	1 (0.6%)	0 (0%)
Salivary hypersecretion	1 (0.6%)	1 (0.6%)	0 (0%)	0 (0%)	1 (0.6%)	0 (0%)	0 (0%)	1 (0.6%)	0 (0%)
Vomiting	1 (0.6%)	1 (0.6%)	0 (0%)	0 (0%)	1 (0.6%)	0 (0%)	0 (0%)	1 (0.6%)	0 (0%)
General disorders and administration site conditions	8 (3.7%)	8 (3.7%)	0 (0%)	0 (0%)	6 (2.4%)	1 (0.6%)	1 (0.6%)	8 (3.7%)	0 (0%)
Adverse drug reaction	4 (2.4%)	4 (2.4%)	0 (0%)	0 (0%)	3 (1.8%)	1 (0.6%)	0 (0%)	4 (2.4%)	0 (0%)
Death	1 (0.6%)	1 (0.6%)	0 (0%)	0 (0%)	0 (0%)	0 (0%)	1 (0.6%)	1 (0.6%)	0 (0%)
Drug withdrawal syndrome	1 (0.6%)	1 (0.6%)	0 (0%)	0 (0%)	1 (0.6%)	0 (0%)	0 (0%)	1 (0.6%)	0 (0%)
Fatigue	1 (0.6%)	1 (0.6%)	0 (0%)	0 (0%)	1 (0.6%)	0 (0%)	0 (0%)	1 (0.6%)	0 (0%)
Oedema	1 (0.6%)	1 (0.6%)	0 (0%)	0 (0%)	1 (0.6%)	0 (0%)	0 (0%)	1 (0.6%)	0 (0%)
Immune system disorders	3 (1.8%)	3 (1.8%)	0 (0%)	0 (0%)	1 (0.6%)	2 (1.2%)	0 (0%)	3 (1.8%)	0 (0%)

© 2025 Bryan CJ et al. *JAMA Network Open*

		Related to Device? [a]			Severity [b]			Expected?	
	Total	Definitely No	Possibly	Definitely Yes	Mild	Moderate	Severe	No	Yes
Asthma	1 (0.6%)	1 (0.6%)	0 (0%)	0 (0%)	0 (0%)	1 (0.6%)	0 (0%)	1 (0.6%)	0 (0%)
Hypersensitivity	2 (1.2%)	2 (1.2%)	0 (0%)	0 (0%)	1 (0.6%)	1 (0.6%)	0 (0%)	2 (1.2%)	0 (0%)
Infections and infestations	21 (10.4%)	21 (10.4%)	0 (0%)	0 (0%)	15 (7.9%)	5 (3.0%)	1 (0.6%)	21 (10.4%)	0 (0%)
Abscess	1 (0.6%)	1 (0.6%)	0 (0%)	0 (0%)	0 (0%)	1 (0.6%)	0 (0%)	1 (0.6%)	0 (0%)
Bronchitis	1 (0.6%)	1 (0.6%)	0 (0%)	0 (0%)	1 (0.6%)	0 (0%)	0 (0%)	1 (0.6%)	0 (0%)
COVID-19	6 (3.7%)	6 (3.7%)	0 (0%)	0 (0%)	3 (1.8%)	3 (1.8%)	0 (0%)	6 (3.7%)	0 (0%)
Herpes simplex	1 (0.6%)	1 (0.6%)	0 (0%)	0 (0%)	1 (0.6%)	0 (0%)	0 (0%)	1 (0.6%)	0 (0%)
Influenza	1 (0.6%)	1 (0.6%)	0 (0%)	0 (0%)	1 (0.6%)	0 (0%)	0 (0%)	1 (0.6%)	0 (0%)
Nasopharyngitis	4 (2.4%)	4 (2.4%)	0 (0%)	0 (0%)	4 (2.4%)	0 (0%)	0 (0%)	4 (2.4%)	0 (0%)
Pharyngitis streptococcal	1 (0.6%)	1 (0.6%)	0 (0%)	0 (0%)	1 (0.6%)	0 (0%)	0 (0%)	1 (0.6%)	0 (0%)
Pneumonia aspiration	1 (0.6%)	1 (0.6%)	0 (0%)	0 (0%)	0 (0%)	0 (0%)	1 (0.6%)	1 (0.6%)	0 (0%)
Sinusitis	1 (0.6%)	1 (0.6%)	0 (0%)	0 (0%)	1 (0.6%)	0 (0%)	0 (0%)	1 (0.6%)	0 (0%)
Upper respiratory tract infection	2 (1.2%)	2 (1.2%)	0 (0%)	0 (0%)	1 (0.6%)	1 (0.6%)	0 (0%)	2 (1.2%)	0 (0%)
Urinary tract infection	2 (1.2%)	2 (1.2%)	0 (0%)	0 (0%)	2 (1.2%)	0 (0%)	0 (0%)	2 (1.2%)	0 (0%)
Injury, poisoning and procedural complications	9 (4.9%)	9 (4.9%)	0 (0%)	0 (0%)	2 (1.2%)	4 (2.4%)	3 (1.8%)	9 (4.9%)	0 (0%)
Accident at work	1 (0.6%)	1 (0.6%)	0 (0%)	0 (0%)	0 (0%)	1 (0.6%)	0 (0%)	1 (0.6%)	0 (0%)
Concussion	1 (0.6%)	1 (0.6%)	0 (0%)	0 (0%)	1 (0.6%)	0 (0%)	0 (0%)	1 (0.6%)	0 (0%)
Foreign body ingestion	1 (0.6%)	1 (0.6%)	0 (0%)	0 (0%)	0 (0%)	1 (0.6%)	0 (0%)	1 (0.6%)	0 (0%)
Ligament sprain	1 (0.6%)	1 (0.6%)	0 (0%)	0 (0%)	0 (0%)	1 (0.6%)	0 (0%)	1 (0.6%)	0 (0%)
Overdose	3 (1.8%)	3 (1.8%)	0 (0%)	0 (0%)	0 (0%)	0 (0%)	3 (1.8%)	3 (1.8%)	0 (0%)
Road traffic accident	1 (0.6%)	1 (0.6%)	0 (0%)	0 (0%)	1 (0.6%)	0 (0%)	0 (0%)	1 (0.6%)	0 (0%)

© 2025 Bryan CJ et al. *JAMA Network Open*

		Related to Device? [a]			Severity [b]			Expected?	
	Total	Definitely No	Possibly	Definitely Yes	Mild	Moderate	Severe	No	Yes
Sunburn	1 (0.6%)	1 (0.6%)	0 (0%)	0 (0%)	0 (0%)	1 (0.6%)	0 (0%)	1 (0.6%)	0 (0%)
Investigations	3 (1.8%)	3 (1.8%)	0 (0%)	0 (0%)	1 (0.6%)	2 (1.2%)	0 (0%)	3 (1.8%)	0 (0%)
Psychiatric evaluation	1 (0.6%)	1 (0.6%)	0 (0%)	0 (0%)	0 (0%)	1 (0.6%)	0 (0%)	1 (0.6%)	0 (0%)
Sexually transmitted disease test	1 (0.6%)	1 (0.6%)	0 (0%)	0 (0%)	1 (0.6%)	0 (0%)	0 (0%)	1 (0.6%)	0 (0%)
Smear cervix abnormal	1 (0.6%)	1 (0.6%)	0 (0%)	0 (0%)	0 (0%)	1 (0.6%)	0 (0%)	1 (0.6%)	0 (0%)
Metabolism and nutrition disorders	2 (1.2%)	2 (1.2%)	0 (0%)	0 (0%)	2 (1.2%)	0 (0%)	0 (0%)	2 (1.2%)	0 (0%)
Dehydration	2 (1.2%)	2 (1.2%)	0 (0%)	0 (0%)	2 (1.2%)	0 (0%)	0 (0%)	2 (1.2%)	0 (0%)
Musculoskeletal and connective tissue disorders	3 (1.8%)	3 (1.8%)	0 (0%)	0 (0%)	1 (0.6%)	2 (1.2%)	0 (0%)	3 (1.8%)	0 (0%)
Arthralgia	1 (0.6%)	1 (0.6%)	0 (0%)	0 (0%)	0 (0%)	1 (0.6%)	0 (0%)	1 (0.6%)	0 (0%)
Costochondritis	1 (0.6%)	1 (0.6%)	0 (0%)	0 (0%)	0 (0%)	1 (0.6%)	0 (0%)	1 (0.6%)	0 (0%)
Pain in extremity	1 (0.6%)	1 (0.6%)	0 (0%)	0 (0%)	1 (0.6%)	0 (0%)	0 (0%)	1 (0.6%)	0 (0%)
Nervous system disorders	8 (4.9%)	8 (4.9%)	0 (0%)	0 (0%)	7 (4.3%)	1 (0.6%)	0 (0%)	8 (4.9%)	0 (0%)
Headache	2 (1.2%)	2 (1.2%)	0 (0%)	0 (0%)	2 (1.2%)	0 (0%)	0 (0%)	2 (1.2%)	0 (0%)
Hypoaesthesia	2 (1.2%)	2 (1.2%)	0 (0%)	0 (0%)	2 (1.2%)	0 (0%)	0 (0%)	2 (1.2%)	0 (0%)
Loss of consciousness	1 (0.6%)	1 (0.6%)	0 (0%)	0 (0%)	1 (0.6%)	0 (0%)	0 (0%)	1 (0.6%)	0 (0%)
Migraine	1 (0.6%)	1 (0.6%)	0 (0%)	0 (0%)	1 (0.6%)	0 (0%)	0 (0%)	1 (0.6%)	0 (0%)
Paraesthesia	1 (0.6%)	1 (0.6%)	0 (0%)	0 (0%)	1 (0.6%)	0 (0%)	0 (0%)	1 (0.6%)	0 (0%)
Sciatica	1 (0.6%)	1 (0.6%)	0 (0%)	0 (0%)	0 (0%)	1 (0.6%)	0 (0%)	1 (0.6%)	0 (0%)
Product issues	1 (0.6%)	0 (0%)	0 (0%)	1 (0.6%)	1 (0.6%)	0 (0%)	0 (0%)	1 (0.6%)	0 (0%)
Device malfunction	1 (0.6%)	0 (0%)	0 (0%)	1 (0.6%)	1 (0.6%)	0 (0%)	0 (0%)	1 (0.6%)	0 (0%)
Psychiatric disorders	67 (30.5%)	64 (29.3%)	2 (1.2%)	1 (0.6%)	15 (7.9%)	23 (13.4%)	29 (15.2%)	58 (28.0%)	9 (4.3%)

© 2025 Bryan CJ et al. *JAMA Network Open*

		Related to Device? [a]			Severity [b]			Expected?	
	Total	Definitely No	Possibly	Definitely Yes	Mild	Moderate	Severe	No	Yes
Anxiety	1 (0.6%)	1 (0.6%)	0 (0%)	0 (0%)	0 (0%)	1 (0.6%)	0 (0%)	1 (0.6%)	0 (0%)
Bipolar disorder	1 (0.6%)	1 (0.6%)	0 (0%)	0 (0%)	1 (0.6%)	0 (0%)	0 (0%)	1 (0.6%)	0 (0%)
Borderline personality disorder	2 (1.2%)	2 (1.2%)	0 (0%)	0 (0%)	2 (1.2%)	0 (0%)	0 (0%)	2 (1.2%)	0 (0%)
Depression	4 (2.4%)	4 (2.4%)	0 (0%)	0 (0%)	0 (0%)	3 (1.8%)	1 (0.6%)	3 (1.8%)	1 (0.6%)
Eating disorder	4 (2.4%)	4 (2.4%)	0 (0%)	0 (0%)	1 (0.6%)	1 (0.6%)	2 (1.2%)	4 (2.4%)	0 (0%)
Eating disorder symptom	1 (0.6%)	1 (0.6%)	0 (0%)	0 (0%)	0 (0%)	1 (0.6%)	0 (0%)	1 (0.6%)	0 (0%)
Insomnia	1 (0.6%)	1 (0.6%)	0 (0%)	0 (0%)	1 (0.6%)	0 (0%)	0 (0%)	1 (0.6%)	0 (0%)
Intentional self-injury	2 (1.2%)	2 (1.2%)	0 (0%)	0 (0%)	1 (0.6%)	0 (0%)	1 (0.6%)	2 (1.2%)	0 (0%)
Major depression	1 (0.6%)	1 (0.6%)	0 (0%)	0 (0%)	1 (0.6%)	0 (0%)	0 (0%)	1 (0.6%)	0 (0%)
Post-traumatic stress disorder	1 (0.6%)	1 (0.6%)	0 (0%)	0 (0%)	1 (0.6%)	0 (0%)	0 (0%)	1 (0.6%)	0 (0%)
Suicidal ideation	27 (16.5%)	25 (15.2%)	1 (0.6%)	1 (0.6%)	6 (3.7%)	7 (4.3%)	14 (8.5%)	23 (14.0%)	4 (2.4%)
Suicide attempt	22 (13.4%)	21 (12.8%)	1 (0.6%)	0 (0%)	1 (0.6%)	10 (6.1%)	11 (6.7%)	18 (11.0%)	4 (2.4%)
Renal and urinary disorders	2 (1.2%)	2 (1.2%)	0 (0%)	0 (0%)	2 (1.2%)	0 (0%)	0 (0%)	2 (1.2%)	0 (0%)
Nephrolithiasis	1 (0.6%)	1 (0.6%)	0 (0%)	0 (0%)	1 (0.6%)	0 (0%)	0 (0%)	1 (0.6%)	0 (0%)
Pollakiuria	1 (0.6%)	1 (0.6%)	0 (0%)	0 (0%)	1 (0.6%)	0 (0%)	0 (0%)	1 (0.6%)	0 (0%)
Respiratory, thoracic and mediastinal disorders	3 (1.8%)	3 (1.8%)	0 (0%)	0 (0%)	0 (0%)	3 (1.8%)	0 (0%)	3 (1.8%)	0 (0%)
Chronic obstructive pulmonary disease	1 (0.6%)	1 (0.6%)	0 (0%)	0 (0%)	0 (0%)	1 (0.6%)	0 (0%)	1 (0.6%)	0 (0%)
Hypoxia	1 (0.6%)	1 (0.6%)	0 (0%)	0 (0%)	0 (0%)	1 (0.6%)	0 (0%)	1 (0.6%)	0 (0%)
Tonsillar hypertrophy	1 (0.6%)	1 (0.6%)	0 (0%)	0 (0%)	0 (0%)	1 (0.6%)	0 (0%)	1 (0.6%)	0 (0%)
Surgical and medical procedures	1 (0.6%)	1 (0.6%)	0 (0%)	0 (0%)	0 (0%)	0 (0%)	1 (0.6%)	1 (0.6%)	0 (0%)
Hospitalisation	1 (0.6%)	1 (0.6%)	0 (0%)	0 (0%)	0 (0%)	0 (0%)	1 (0.6%)	1 (0.6%)	0 (0%)

© 2025 Bryan CJ et al. *JAMA Network Open*

		Related to Device? [a]			Severity [b]			Expected?	
	Total	Definitely No	Possibly	Definitely Yes	Mild	Moderate	Severe	No	Yes
Uncoded [c]	54 (30.5%)	53 (30.5%)	1 (0.6%)	0 (0%)	42 (24.4%)	6 (3.0%)	5 (3.0%)	54 (30.5%)	0 (0%)
Acute on chronic pancreatitis	1 (0.6%)	1 (0.6%)	0 (0%)	0 (0%)	0 (0%)	1 (0.6%)	0 (0%)	1 (0.6%)	0 (0%)
ED appearance for lower GI bleed	1 (0.6%)	1 (0.6%)	0 (0%)	0 (0%)	1 (0.6%)	0 (0%)	0 (0%)	1 (0.6%)	0 (0%)
ED presentation	3 (1.8%)	3 (1.8%)	0 (0%)	0 (0%)	0 (0%)	1 (0.6%)	2 (1.2%)	3 (1.8%)	0 (0%)
ED presentation for SI	1 (0.6%)	1 (0.6%)	0 (0%)	0 (0%)	0 (0%)	0 (0%)	1 (0.6%)	1 (0.6%)	0 (0%)
Hospitalization due to a preexisting condition.	1 (0.6%)	1 (0.6%)	0 (0%)	0 (0%)	0 (0%)	1 (0.6%)	0 (0%)	1 (0.6%)	0 (0%)
Hospitalization for management of chronic abdominal pain.	1 (0.6%)	1 (0.6%)	0 (0%)	0 (0%)	1 (0.6%)	0 (0%)	0 (0%)	1 (0.6%)	0 (0%)
Increased Suicidal Ideation from Baseline	1 (0.6%)	1 (0.6%)	0 (0%)	0 (0%)	0 (0%)	1 (0.6%)	0 (0%)	1 (0.6%)	0 (0%)
Missed Visit	39 (23.8%)	38 (23.2%)	1 (0.6%)	0 (0%)	37 (22.6%)	0 (0%)	2 (1.2%)	39 (23.8%)	0 (0%)
Suicidal Ideation with preparatory actions and intent	1 (0.6%)	1 (0.6%)	0 (0%)	0 (0%)	1 (0.6%)	0 (0%)	0 (0%)	1 (0.6%)	0 (0%)
Suicide attempt 08Jan2024	1 (0.6%)	1 (0.6%)	0 (0%)	0 (0%)	0 (0%)	0 (0%)	0 (0%)	1 (0.6%)	0 (0%)
Suicide attempt via self-strangulation	1 (0.6%)	1 (0.6%)	0 (0%)	0 (0%)	1 (0.6%)	0 (0%)	0 (0%)	1 (0.6%)	0 (0%)
Tracheal Dilation- outpatient procedure	1 (0.6%)	1 (0.6%)	0 (0%)	0 (0%)	0 (0%)	1 (0.6%)	0 (0%)	1 (0.6%)	0 (0%)
Tracheal dilation- outpatient procedure	1 (0.6%)	1 (0.6%)	0 (0%)	0 (0%)	0 (0%)	1 (0.6%)	0 (0%)	1 (0.6%)	0 (0%)
Withdrawal due to study involvement	1 (0.6%)	1 (0.6%)	0 (0%)	0 (0%)	1 (0.6%)	0 (0%)	0 (0%)	1 (0.6%)	0 (0%)
Vascular disorders	2 (1.2%)	2 (1.2%)	0 (0%)	0 (0%)	2 (1.2%)	0 (0%)	0 (0%)	2 (1.2%)	0 (0%)

© 2025 Bryan CJ et al. *JAMA Network Open*

		Related to Device? [a]			Severity [b]			Expected?	
	Total	Definitely No	Possibly	Definitely Yes	Mild	Moderate	Severe	No	Yes
Hypertension	1 (0.6%)	1 (0.6%)	0 (0%)	0 (0%)	1 (0.6%)	0 (0%)	0 (0%)	1 (0.6%)	0 (0%)
Hypotension	1 (0.6%)	1 (0.6%)	0 (0%)	0 (0%)	1 (0.6%)	0 (0%)	0 (0%)	1 (0.6%)	0 (0%)

Note:
- Table entries represent: # of AEs (% of Subjects experiencing the event)
- Participants experiencing an event within a given PT and SOC more than once will be counted under the maximum severity/relationship experienced.

Notes:
- Table entries represent: # of AEs (% of Subjects experiencing the event)
- Participants experiencing an event within a given PT and SOC more than once will be counted under the maximum severity/relationship experienced.

[a] Based on n=197 due to missing data from 1 participant
[b] Based on n=196 due to missing data from 2 participants
[c] Uncoded AEs are as reported by site.

© 2025 Bryan CJ et al. *JAMA Network Open*

eTable 5. Adverse events (AEs) reported by participants assigned to control (n=167)

		Related to Device? [a]			Severity [b]			Expected?	
	Total	Definitely No	Possibly	Definitely Yes	Mild	Moderate	Severe	No	Yes
**Any AEs**	200 (64.1%)	192 (61.7%)	5 (2.4%)	0 (0%)	109 (42.5%)	50 (22.2%)	36 (15.0%)	197 (63.5%)	3 (1.8%)
Cardiac disorders	2 (1.2%)	2 (1.2%)	0 (0%)	0 (0%)	1 (0.6%)	0 (0%)	1 (0.6%)	2 (1.2%)	0 (0%)
Chest pain	2 (1.2%)	2 (1.2%)	0 (0%)	0 (0%)	1 (0.6%)	0 (0%)	1 (0.6%)	2 (1.2%)	0 (0%)
Eye disorders	1 (0.6%)	1 (0.6%)	0 (0%)	0 (0%)	1 (0.6%)	0 (0%)	0 (0%)	1 (0.6%)	0 (0%)
Eye irritation	1 (0.6%)	1 (0.6%)	0 (0%)	0 (0%)	1 (0.6%)	0 (0%)	0 (0%)	1 (0.6%)	0 (0%)
Gastrointestinal disorders	9 (3.6%)	9 (3.6%)	0 (0%)	0 (0%)	4 (2.4%)	3 (1.8%)	2 (0.6%)	9 (3.6%)	0 (0%)
Abdominal pain	2 (1.2%)	2 (1.2%)	0 (0%)	0 (0%)	1 (0.6%)	1 (0.6%)	0 (0%)	2 (1.2%)	0 (0%)
Abdominal pain upper	2 (1.2%)	2 (1.2%)	0 (0%)	0 (0%)	1 (0.6%)	1 (0.6%)	0 (0%)	2 (1.2%)	0 (0%)
Cyclic vomiting syndrome	1 (0.6%)	1 (0.6%)	0 (0%)	0 (0%)	0 (0%)	0 (0%)	1 (0.6%)	1 (0.6%)	0 (0%)
Diarrhoea	1 (0.6%)	1 (0.6%)	0 (0%)	0 (0%)	0 (0%)	1 (0.6%)	0 (0%)	1 (0.6%)	0 (0%)
Gastric ulcer	1 (0.6%)	1 (0.6%)	0 (0%)	0 (0%)	1 (0.6%)	0 (0%)	0 (0%)	1 (0.6%)	0 (0%)
Irritable bowel syndrome	1 (0.6%)	1 (0.6%)	0 (0%)	0 (0%)	1 (0.6%)	0 (0%)	0 (0%)	1 (0.6%)	0 (0%)
Vomiting	1 (0.6%)	1 (0.6%)	0 (0%)	0 (0%)	0 (0%)	0 (0%)	1 (0.6%)	1 (0.6%)	0 (0%)
General disorders and administration site conditions	6 (3.6%)	6 (3.6%)	0 (0%)	0 (0%)	6 (3.6%)	0 (0%)	0 (0%)	6 (3.6%)	0 (0%)
Adverse drug reaction	4 (2.4%)	4 (2.4%)	0 (0%)	0 (0%)	4 (2.4%)	0 (0%)	0 (0%)	4 (2.4%)	0 (0%)
Drug withdrawal syndrome	1 (0.6%)	1 (0.6%)	0 (0%)	0 (0%)	1 (0.6%)	0 (0%)	0 (0%)	1 (0.6%)	0 (0%)
Inflammation	1 (0.6%)	1 (0.6%)	0 (0%)	0 (0%)	1 (0.6%)	0 (0%)	0 (0%)	1 (0.6%)	0 (0%)
Immune system disorders	1 (0.6%)	1 (0.6%)	0 (0%)	0 (0%)	1 (0.6%)	0 (0%)	0 (0%)	1 (0.6%)	0 (0%)
Drug hypersensitivity	1 (0.6%)	1 (0.6%)	0 (0%)	0 (0%)	1 (0.6%)	0 (0%)	0 (0%)	1 (0.6%)	0 (0%)
Infections and infestations	23 (12.0%)	23 (12.0%)	0 (0%)	0 (0%)	17 (8.4%)	5 (3.0%)	1 (0.6%)	23 (12.0%)	0 (0%)

© 2025 Bryan CJ et al. *JAMA Network Open*

		Related to Device? [a]			Severity [b]			Expected?	
	Total	Definitely No	Possibly	Definitely Yes	Mild	Moderate	Severe	No	Yes
Bacterial vaginosis	1 (0.6%)	1 (0.6%)	0 (0%)	0 (0%)	1 (0.6%)	0 (0%)	0 (0%)	1 (0.6%)	0 (0%)
COVID-19	6 (3.6%)	6 (3.6%)	0 (0%)	0 (0%)	6 (3.6%)	0 (0%)	0 (0%)	6 (3.6%)	0 (0%)
Gastroenteritis viral	1 (0.6%)	1 (0.6%)	0 (0%)	0 (0%)	0 (0%)	1 (0.6%)	0 (0%)	1 (0.6%)	0 (0%)
Herpes simplex	1 (0.6%)	1 (0.6%)	0 (0%)	0 (0%)	0 (0%)	1 (0.6%)	0 (0%)	1 (0.6%)	0 (0%)
Influenza	2 (1.2%)	2 (1.2%)	0 (0%)	0 (0%)	1 (0.6%)	0 (0%)	1 (0.6%)	2 (1.2%)	0 (0%)
Nasopharyngitis	1 (0.6%)	1 (0.6%)	0 (0%)	0 (0%)	1 (0.6%)	0 (0%)	0 (0%)	1 (0.6%)	0 (0%)
Norovirus infection	1 (0.6%)	1 (0.6%)	0 (0%)	0 (0%)	1 (0.6%)	0 (0%)	0 (0%)	1 (0.6%)	0 (0%)
Pharyngitis	1 (0.6%)	1 (0.6%)	0 (0%)	0 (0%)	1 (0.6%)	0 (0%)	0 (0%)	1 (0.6%)	0 (0%)
Respiratory syncytial virus infection	1 (0.6%)	1 (0.6%)	0 (0%)	0 (0%)	1 (0.6%)	0 (0%)	0 (0%)	1 (0.6%)	0 (0%)
Rhinovirus infection	1 (0.6%)	1 (0.6%)	0 (0%)	0 (0%)	1 (0.6%)	0 (0%)	0 (0%)	1 (0.6%)	0 (0%)
Sepsis	1 (0.6%)	1 (0.6%)	0 (0%)	0 (0%)	0 (0%)	1 (0.6%)	0 (0%)	1 (0.6%)	0 (0%)
Sinusitis	2 (1.2%)	2 (1.2%)	0 (0%)	0 (0%)	1 (0.6%)	1 (0.6%)	0 (0%)	2 (1.2%)	0 (0%)
Upper respiratory tract infection	1 (0.6%)	1 (0.6%)	0 (0%)	0 (0%)	1 (0.6%)	0 (0%)	0 (0%)	1 (0.6%)	0 (0%)
Urinary tract infection	2 (1.2%)	2 (1.2%)	0 (0%)	0 (0%)	2 (1.2%)	0 (0%)	0 (0%)	2 (1.2%)	0 (0%)
Wound infection	1 (0.6%)	1 (0.6%)	0 (0%)	0 (0%)	0 (0%)	1 (0.6%)	0 (0%)	1 (0.6%)	0 (0%)
Injury, poisoning and procedural complications	14 (6.6%)	14 (6.6%)	0 (0%)	0 (0%)	7 (3.6%)	5 (2.4%)	1 (0.6%)	14 (6.6%)	0 (0%)
Animal bite	1 (0.6%)	1 (0.6%)	0 (0%)	0 (0%)	1 (0.6%)	0 (0%)	0 (0%)	1 (0.6%)	0 (0%)
Arthropod bite	1 (0.6%)	1 (0.6%)	0 (0%)	0 (0%)	0 (0%)	1 (0.6%)	0 (0%)	1 (0.6%)	0 (0%)
Bursitis	1 (0.6%)	1 (0.6%)	0 (0%)	0 (0%)	1 (0.6%)	0 (0%)	0 (0%)	1 (0.6%)	0 (0%)
Fall	1 (0.6%)	1 (0.6%)	0 (0%)	0 (0%)	1 (0.6%)	0 (0%)	0 (0%)	1 (0.6%)	0 (0%)

© 2025 Bryan CJ et al. *JAMA Network Open*

		Related to Device? [a]			Severity [b]			Expected?	
	Total	Definitely No	Possibly	Definitely Yes	Mild	Moderate	Severe	No	Yes
Gastrointestinal stoma complication	1 (0.6%)	1 (0.6%)	0 (0%)	0 (0%)	0 (0%)	0 (0%)	1 (0.6%)	1 (0.6%)	0 (0%)
Joint injury	1 (0.6%)	1 (0.6%)	0 (0%)	0 (0%)	1 (0.6%)	0 (0%)	0 (0%)	1 (0.6%)	0 (0%)
Ligament sprain	2 (1.2%)	2 (1.2%)	0 (0%)	0 (0%)	1 (0.6%)	0 (0%)	0 (0%)	2 (1.2%)	0 (0%)
Road traffic accident	1 (0.6%)	1 (0.6%)	0 (0%)	0 (0%)	0 (0%)	1 (0.6%)	0 (0%)	1 (0.6%)	0 (0%)
Skin laceration	3 (1.8%)	3 (1.8%)	0 (0%)	0 (0%)	1 (0.6%)	2 (1.2%)	0 (0%)	3 (1.8%)	0 (0%)
Tendonitis	1 (0.6%)	1 (0.6%)	0 (0%)	0 (0%)	1 (0.6%)	0 (0%)	0 (0%)	1 (0.6%)	0 (0%)
Thermal burn	1 (0.6%)	1 (0.6%)	0 (0%)	0 (0%)	0 (0%)	1 (0.6%)	0 (0%)	1 (0.6%)	0 (0%)
Investigations	1 (0.6%)	1 (0.6%)	0 (0%)	0 (0%)	1 (0.6%)	0 (0%)	0 (0%)	1 (0.6%)	0 (0%)
Blood urine present	1 (0.6%)	1 (0.6%)	0 (0%)	0 (0%)	1 (0.6%)	0 (0%)	0 (0%)	1 (0.6%)	0 (0%)
Metabolism and nutrition disorders	2 (1.2%)	2 (1.2%)	0 (0%)	0 (0%)	1 (0.6%)	0 (0%)	1 (0.6%)	2 (1.2%)	0 (0%)
Hypoglycaemia	1 (0.6%)	1 (0.6%)	0 (0%)	0 (0%)	0 (0%)	0 (0%)	1 (0.6%)	1 (0.6%)	0 (0%)
Vitamin D deficiency	1 (0.6%)	1 (0.6%)	0 (0%)	0 (0%)	1 (0.6%)	0 (0%)	0 (0%)	1 (0.6%)	0 (0%)
Musculoskeletal and connective tissue disorders	3 (1.8%)	3 (1.8%)	0 (0%)	0 (0%)	0 (0%)	3 (1.8%)	0 (0%)	3 (1.8%)	0 (0%)
Ankle fracture	1 (0.6%)	1 (0.6%)	0 (0%)	0 (0%)	0 (0%)	1 (0.6%)	0 (0%)	1 (0.6%)	0 (0%)
Arthralgia	2 (1.2%)	2 (1.2%)	0 (0%)	0 (0%)	0 (0%)	2 (1.2%)	0 (0%)	2 (1.2%)	0 (0%)
Nervous system disorders	13 (7.2%)	12 (6.6%)	0 (0%)	0 (0%)	7 (4.2%)	4 (1.8%)	1 (0.6%)	13 (7.2%)	0 (0%)
Dizziness	1 (0.6%)	1 (0.6%)	0 (0%)	0 (0%)	0 (0%)	0 (0%)	1 (0.6%)	1 (0.6%)	0 (0%)
Headache	4 (2.4%)	3 (1.8%)	0 (0%)	0 (0%)	3 (1.8%)	0 (0%)	0 (0%)	4 (2.4%)	0 (0%)
Hypoaesthesia	1 (0.6%)	1 (0.6%)	0 (0%)	0 (0%)	1 (0.6%)	0 (0%)	0 (0%)	1 (0.6%)	0 (0%)
Migraine	3 (1.8%)	3 (1.8%)	0 (0%)	0 (0%)	1 (0.6%)	2 (1.2%)	0 (0%)	3 (1.8%)	0 (0%)
Myoclonic epilepsy	1 (0.6%)	1 (0.6%)	0 (0%)	0 (0%)	1 (0.6%)	0 (0%)	0 (0%)	1 (0.6%)	0 (0%)

© 2025 Bryan CJ et al. *JAMA Network Open*

		Related to Device? [a]			Severity [b]			Expected?	
	Total	Definitely No	Possibly	Definitely Yes	Mild	Moderate	Severe	No	Yes
Seizure	2 (1.2%)	2 (1.2%)	0 (0%)	0 (0%)	1 (0.6%)	1 (0.6%)	0 (0%)	2 (1.2%)	0 (0%)
Tremor	1 (0.6%)	1 (0.6%)	0 (0%)	0 (0%)	0 (0%)	1 (0.6%)	0 (0%)	1 (0.6%)	0 (0%)
Pregnancy, puerperium and perinatal conditions	1 (0.6%)	1 (0.6%)	0 (0%)	0 (0%)	0 (0%)	1 (0.6%)	0 (0%)	1 (0.6%)	0 (0%)
Abortion spontaneous	1 (0.6%)	1 (0.6%)	0 (0%)	0 (0%)	0 (0%)	1 (0.6%)	0 (0%)	1 (0.6%)	0 (0%)
Product issues	1 (0.6%)	1 (0.6%)	0 (0%)	0 (0%)	1 (0.6%)	0 (0%)	0 (0%)	1 (0.6%)	0 (0%)
Embedded device	1 (0.6%)	1 (0.6%)	0 (0%)	0 (0%)	1 (0.6%)	0 (0%)	0 (0%)	1 (0.6%)	0 (0%)
Psychiatric disorders	51 (23.4%)	47 (22.8%)	4 (1.8%)	0 (0%)	15 (7.8%)	16 (9.0%)	19 (9.6%)	49 (22.8%)	2 (1.2%)
Anxiety	5 (3.0%)	4 (2.4%)	1 (0.6%)	0 (0%)	4 (2.4%)	1 (0.6%)	0 (0%)	4 (2.4%)	1 (0.6%)
Bipolar I disorder	1 (0.6%)	1 (0.6%)	0 (0%)	0 (0%)	1 (0.6%)	0 (0%)	0 (0%)	1 (0.6%)	0 (0%)
Bipolar II disorder	1 (0.6%)	1 (0.6%)	0 (0%)	0 (0%)	1 (0.6%)	0 (0%)	0 (0%)	1 (0.6%)	0 (0%)
Borderline personality disorder	1 (0.6%)	1 (0.6%)	0 (0%)	0 (0%)	1 (0.6%)	0 (0%)	0 (0%)	1 (0.6%)	0 (0%)
Completed suicide	1 (0.6%)	1 (0.6%)	0 (0%)	0 (0%)	0 (0%)	0 (0%)	1 (0.6%)	1 (0.6%)	0 (0%)
Hypomania	1 (0.6%)	1 (0.6%)	0 (0%)	0 (0%)	1 (0.6%)	0 (0%)	0 (0%)	1 (0.6%)	0 (0%)
Intentional self-injury	3 (1.8%)	2 (1.2%)	1 (0.6%)	0 (0%)	1 (0.6%)	2 (1.2%)	0 (0%)	3 (1.8%)	0 (0%)
Major depression	1 (0.6%)	1 (0.6%)	0 (0%)	0 (0%)	1 (0.6%)	0 (0%)	0 (0%)	1 (0.6%)	0 (0%)
Panic attack	2 (1.2%)	2 (1.2%)	0 (0%)	0 (0%)	0 (0%)	2 (1.2%)	0 (0%)	2 (1.2%)	0 (0%)
Psychotic disorder	1 (0.6%)	1 (0.6%)	0 (0%)	0 (0%)	0 (0%)	1 (0.6%)	0 (0%)	1 (0.6%)	0 (0%)
Restlessness	1 (0.6%)	1 (0.6%)	0 (0%)	0 (0%)	1 (0.6%)	0 (0%)	0 (0%)	1 (0.6%)	0 (0%)
Suicidal ideation	16 (9.6%)	15 (9.0%)	1 (0.6%)	0 (0%)	3 (1.8%)	6 (3.6%)	6 (3.6%)	15 (9.0%)	1 (0.6%)
Suicide attempt	17 (10.2%)	16 (9.6%)	1 (0.6%)	0 (0%)	1 (0.6%)	4 (2.4%)	12 (7.2%)	17 (10.2%)	0 (0%)
Reproductive system and breast disorders	3 (1.2%)	3 (1.2%)	0 (0%)	0 (0%)	1 (0.6%)	0 (0%)	2 (0.6%)	3 (1.2%)	0 (0%)

© 2025 Bryan CJ et al. *JAMA Network Open*

		Related to Device? [a]			Severity [b]			Expected?	
	Total	Definitely No	Possibly	Definitely Yes	Mild	Moderate	Severe	No	Yes
Ovarian cyst	1 (0.6%)	1 (0.6%)	0 (0%)	0 (0%)	1 (0.6%)	0 (0%)	0 (0%)	1 (0.6%)	0 (0%)
Ovulation pain	1 (0.6%)	1 (0.6%)	0 (0%)	0 (0%)	0 (0%)	0 (0%)	1 (0.6%)	1 (0.6%)	0 (0%)
Pelvic pain	1 (0.6%)	1 (0.6%)	0 (0%)	0 (0%)	0 (0%)	0 (0%)	1 (0.6%)	1 (0.6%)	0 (0%)
Respiratory, thoracic and mediastinal disorders	1 (0.6%)	1 (0.6%)	0 (0%)	0 (0%)	1 (0.6%)	0 (0%)	0 (0%)	1 (0.6%)	0 (0%)
Nasal congestion	1 (0.6%)	1 (0.6%)	0 (0%)	0 (0%)	1 (0.6%)	0 (0%)	0 (0%)	1 (0.6%)	0 (0%)
Skin and subcutaneous tissue disorders	5 (3.0%)	5 (3.0%)	0 (0%)	0 (0%)	3 (1.8%)	1 (0.6%)	1 (0.6%)	5 (3.0%)	0 (0%)
Dermatitis	1 (0.6%)	1 (0.6%)	0 (0%)	0 (0%)	0 (0%)	0 (0%)	1 (0.6%)	1 (0.6%)	0 (0%)
Granuloma skin	1 (0.6%)	1 (0.6%)	0 (0%)	0 (0%)	0 (0%)	1 (0.6%)	0 (0%)	1 (0.6%)	0 (0%)
Rash	1 (0.6%)	1 (0.6%)	0 (0%)	0 (0%)	1 (0.6%)	0 (0%)	0 (0%)	1 (0.6%)	0 (0%)
Scratch	1 (0.6%)	1 (0.6%)	0 (0%)	0 (0%)	1 (0.6%)	0 (0%)	0 (0%)	1 (0.6%)	0 (0%)
Urticaria	1 (0.6%)	1 (0.6%)	0 (0%)	0 (0%)	1 (0.6%)	0 (0%)	0 (0%)	1 (0.6%)	0 (0%)
Surgical and medical procedures	3 (1.8%)	3 (1.8%)	0 (0%)	0 (0%)	0 (0%)	2 (1.2%)	1 (0.6%)	2 (1.2%)	1 (0.6%)
Colostomy	1 (0.6%)	1 (0.6%)	0 (0%)	0 (0%)	0 (0%)	1 (0.6%)	0 (0%)	1 (0.6%)	0 (0%)
Hospitalisation	2 (1.2%)	2 (1.2%)	0 (0%)	0 (0%)	0 (0%)	1 (0.6%)	1 (0.6%)	1 (0.6%)	1 (0.6%)
Uncoded [c]	56 (28.7%)	53 (26.9%)	1 (0.6%)	0 (0%)	40 (21.6%)	8 (4.2%)	6 (3.6%)	56 (28.7%)	0 (0%)
COVID	1 (0.6%)	1 (0.6%)	0 (0%)	0 (0%)	1 (0.6%)	0 (0%)	0 (0%)	1 (0.6%)	0 (0%)
Crohn's disease exacerbation	1 (0.6%)	1 (0.6%)	0 (0%)	0 (0%)	0 (0%)	1 (0.6%)	0 (0%)	1 (0.6%)	0 (0%)
ED Visit for Viral Gastroenteritis	1 (0.6%)	1 (0.6%)	0 (0%)	0 (0%)	0 (0%)	1 (0.6%)	0 (0%)	1 (0.6%)	0 (0%)

© 2025 Bryan CJ et al. *JAMA Network Open*

		Related to Device? [a]			Severity [b]			Expected?	
	Total	Definitely No	Possibly	Definitely Yes	Mild	Moderate	Severe	No	Yes
ED appearance and subsequent psychiatric hospitalization due to increased suicidal ideation and aborted/interrupted attempts	1 (0.6%)	1 (0.6%)	0 (0%)	0 (0%)	0 (0%)	0 (0%)	1 (0.6%)	1 (0.6%)	0 (0%)
ED appearance for alcohol intoxication and potential psychiatric crisis	1 (0.6%)	1 (0.6%)	0 (0%)	0 (0%)	0 (0%)	1 (0.6%)	0 (0%)	1 (0.6%)	0 (0%)
ED appearance for dizziness	1 (0.6%)	1 (0.6%)	0 (0%)	0 (0%)	1 (0.6%)	0 (0%)	0 (0%)	1 (0.6%)	0 (0%)
ED presentation	2 (1.2%)	2 (1.2%)	0 (0%)	0 (0%)	0 (0%)	1 (0.6%)	1 (0.6%)	2 (1.2%)	0 (0%)
ED visit and subsequent psychiatric admission for suicidal ideation	1 (0.6%)	1 (0.6%)	0 (0%)	0 (0%)	0 (0%)	0 (0%)	1 (0.6%)	1 (0.6%)	0 (0%)
Low Vitamin D Level	1 (0.6%)	1 (0.6%)	0 (0%)	0 (0%)	1 (0.6%)	0 (0%)	0 (0%)	1 (0.6%)	0 (0%)
Missed Visit	36 (21.6%)	35 (21.0%)	0 (0%)	0 (0%)	32 (19.2%)	1 (0.6%)	2 (1.2%)	36 (21.6%)	0 (0%)
Non suicidal self harm (cutting) needing sutures.	1 (0.6%)	0 (0%)	1 (0.6%)	0 (0%)	0 (0%)	1 (0.6%)	0 (0%)	1 (0.6%)	0 (0%)
Other	1 (0.6%)	0 (0%)	0 (0%)	0 (0%)	0 (0%)	0 (0%)	0 (0%)	1 (0.6%)	0 (0%)
Risk Concern - Per Outpatient Provider	1 (0.6%)	1 (0.6%)	0 (0%)	0 (0%)	1 (0.6%)	0 (0%)	0 (0%)	1 (0.6%)	0 (0%)
Urgent care appearance for Dizziness	1 (0.6%)	1 (0.6%)	0 (0%)	0 (0%)	1 (0.6%)	0 (0%)	0 (0%)	1 (0.6%)	0 (0%)
Urgent care appearance for abdominal pain	1 (0.6%)	1 (0.6%)	0 (0%)	0 (0%)	0 (0%)	1 (0.6%)	0 (0%)	1 (0.6%)	0 (0%)
Urgent care visit for back/arm pain	1 (0.6%)	1 (0.6%)	0 (0%)	0 (0%)	1 (0.6%)	0 (0%)	0 (0%)	1 (0.6%)	0 (0%)
Urgent care visit for upper respiratory symptoms	1 (0.6%)	1 (0.6%)	0 (0%)	0 (0%)	1 (0.6%)	0 (0%)	0 (0%)	1 (0.6%)	0 (0%)

© 2025 Bryan CJ et al. *JAMA Network Open*

		Related to Device? [a]			Severity [b]			Expected?	
	Total	Definitely No	Possibly	Definitely Yes	Mild	Moderate	Severe	No	Yes
left ankle pain	1 (0.6%)	1 (0.6%)	0 (0%)	0 (0%)	1 (0.6%)	0 (0%)	0 (0%)	1 (0.6%)	0 (0%)
suicide attempt via ataraxic ingestion	1 (0.6%)	1 (0.6%)	0 (0%)	0 (0%)	0 (0%)	0 (0%)	1 (0.6%)	1 (0.6%)	0 (0%)
worsening anxiety	1 (0.6%)	1 (0.6%)	0 (0%)	0 (0%)	0 (0%)	1 (0.6%)	0 (0%)	1 (0.6%)	0 (0%)
Vascular disorders	4 (2.4%)	4 (2.4%)	0 (0%)	0 (0%)	2 (1.2%)	2 (1.2%)	0 (0%)	4 (2.4%)	0 (0%)
Haematochezia	1 (0.6%)	1 (0.6%)	0 (0%)	0 (0%)	0 (0%)	1 (0.6%)	0 (0%)	1 (0.6%)	0 (0%)
Peripheral coldness	1 (0.6%)	1 (0.6%)	0 (0%)	0 (0%)	1 (0.6%)	0 (0%)	0 (0%)	1 (0.6%)	0 (0%)
Rectal haemorrhage	1 (0.6%)	1 (0.6%)	0 (0%)	0 (0%)	0 (0%)	1 (0.6%)	0 (0%)	1 (0.6%)	0 (0%)
Vaginal haemorrhage	1 (0.6%)	1 (0.6%)	0 (0%)	0 (0%)	1 (0.6%)	0 (0%)	0 (0%)	1 (0.6%)	0 (0%)

Note:
- Table entries represent: # of AEs (% of Subjects experiencing the event)
- Participants experiencing an event within a given PT and SOC more than once will be counted under the maximum severity/relationship experienced.
[a] Based on n=197 due to missing data from 3 participants
[b] Based on n=195 due to missing data from 5 participants
[c] Uncoded AEs are as reported by site.

© 2025 Bryan CJ et al. *JAMA Network Open*
