# Supplementary material for: A Digital Therapeutic Intervention for Inpatients With Elevated Suicide Risk: A Randomized Clinical Trial
Source: JAMA Netw Open. 2025 Aug 8;8(8):e2525809. doi: 10.1001/jamanetworkopen.2025.25809 (PMC12334960; doi:10.1001/jamanetworkopen.2025.25809)
Supplement: Supplement 3. — Data Sharing Statement [file jamanetwopen-e2525809-s003.pdf]

Data Sharing Statement

Bryan. A Digital Therapeutic Intervention for Inpatients With Elevated Suicide Risk. *JAMA Netw Open*. Published August 08, 2025. doi:10.1001/jamanetworkopen.2025.25809

Data
Additional Information: clinicaltrials.gov, https://clinicaltrials.gov/study/NCT05144685, NCT05144685
Data available: Yes
Data types: Deidentified participant data
How to access data: patricia.simon@yale.edu
When available: With publication

Supporting Documents
Document types: Informed consent form
How to access documents: https://clinicaltrials.gov/study/NCT05144685?term=otx-202&rank=1
When available: With publication

Additional Information
Who can access the data: Researchers whose proposed use of the data has been approved
Types of analyses: for a specified purpose
Mechanisms of data availability: with investigator support after approval of a proposal and with a signed data access agreement
